# Effectiveness of Digital Health Interventions in Older Adults With Frailty and Sarcopenia: Systematic Review and Meta‐Analysis of Randomized Controlled Trials

**DOI:** 10.2196/88374

**Published:** 2026-05-11

**Authors:** Ting Dai, Changsheng Guo, Lingli Gao, Jinyu Huang, Yan Chen, Yujie Zhang, Jing Gao, Xiaodong Feng

**Affiliations:** 1 Rehabilitation Center, The First Affiliated Hospital of Henan University of Traditional Chinese Medicine, No.19 Renmin Road, Zhengzhou, Henan, 450099, China, +86 371 6621 2349; 2 The First Clinical Medical College, Henan University of Chinese Medicine, Zhengzhou, China; 3 Rehabilitation Center, The Second Affiliated Hospital of Heilongjiang University of Chinese Medicine, Harbin, Heilongjiang, China; 4 The First Affiliated Hospital of Henan University of CM, Heart Center/National Regional (Traditional Chinese Medicine) Cardiovascular Diagnosis and Treatment Center, Zhengzhou, Henan, China

**Keywords:** digital health intervention, sarcopenia, frailty, older adults, meta-analysis

## Abstract

**Background:**

Frailty and sarcopenia represent substantial global health challenges, frequently diminishing patients’ quality of life through impaired muscle function and physical performance. Digital health interventions (DHIs) have shown promise in mitigating these conditions among older adults. However, outcomes of such interventions in this demographic are inconsistent, and a thorough synthesis of existing evidence is lacking.

**Objective:**

This study aimed to evaluate the effectiveness of DHIs in older adults with frailty and sarcopenia.

**Methods:**

A comprehensive search of PubMed, Web of Science, MEDLINE, Embase, and Cochrane Library was conducted from their inception until January 2026 to identify randomized controlled trials. Meta-analyses were performed using R software (R Foundation for Statistical Computing). Study quality was evaluated using the revised Cochrane Risk of Bias Tool 2.0 (Cochrane Collaboration), and evidence certainty was assessed using GRADE (Grading of Recommendations, Assessment, Development, and Evaluation).

**Results:**

From 3506 records, 16 studies were included. DHIs significantly improved total skeletal muscle mass (weighted mean difference [WMD] 1.01, 95% CI 0.08-1.94, 95% prediction interval [PI] −0.95 to 2.96), gait speed (WMD 0.09, 95% CI 0.03-0.15, 95% PI −0.1 to 0.26), Timed Up and Go Test (TUGT: WMD −0.52, 95% CI −1.02 to −0.03, 95% PI −1.93 to 0.85), 30-second Chair Stand Test (30CST: WMD 2.19, 95% CI 0.89-3.48, 95% PI −1.59 to 5.66), balance (standardized mean difference [SMD] 0.61, 95% CI 0-1.21, 95% PI −0.94 to 2.13), and quality of life (SMD 0.16, 95% CI 0.05-0.27, 95% PI 0.04-0.28). No significant improvements were observed in Appendicular Skeletal Muscle Mass Index (ASMI), grip strength, 6-minute walk test (6MWT), 2-minute walk test (2MWT), Short Physical Performance Battery (SPPB), or BMI. Although the pooled effect was favorable, the wide 95% PI suggests substantial between-study heterogeneity. Subgroup analysis stratified by intervention duration revealed significant intersubgroup differences in ASMI (*χ*²_₁_=9.93; *P*=.0016), indicating interventions lasting ≥12 weeks were more effective for improving ASMI (WMD 0.28, 95% CI 0.06-0.50, 95% PI −0.30 to 0.83). Subgroup analysis stratified by intervention type showed significant intersubgroup differences in balance (*χ*²_₃_=9.89; *P*=.0195), with exergame-based interventions showing significant effects (SMD 0.83, 95% CI 0.26-1.40).

**Conclusions:**

This systematic review is the first to quantify the disease-specific efficacy of DHIs in improving muscle function, physical performance, and quality of life among older adults with frailty and sarcopenia, demonstrating their unique value as a scalable complementary approach. By overcoming geographical and resource constraints, DHIs support underserved populations. However, low evidence quality and heterogeneity warrant cautious interpretation. The 95% PIs indicate that actual effects may vary with population characteristics and implementation contexts. Nonetheless, DHIs represent a promising and cost-effective strategy for service expansion. Future high-quality studies are needed to better understand their effectiveness and implementation across settings.

## Introduction

With the accelerating trend of global population aging, frailty and sarcopenia have emerged as significant public health challenges that are closely associated with geriatric syndromes [1]. Frailty is a multidimensional syndrome characterized by decreased functional reserve and increased vulnerability to stressors [2]. Sarcopenia, defined as the loss of skeletal muscle mass and function, typically precedes frailty and is a core component of frailty [2,3]. A shared pathophysiological hallmark and a key clinical link between these 2 conditions is the presence of muscle atrophy and the consequent decline in physical performance [4,5]. Systematic reviews and meta-analyses indicate that the prevalence of frailty among people aged 60 years and older is 16%, while the prevalence of prefrailty is as high as 47% [6]. The prevalence of sarcopenia ranges from 10% to 27% [7]. Critically, both of these highly prevalent conditions are important predictors of serious adverse health outcomes [8-10], profoundly impacting older adults’ function, quality of life, and survival of older adults [5,11]. Despite sufficient evidence indicating that moderate-to-high intensity resistance training effectively enhances muscle function and physical performance in older adults [12], the face-to-face implementation of this intervention is constrained by practical factors such as inadequate health system resources [13], heavy burden of travel for medical care [14], and age-related physical decline in older adults themselves [15]. These factors have significantly reduced patient adherence and motivation [16], highlighting an urgent need for innovative management strategies.

In recent years, the rapid advancement of digital technologies in the health care field has brought transformative opportunities for optimizing health interventions and strengthening health systems [17].

Digital health interventions (DHIs), leveraging technologies such as telemedicine, mobile apps, wearable devices, exergames, and virtual reality, can provide support for comprehensive health management in older adults [18,19]. They enable health monitoring, personalized health education, remote interventions, and continuous surveillance [19-21], holding great promise for overcoming economic, geographical, temporal, and physical barriers. Notably, the COVID-19 pandemic has accelerated the integration and adoption of digital processes within health care globally [22].

Multiple clinical studies have demonstrated that DHIs, such as wearable devices [23], exergaming [24], and telephone reminders [25], show potential in improving functional outcomes among older adults with frailty and sarcopenia. However, their implementation and scaling face dual challenges, as follows: (1) at the system level, structural disparities in health care resource allocation, uneven regional distribution of medical device networks, and insufficient digital transformation capacity may hinder the dissemination and adoption of advanced technologies, potentially exacerbating geographic inequities in health care access [26]; and (2) at the evidence level, significant heterogeneity in study designs and methodologies has led to inconsistent conclusions regarding efficacy and a lack of consensus, thereby further limiting the clinical translation of these evidence-based findings.

Although multiple reviews have systematically summarized the effectiveness of DHIs in older adults with frailty or sarcopenia [27-32], to the best of our knowledge, considerable methodological and content gaps still exist in the existing literature. First, at the study population level, current reviews focus on either frail or sarcopenic individuals as a single group, conducting pooled effect analyses for only a single disease state, and thus fail to adequately account for the fact that frailty and sarcopenia are distinct but interrelated conditions [33], resulting in conclusions that inadequately cover this high-risk population and lack clinical applicability. Second, regarding the consistency of evidence, inconsistent findings have been reported; for instance, one review demonstrated that digital technology interventions significantly improved grip strength [27], whereas another study observed no such beneficial effect [32]. Meanwhile, the narrow range of intervention types [28,31], the absence of long-term follow-up assessments [27-32], and the limited inclusion of core outcomes covering muscle function, physical function, and quality of life further restrict the comprehensive evaluation of intervention effects [28,30,31,34]. In addition, the moderating effects of key intervention parameters (eg, intervention duration and delivery mode) on efficacy have not been systematically analyzed.

These evidence gaps hinder the development of rehabilitation strategies for patients with frailty and sarcopenia. Therefore, this meta-analysis aims to (1) systematically evaluate the effectiveness of DHIs on muscle function (muscle mass and strength), physical function, and quality of life in older adults with frailty and sarcopenia; and (2) through subgroup analyses, explore the influences of potential moderators including intervention type, duration, control group design, delivery and synchronization modes, and follow-up effects, with the goal of providing clearer evidence-based guidance for clinical practice and future research.

## Methods

### Protocol and Registration

This systematic review was conducted in accordance with the PRISMA (Preferred Reporting Items for Systematic Reviews and Meta-Analyses) guidelines [35], using the PRISMA 2020 checklist (Checklist 1). Additionally, the study protocol was registered with the International Prospective Register of Systematic Reviews (CRD420251135302).

### Data Sources and Search Strategy

This systematic search was conducted in strict accordance with the PRISMA-S (Preferred Reporting Items for Systematic Reviews and Meta-Analyses search extension) guidelines [36]. A systematic search was performed across 5 major databases: PubMed (NCBI), Embase, Cochrane Library (Wiley), Web of Science, and Ovid MEDLINE. The search encompassed the period from the inception of each database to July 10, 2025, with no restrictions imposed on language or publication date. To ensure the inclusion of the most recent evidence, the search was updated on January 9, 2026. This updated search featured an optimized combination of MeSH (Medical Subject Headings) and keywords, which was informed by previous studies [27,29,30] and tailored to the characteristics of each database, thereby yielding search strategies that differed from those used initially. It should be noted that the search strategies used have not been formally peer-reviewed.

To enhance the comprehensiveness of the retrieval process, the reference lists of pertinent systematic reviews and their included studies were meticulously reviewed. In instances where essential data were absent from the included studies, we reached out to the corresponding authors via email to request the unreported yet crucial original data. The literature search was restricted to the methodologies outlined above, and gray literature, unpublished studies, and preprints were not systematically explored. All database searches were completed on January 11, 2026. Comprehensive search strategies for each database are documented in Multimedia Appendix 1.

### Eligibility Criteria

Two authors independently conducted the screening and selection of studies according to predefined criteria. Any discrepancies encountered were resolved through discussion or, if necessary, by consulting a third reviewer. The determination of study eligibility was guided by the PICOS (Population, Intervention, Comparison, Outcomes, and Study Design) framework.

The inclusion criteria were as follows: (1) participants were aged ≥60 years and carried a diagnosis of frailty or sarcopenia, established according to specified diagnostic criteria. (2) The intervention implemented was a DHI delivered via digital platforms or technologies, through one or more modalities, including but not limited to telephone, video, mobile apps, or wearable devices. (3) Control groups received nondigital interventions, including but not limited to usual care, waitlist, active control, or blank control. (4) The outcomes associated with frailty and sarcopenia were evaluated through comprehensive assessments of muscle strength, muscle mass, physical performance, BMI, and quality of life. Specifically, muscle mass measurements included skeletal muscle mass and the Appendicular Skeletal Muscle Mass Index (ASMI). Physical performance was assessed using various performance-based tests, including gait speed, the 6-Minute Walk Test (6MWT), the 2-Minute Walk Test (2MWT), the Five Times Sit to Stand Test (FTSST), the 30-Second Chair Stand Test (30CST), the Timed Up and Go Test (TUGT), the Short Physical Performance Battery (SPPB), and balance assessments. These outcomes were systematically measured at both baseline and the study end point using objective and subjective instruments, and they were classified as either primary or secondary outcomes. (5) The study design was randomized controlled trials (RCTs). To enhance the inclusiveness and generalizability of the review findings, we removed the original restriction of a minimum of 10 participants per intervention group, thereby avoiding unnecessary exclusion of small but methodologically rigorous RCTs that can provide valuable evidence for the evidence synthesis.

The exclusion criteria were as follows: (1) the study population was not explicitly defined as frail (using established criteria such as the Clinical Frailty Scale [37], Fried Phenotype [2], etc) or sarcopenic (using diagnostic guidelines such as EWGSOP (European Working Group on Sarcopenia in Older People) [5], AWGS (Asian Working Group for Sarcopenia) [38], etc), and participants were aged younger than 60 years. (2) Studies using digital devices only for passive monitoring without a structured intervention. (3) The control group received DHIs. (4) No quantitative outcomes related to frailty or sarcopenia (eg, muscle strength, muscle mass, physical performance, and activities of daily living) were reported. (5) The data were incomplete or were presented in a format (eg, medians without IQRs) that made it impossible to calculate effect sizes. (6) Reviews, commentaries, news, conference abstracts, case reports, conference papers, or unpublished manuscripts

### Study Selection and Data Extraction

The literature screening was conducted using EndNote software (Clarivate). Two researchers initially screened articles by reviewing titles and abstracts, removing duplicates. If relevance could not be determined from the title or abstract, the full text was assessed. All disagreements were resolved through discussion, with the corresponding author making the final decision when consensus was not reached.

We strictly adhered to the guidelines outlined in the *Cochrane Handbook for Systematic Reviews of Interventions*, designed and optimized a structured data extraction form, and validated its reliability and reproducibility through preextraction in a subset of included studies. Subsequently, 2 independent researchers extracted the following information from all included studies: basic study characteristics (first author, year of publication, country or countries in which the studies were conducted, and study design), disease and diagnostic details (patient population and diagnostic criteria), participant characteristics (sample size, age range, and gender distribution), intervention and control specifics (type of digital health technology, control intervention, and duration of intervention), as well as outcome measures and intention-to-treat (ITT) analysis. Upon completion of data extraction, a third researcher verified the extracted data.

Data extraction followed the predefined criteria below: (1) for studies with multiple publications, the most recent version with the most comprehensive methodological descriptions and outcome reports was prioritized. (2) For multiarm RCTs, data from each intervention arm were extracted independently. (3) In case of missing key outcome data, the corresponding author of the original study was first contacted via email to attempt data acquisition. If data remained unavailable, the study was excluded from subsequent meta-analyses. (4) Immediate data from the first assessment after intervention completion were prioritized for extraction, with supplementary follow-up data retrieved to support comparative analyses. (5) For studies reporting both ITT analysis and per-protocol analysis, the former was used as the primary data source. (6) When the original literature did not directly report means and SDs, we used meta-analytical tools for data estimation and conversion to perform the necessary transformations or calculations [39]. All extracted data underwent final review by a third researcher.

Additionally, to systematically present the diversity of digital health interventions, 2 researchers classified the interventions using an inductive approach based on the full-text content of the included studies. Discrepancies arising during the classification process were resolved through consensus after discussions involving a third researcher.

### Risk of Bias Assessment

Risk of bias was assessed using the revised Cochrane Risk of Bias tool (RoB 2.0), which covers 5 domains: randomization, deviations from interventions, missing data, outcome measurement, and result selection. Two reviewers independently assessed studies, resolving disagreements via discussion. Overall risk of bias was rated as low, some concerns, or high: low if all domains were low risk; some concerns if at least one domain had concerns and none were high risk; high if any domain was high risk or multiple domains had concerns.

### Quality of Evidence Evaluation

The quality of evidence for each meta-analysis was appraised using the Grading of Recommendations, Assessment, Development and Evaluations (GRADE) approach [40]. Evidence quality was evaluated based on 5 downgrading factors: risk of bias, inconsistency, indirectness, imprecision, and publication bias, as well as 3 upgrading factors: effect size, impact of plausible confounding, and dose-response relationship. Evidence quality was categorized into 4 levels: high, moderate, low, and very low. Two authors independently performed the GRADE assessment with the GRADEpro GDT tool (GRADE Working Group), with discrepancies resolved by a third author.

### Data Synthesis and Analysis

Although the original protocol planned to use Review Manager 5.3 software (Cochrane Collaboration) and model selection based on *I*² statistic and Q test *P* values, the final data analysis was performed in RStudio (Posit, PBC). A conservative random-effects model, grounded in conceptual assumptions regarding between-study heterogeneity, was used. Effect sizes were pooled using the Hartung-Knapp-Sidik-Jonkman (HKSJ) method [41]. To strengthen methodological rigor and clinical interpretability, additional analytical methods beyond the original protocol were implemented during the analytical refinement phase, including 95% prediction intervals (PIs) and heterogeneity quantification using τ².

This systematic review calculated pooled effect sizes using the changes in means and SDs from pre- to postintervention, with the SD of change scores estimated using the equations specified in the *Cochrane Handbook for Systematic Reviews of Interventions*. The calculations were performed as follows:


$${\text{Mean}}_{\text{change}}={\text{Mean}}_{\text{post}}-{\text{Mean}}_{\text{pre}}$$

$${SD}_{\text{change}}=\sqrt{{SD}_{\text{pre}}^{2}+{SD}_{\text{post}}^{2}-2\times r\times {SD}_{\text{pre}}\times {SD}_{\text{post}}}$$

Here, *r* represents the correlation coefficient between pre- and postintervention values. If this correlation coefficient was not reported in the original studies, a value of *r*=0.5 was assumed, consistent with the Handbook’s recommendations. When studies provided only between-group differences, the corresponding authors were contacted to obtain within-group change data. In instances where SEs were reported instead of SD, the SD was calculated using the formula:

SD=SE×$\sqrt{n}$

When data were reported as median (IQR), the mean and SD were derived using the sample size–based method described by Hozo et al [42]. Studies that did not report SD, SE, or 95% CI and for which the corresponding authors did not provide these values upon request were excluded from the meta-analysis. Additionally, when outcomes were stratified by sex, age, or body weight status, they were analyzed separately by subgroup.

For outcome measures assessed with identical instruments and units, the weighted mean difference (WMD) was applied to pool effect estimates; for those evaluated using different instruments or scales, the standardized mean difference (SMD) was used to ensure comparability of effect sizes. Given the predominantly small sample sizes across the included studies, Hedges *g* was selected as the estimator for SMD to correct for small sample bias. Following the Cochrane Handbook, SMD values of 0.2, 0.5, and 0.8 were interpreted as representing small, medium, and large effects, respectively. For multiarm trials, the shared control group was split into approximately equal subgroups, with one subgroup assigned to each intervention arm for comparison, thereby avoiding double counting of participants and preventing unit-of-analysis errors [43,44]. For cluster RCTs, we first evaluated whether the original studies used appropriate statistical methods to account for intracluster correlation [45]. Where such appropriate adjustments were absent from the original reports, an inflated SE approach was applied to approximate a corrected analysis.

To quantify the real-world variability of the intervention effect across different populations and application scenarios, the study also reports PIs [46]. Because conventional PIs perform poorly when the number of studies is small or heterogeneity is low, we used the bootstrap method proposed by Nagashima et al [47] to calculate PIs. This approach draws samples of the heterogeneity parameter τ² from the exact distribution of the Cochran Q statistic and combines them with the Hartung-Knapp SE to construct a predictive distribution, thereby improving the robustness of PI estimation and inference even when the number of studies is very limited (K<5) or heterogeneity is moderate. In addition, the Minimal Clinically Important Difference (MCID) reported in previous literature in the field was referenced to evaluate the clinical relevance of the estimated effects.

Given the noticeable differences among the included studies in participant characteristics, intervention protocols, and adherence, a conservative random effects model was chosen based on the conceptual assumption of a distribution of true effects [48]. For effect size pooling, following the recommendation of the Cochrane Collaboration, we used the HKSJ method for random-effects meta-analysis. By adopting a distribution and adjusting the SE with the Q statistic, the HKSJ approach yields more robust CIs for the effect size compared with the conventional DerSimonian-Laird method, especially when the number of included studies is small and statistical power is limited; it thus better accounts for the inherent uncertainty and imprecision in effect estimation [41]. Restricted maximum likelihood estimation was used to estimate the between-study variance τ² so as to more accurately reflect the impact of heterogeneity on the effect estimates. Heterogeneity was assessed using the Q-test, *I*² statistic, and τ² metric [49]. When 3 or more studies are included, we also report 95% PIs to indicate the expected distribution range of effect sizes in new settings. The *I*² statistic is reported for comparability with other studies but is interpreted with caution [49,50]. All statistical analyses were performed using R software (version 4.5.2; R Core Team) within the RStudio environment.

When 10 or more studies were available, small-study effects were assessed using funnel plots and the Egger test, which were treated as indicators of such effects rather than sole evidence of publication bias [51,52]. To systematically investigate potential effect modifiers, subgroup analyses were conducted for intervention duration (≤8 wk vs ≥12 wk), control type (exercise vs nonexercise), intervention type, implementation mode (home or remote vs center-based), and synchronization mode (synchronous vs asynchronous). A subgroup was not analyzed if it contained fewer than 4 included studies. All subgroup findings are exploratory and do not imply causality. These subgroup analyses were not specified in the preregistered protocol. All outcomes reported align fully with the preregistered protocol. Sensitivity analysis was performed using the leave-one-out method to evaluate the robustness of the pooled effect estimates. When 10 or more studies were included, funnel plots and the Egger test were used to assess small-study effects; it should be noted that these methods are designed primarily to detect small-study effects rather than to serve as conclusive evidence of publication bias [53,54].

## Results

### Search Results and Study Selection

**Figure 1.**
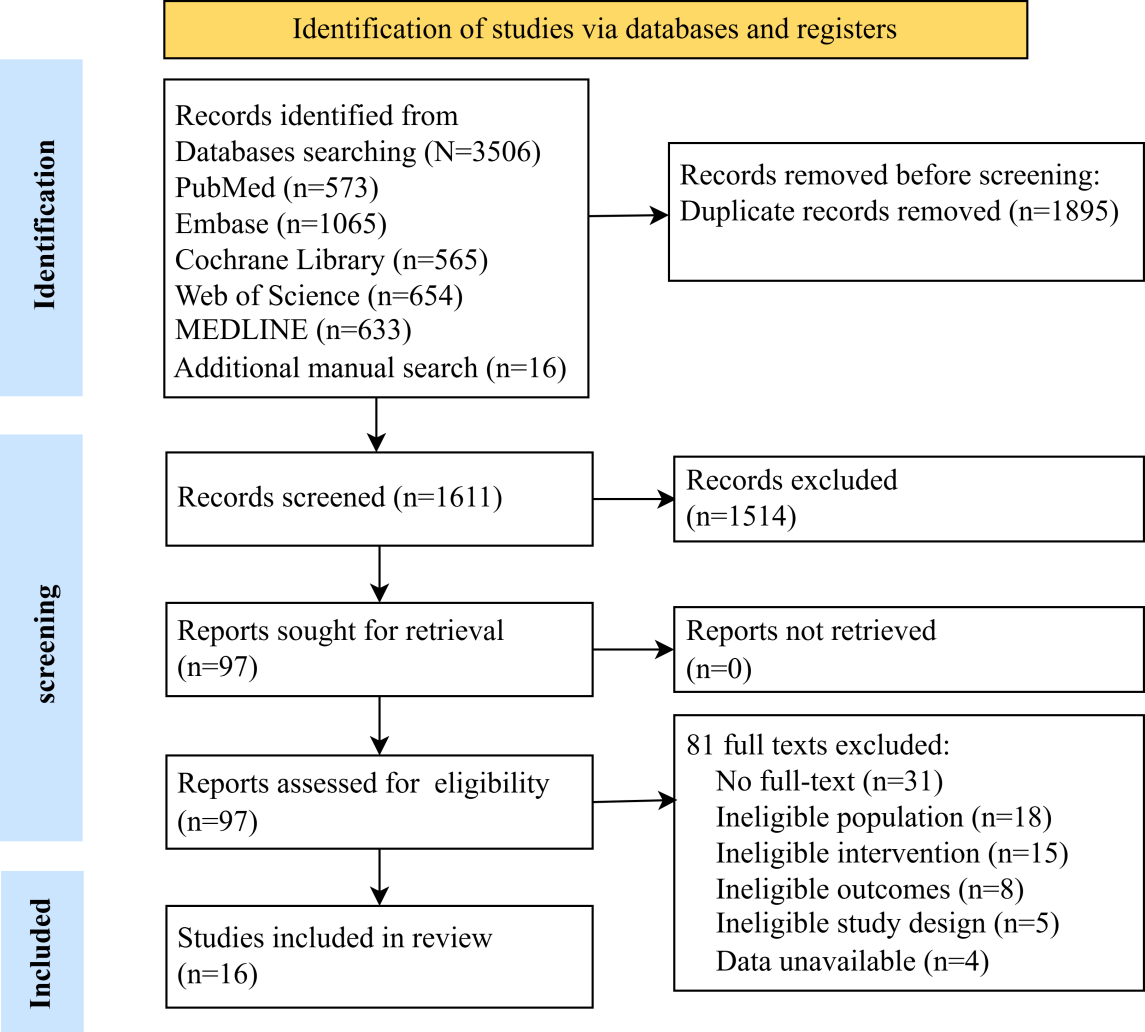
PRISMA (Preferred Reporting Items for Systematic Reviews and Meta-Analyses) flow diagram of the literature search and study selection.

In the initial systematic database search, a total of 3506 studies were identified. After the removal of 1895 duplicate entries, 1611 studies remained. Following title and abstract screening, 97 articles underwent full-text review. After full-text assessment, 81 studies were excluded for the following reasons: unavailable full text (n=31), lack of focus on sarcopenia or frailty (n=18), inconsistent intervention measures (n=15), differing outcome indicators (n=8), nonrandomized study design (n=5), and unobtainable or nonconvertible data (n=4). Ultimately, 16 studies met the inclusion criteria for the final analysis [55-70]. The detailed screening process is shown in Figure 1.

### Study Characteristics

This analysis included 16 studies [55-70] involving 1109 participants from 3 geographic regions, with sample sizes ranging from 15 to 201. The geographical distribution of the studies was as follows: 9 studies were conducted in China [55,57,59-62,64,65,70], 2 studies in Korea [63,69], and one each in the United States [56], England [67], and Thailand and Spain [58]; one additional study was a multicenter trial conducted across Italy, Germany, Austria, Spain, England, Belgium, Sweden, Japan, Korea, and Australia [66]. All were RCTs completed between 2012 and 2025. Four trials used a 3-arm design [55,56,63,65], one a 4-arm design [57], and the rest a 2-arm design [58-62,64,66-70]. Seven studies focused on frailty [56,58,60,61,63,66,70], 8 on sarcopenia [55,57,59,64,65,67-69], and 1 on both [62]. Most compared DHIs with control conditions such as usual care, health education, or nondigital exercise.

DHIs mainly included apps, artificial intelligence, virtual reality, exergames (such as those using Kinect and Nintendo Wii), health intervention platforms, wearable devices, and hybrid DHIs. These were implemented in diverse settings such as hospitals, community health centers, and senior day care centers. The duration of interventions across studies varied considerably, ranging from as short as 4 weeks to as long as 12 months. The intervention frequency was most often 2‐3 sessions per week, each lasting 20‐60 minutes. Outcome measures included muscle mass, muscle strength, physical performance, and quality of life. Detailed study and population characteristics are in Table 1.

**Table 1.** Characteristics of the included studies.^a^

Study (year)	Country	Study design	Disease	Diagnostic criteria	Population (sample size [n, male or female], ages, years [mean, SD])	Intervention (brief introduction)	Intervention format	Control	Intervention frequency, duration, length of follow-up	Outcomes (measures)	ITT^b^
Wei et al (2025) [55]	China	RCT^c^	Sarcopenia	AWGS^d^	EG1^e^: 27 (14/13) Age: 71.57 (7.24) EG2: 25 (13/12) Age: 72.56 (7.76) CG:^f^ 24 (14/10) Age: 70.77, (8.27)	EG 1: Home or remote-based individual exercise intervention with real-time AI^g^ supervision and guidance EG 2: Center-based group intervention with real-time instructor supervision and feedback	EG 1: AI EG 2: Videoconferencing platform	Exercise	Frequency: 3 times a week Duration: 3 months Follow-up: NA^h^	Muscle mass: ASMI^i^ Muscle strength: grip strength Physical performance: gait speed, TUGT^j^ QoL^k^: SF-36^l^	NA
Daniel et al (2012) [56]	United States	RCT	Prefrailty	Frailty phenotype	EG1: 8 (3/5) Age: 80, (3.37) EG2: 8 (3/5) Age: 78.13, (5.5) CG: 7 (3/4) Age: 72.6 (4.6)	EG: Center-based group with real-time supervision and guidance by study staff during sessions	Exergame (Nintendo Wii)	Exercise	Frequency: 3 times a week Duration: 15 weeks Follow-up: NA	Physical performance: 30CST^m^, TUGT, gait speed, 6MWT^n^	NA
Wang et al (2022) [57]	China	RCT	Sarcopenia	AWGS and EWGSOP^o^	EG1: 50 (8/42) Age: 70.16 (4.32) EG2: 50 (9/41) Age: 68.18 (3.93) EG3: 50 (10/40) Age: 69.72 (3.60) CG: 51 (7/44) Age: 69.88 (3.29)	EG1: Home or remote individual intervention with combined nutrition and exercise, no real-time feedback or supervision EG 2: Home or remote individual intervention with nutrition, no real-time feedback or supervision. EG 3: Home or remote individual intervention with exercise, no real-time feedback or supervision	Mobile app	Education	Frequency: 3 times a week or more Duration: 12 weeks Follow-up: NA	Muscle mass: ASMI, skeletal muscle mass Physical performance: gait speed, timing sitting and standing test, balance Body composition: BMI	NA
Mollà-Casanova et al (2023) [58]	Spain	RCT	Prefrailty and frailty	Frailty phenotype	EG: n=19 (2/17) Age: 71.68 (4.4) CG: 19 (3/16) Age: 72.26 (3.33)	EG: Center-based group exercise with real-time instructor supervision and feedback	Virtual running	Placebo	Frequency: 3 times a week Duration: 8 weeks Follow-up: 4 weeks	Physical performance: 2MWT^p^, FTSST^q^	NA
Ho et al (2024) [59]	China	RCT	Sarcopenia	AWGS	EG: 27 (3/24) Age: 70.26, (4.72) CG: 31 (6/25) Age: 74.26 (6.30)	EG: Home or remote individual intervention with nutritional education, no real-time supervision or guidance	Wearable activity tracker	Education	Frequency: daily Duration: 8 weeks Follow-up: NA	Muscle mass: SMI^r^ Muscle strength: grip strength Physical performance: FTSST Body composition: BMI	NA
Liao et al (2019) [60]	China	RCT	Prefrailty and frailty	Frailty phenotype	EG: 27 (8/19) Age: 79.6 (8.5) CG: 25 (8/17) Age: 84.1 (5.5)	EG: Center-based group exercise with real-time feedback, supervised by a physical therapist	Exergame	Exercise	Frequency: 3 times a week Duration: 12 weeks Follow-up: none	Muscle strength: grip strength Physical performance: 30CST, single leg stance, TUGT, gait speed	NA
Liu et al (2021) [61]	China	RCT	Frailty	Fried Frailty Index	EG: 22 (5/17) Age: 72.1 (3.7) CG: 18 (1/17) Age: 80.4 (6.83)	EG: Home or remote group exercise intervention with no real-time supervision or feedback	Wearable activity tracker	Exercise	Frequency: 3 times a week Duration: 12 weeks Follow-up: none	Physical performance: 30CST, TUGT, 2MWT	NA
Tuan et al (2024) [62]	China	RCT	Frailty and sarcopenia	AWGS, osteoporotic fractures index	EG: 30 (10/20) Age: 78.83 (7.71) CG: 30 (11/19) Age: 78.73 (6.82)	EG: Center-based group exercise with real-time supervision and feedback	Exergame	Standard care	Frequency: 2 times a week Duration: 12 weeks Follow-up: NA	Muscle mass: skeletal muscle mass, ASMI Muscle strength: grip strength Physical performance: gait speed QoL: SF-36	YES
Lee (2025) [63]	Korea	RCT	Prefrailty	Frailty phenotype	EG1: 33 (17/13) Age: 84.90 (2.85) EG2: 30 (15/15) Age: 85.18 (3.14) CG: 31 (16/14) Age: 84.81 (2.71)	EG 1: Home or remote group exercise with real-time supervision and feedback provided by physical therapists EG 2: Center-based group exercise with real-time supervision and feedback provided by physical therapists	Videoconferencing app	Education	Frequency: 2 times a week Duration: 8 weeks Follow-up: NA	Physical performance: FTSTS, 30CST, gait speed, TUGT, BBS^s^	NA
Zhang et al (2025) [64]	China	RCT	Sarcopenia	AWGS	EG: 24 (6/18) Age: 70.47 (6.05) CG: 27 (7/20) Age: 69.81 (5.76)	EG: Home or remote individual exercise, with no real-time supervision or feedback	Mobile app	Exercises	Frequency: 3 times a week Duration: 4 weeks Follow-up: NA	Muscle mass: ASMI, skeletal muscle mass Muscle strength: grip strength Physical performance: TUGT, 30CST, 6MWT, Balance	NA
He et al (2024) [65]	China	RCT	Sarcopenia	AWGS	EG1: 24 (NA) Age: 73.67 (4.77) EG2: 23 (NA) Age: 72.26 (4.43) CG: 23 (NA) Age: 70.91 (3.94)	EG 1: Home or remote-based individual exercise intervention with real-time AI supervision and guidance EG 2: Center-based group intervention with real-time instructor supervision and feedback	EG 1: AI EG 2: Videoconferencing app	Exercise	Frequency: 3 times a week Duration: 3 months Follow-up: NA	Muscle mass: ASMI Muscle strength: grip strength Physical performance: gait speed, TUGT QoL: SF-36	NA
Rainero et al (2021) [66]	Italy, Germany, Austria, Spain, England, Belgium, Sweden, Japan, Korea, Australia	RCT	Prefrailty	Frailty phenotype	EG: 101 (30/71) Age: 70.37 (6.15) CG: 100 (23/77) Age: 73.40 (6.57)	EG: Home or remote-based individual multicomponent intervention, including nutritional support, with no real-time supervision or feedback	Health intervention platform	Education	Frequency: NA Duration: 12 months Follow-up: NA	Muscle strength: grip strength Physical performance: TUGT, gait speed, balance Fear of falling: ABC^t^ QoL: WHO-QoL^u^	NA
Bailey et al (2024) [67]	England	RCT	Sarcopenia	EWGSOP, Clinical Frailty Scale version 2.0	EG: 23 (NA) Age: 75 (7) CG: 26 (NA) Age: 74 (6)	EG: Home or remote-based individual exercise with no real-time supervision or feedback	Hybrid digital health interventions	Usual care	Frequency: NA Duration: 6 months Follow-up: NA	Muscle strength: grip strength Physical performance: Balance, SPPB^v^ QoL: SarQol^w^ Body composition: BMI	NA
Yuenyongchaiwat et al (2026) [68]	Thailand	RCT	Sarcopenia	AWGS	EG: 23 (4/19) Age: 69.6 (4.6) CG: 23 (5/18) Age: 71.6 (6.3)	EG: Home or remote-based individual exercise with no real-time supervision or feedback	Virtual reality	Education	Frequency: 3 times a week Duration: 12 weeks Follow-up: NA	Muscle mass: SMI Muscle strength: grip strength Physical performance: gait speed	YES
An et al (2025) [69]	Korea	RCT	Sarcopenia	AWGS	EG: 15 (0/15) Age: 76.24 (8.7) CG: 15 (0/15) Age: 74.88 (9.1)	EG: Center-based individual exercise with therapist supervision and real-time feedback provided via mixed reality devices	Mixed reality	Exercise	Frequency: 5 times a week Duration: 4 weeks Follow-up: NA	QoL: SF-12^x^	NA
Liu et al (2025) [70]	China	RCT	Prefrailty and frailty	Frailty phenotype	EG: 15 (7/8) Age: 75.80 (6.99) CG: 15 (8/7) Age: 76.13 (8.16)	EG: Center-based exercise with no real-time supervision or feedback, individual or group not specified	Exergaming (Nintendo Switch sports games)	Exercise	Frequency: 2 times a week Duration: 12 weeks Follow-up: 1 month and 3 months	Muscle mass: ASMI Muscle strength: grip strength Physical performance: SPPB, TUGT	YES

a The ASMI and SMI refer to the same indicator (appendicular lean mass divided by height squared). To maintain consistency, the term ASMI is adopted for all subsequent analyses in this study; the 30SSRT and 30CST are identical in their testing protocol. To ensure consistency in reporting and analysis, the metric 30CST will be used uniformly throughout the subsequent analyses of this study.

b ITT: intention-to-treat.

c RCT: randomized controlled trial.

d AWGS: Asian Working Group for Sarcopenia.

e EG: experimental group.

f CG: control group.

g AI: artificial intelligence.

h NA: not applicable.

i ASMI: Appendicular Skeletal Muscle Mass Index.

j TUGT: Timed Up and Go Test.

k QoL: Quality of Life.

l SF-36: 36-item short form survey.

m 30CST: 30-second Chair Stand Test.

n 6MWT: 6-minute walk test.

o EWGSOP: European Working Group on Sarcopenia in Older People.

p 2MWT: 2-minute walk test.

q FTSST: Five Times Sit to Stand Test.

r SMI: Skeletal Muscle Index.

s BBS: Berg Balance Scale.

t ABC: activities-specific balance confidence scale.

u WHO-QoL: World Health Organization Quality of Life.

v SBBP: Short Physical Performance Battery.

w SarQol: Sarcopenia Quality of Life.

x SF-12: 12-Item short form health survey.

### Risk of Bias and Quality of Evidence

**Figure 2.**
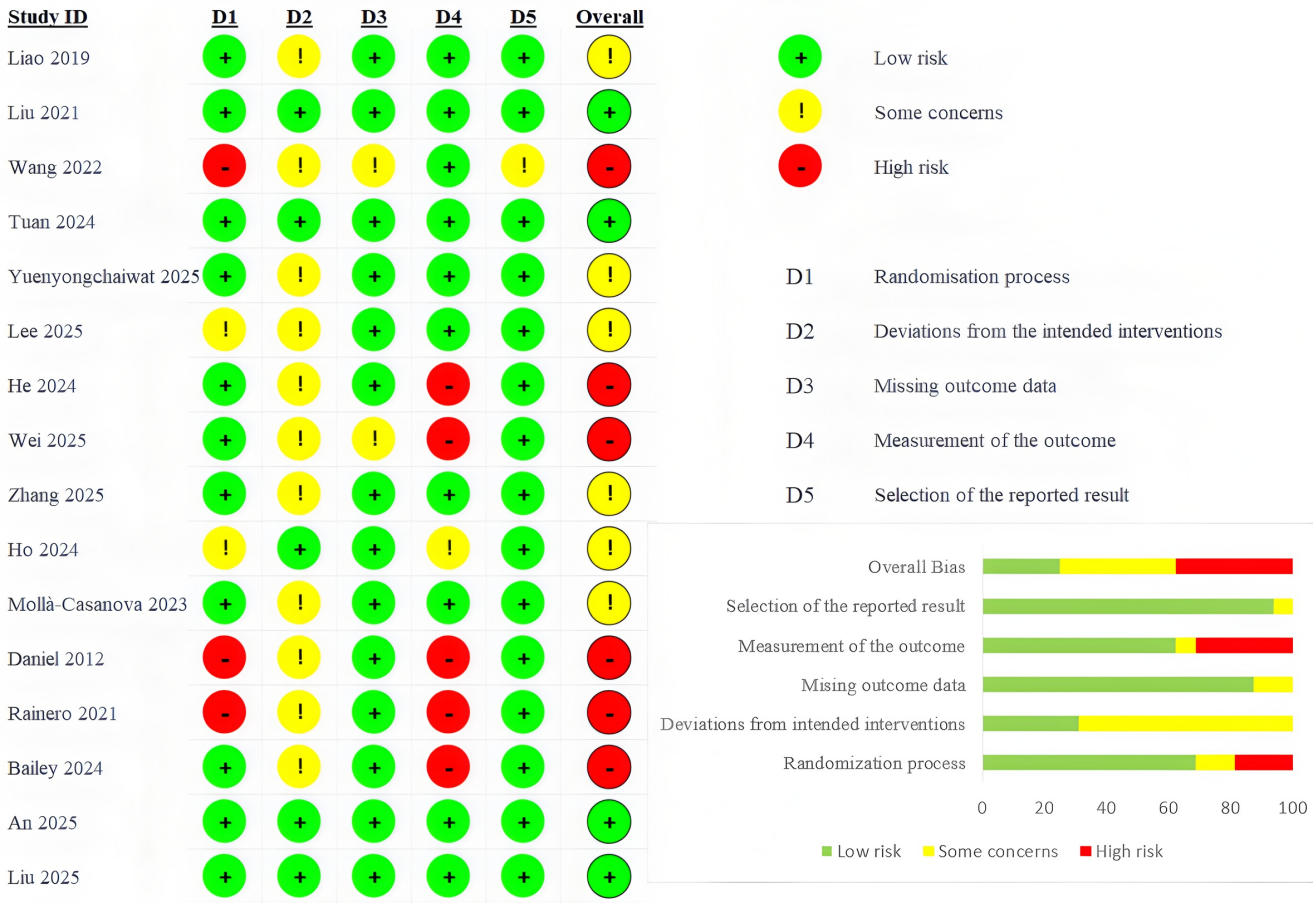
Risk of bias of included randomized controlled trials [55-70].

The results of the risk of bias assessment are shown in Figure 2. Six were rated as high risk of bias [55-57,65-67], 6 as some concerns [58-60,63,64,68], and 4 as low risk of bias [61,62,69,70]. In Domain 1 (randomization process), 1 study used sequential enrollment for allocation [66] and 2 studies did not provide detailed descriptions of the randomization implementation process, with unbalanced baseline data observed [56,57], resulting in a high risk of randomization bias. In Domain 2 (deviations from intended interventions), 11 studies did not report the use of ITT analysis or modified ITT analysis [55-58,60,63-68], thus indicating some concerns regarding bias. In Domain 3 (missing outcome data), one study exhibited a high attrition rate without reporting specific reasons for withdrawal [51], while another study indicated that participants’ dropout might be related to their health status [55]. Both of these scenarios have raised concerns regarding the risk of bias from missing outcome data. For the remaining studies included in the meta-analysis, the number of dropouts was generally balanced between groups, and the reasons for withdrawal were unrelated to the study outcomes. Therefore, missing data are unlikely to have distorted the true effects, indicating a low risk of bias due to missing outcome data. In Domain 4 (measurement of the outcome), a total of 6 studies were assessed to have some degree of risk of bias [55,56,59,65-67]. Among them, 5 studies were rated as being at high risk of bias because they did not report whether outcome assessors were blinded, and all their outcome measures incorporated subjective assessment items [55,56,65-67]. Another study was judged to have some concerns regarding risk of bias; although it also failed to report the implementation of blinding, all its outcome measures were objective assessments [59]. In Domain 5 (selection of the reported result), all but one study [57] was consistent with their prepublished protocols.

In GRADE assessments, overall certainty across all comparisons and outcomes is very low to low due to combinations of risk of bias, inconsistency, and imprecision (Multimedia Appendix 2).

### Effectiveness of DHIs on Outcomes

#### Effects of DHIs on Muscle Strength

Ten studies [55,59,60,62,64-68,70] assessed grip strength, one of which [70] included follow-up data. After splitting the sample size of shared control groups [55,65] in multiarm trials, 12 study arms were included in the analysis. Although the heterogeneity statistic *I*²=0%, a random-effects model was applied to provide a more conservative and generalizable estimate, given the variability in participant characteristics and intervention content across studies. Compared with controls, DHIs did not significantly improve grip strength (WMD 0.49, 95% CI –0.43 to 1.41, 95% PI –1.43 to 2.42; τ²=0.16; *P*=.50; Figure S1 in Multimedia Appendix 3). The 95% CI includes zero, indicating a statistically nonsignificant average effect. The 95% PI also spans zero, reflecting considerable uncertainty in potential future effects. During follow-up, no significant improvement in grip strength was observed with DHIs (WMD 0.49, 95% CI –0.43 to 1.41, 95% PI –1.43 to 2.42; *I*²=0%; τ²=0.17; *P*=.50; Figure S2 in Multimedia Appendix 3). Again, both the 95% CI and the 95% PI include zero, suggesting statistical nonsignificance and substantial variability in possible future outcomes. However, the reliability of this 95% PI should be interpreted cautiously due to the limited number of available studies. In view of the risks of methodological bias and imprecision, the overall quality of evidence for the effect of DHIs on muscle strength was rated as “low” (Multimedia Appendix 2).

#### Effects of DHIs on Muscle Mass

Three studies [57,62,64] evaluated skeletal muscle mass. By dividing the sample size of the shared control group in a multiarm trial [57], 5 study arms were ultimately included for analysis. Although the heterogeneity statistic being *I*²=0%, a random-effects model was used to provide a more conservative and generalizable effect estimate, given the variations in participant characteristics and intervention content across studies. In the analysis, the DHIs group showed a significant improvement in skeletal muscle mass compared to the control group (WMD 1.01, 95% CI 0.08-1.94, 95% PI –0.95 to 2.96; τ²=0; *P*=.47; Figure S3 in Multimedia Appendix 3). While the 95% CI confirms the statistical significance of the average effect, it should be noted that the 95% PI includes zero. This indicates that the beneficial effect may not be replicable in future studies or different populations. The interpretation of this PI, however, is limited by the small number of included studies. Overall, the quality of evidence supporting the effect of DHIs on skeletal muscle mass is rated as “very low” (Multimedia Appendix 2), due to risks of bias, heterogeneity in results, and data imprecision.

Eight studies [55,57,59,62,64,65,68,70] evaluated ASMI, with one study [70] providing longitudinal data. After dividing shared control group samples in multiarm trials [55,57,65], 12 study arms were included in the analysis. Pooled results showed that DHIs did not significantly improve ASMI compared with the control group (WMD 0.18, 95% CI −0.04 to 0.40, 95% PI −0.46 to 0.82; τ²=0.06; *P*=.03; Figure S4 in Multimedia Appendix 3). Heterogeneity was moderate (*I*²=48.2%). The 95% CI included zero, indicating a nonsignificant average effect; similarly, the 95% PI spanned zero, suggesting that DHIs may show no clear benefit or could even be unfavorable for ASMI in future studies or different populations. The limited number of included studies may affect the reliability of this 95% PI. During follow-up, DHIs also showed no significant improvement in ASMI (WMD 0.18, 95% CI −0.04 to 0.4, 95% PI −0.46 to 0.82; *I*²=48.4%; τ²=0.06; *P*=.03; Figure S5 in Multimedia Appendix 3). The 95% CI crosses zero, indicating that the average effect was not statistically significant. The 95% PI also spans zero, reflecting high uncertainty in future effects. Similarly, the reliability of this PI should be interpreted with caution, particularly given the limited number of follow-up studies. Due to risk of bias, inconsistency, and imprecision, the quality of evidence for the effect of DHIs on ASMI was rated as “very low” (Multimedia Appendix 2).

Subgroup analyses revealed that the improvement in ASMI was more pronounced in the ≧12 weeks subgroup (WMD 0.28, 95% CI 0.06-0.50, 95% PI −0.30 to 0.83; *I*²=32.6%; τ²=0.03; *P*=.15) compared with the ≤8 weeks subgroup (SMD −0.2, 95% CI −1.65 to 1.26; *I*²=0; τ²=0; *P*=.48; Multimedia Appendix 4). A test for subgroup differences confirmed a statistically significant distinction between the 2 subgroups (*χ*²_₁_=9.93; *P*=0.0016). It should be noted that this subgroup analysis was conducted post hoc and lacks the randomization of the original RCTs, thereby precluding strict control for confounding factors. Consequently, the observed difference in ASMI improvement is observational in nature and does not constitute causal evidence that intervention duration drives such a difference.

#### Effects of DHIs on Physical Performance

##### Gait Speed

Eight studies [55,57,60,62,63,65,66,68] measured gait speed. By splitting the sample size of shared control groups in multiarm trials [57,63,65], 13 study arms were ultimately included for analysis. The pooled results indicated that, compared with the control group, DHIs significantly improved gait speed (WMD 0.09, 95% CI 0.03-0.15, 95% PI −0.1 to 0.26; τ²=0; *P*=.07; Figure S6 in Multimedia Appendix 3), with moderate heterogeneity across studies (*I*²=39.1%). The 95% CI confirmed the statistical significance of the average effect; however, the 95% PI crossed zero, indicating that DHIs might not improve gait speed in future studies or different populations. The reliability of this 95% PI may be limited by the small number of included studies. Due to risks of bias and imprecision, the quality of evidence for the effect of DHIs on gait speed was rated as “low” Multimedia Appendix 2). The impact of DHIs on gait speed appeared modest.

##### 2MWT

Two studies [58,61] reported data on 2MWT, with one study [58] providing follow-up information. Due to inconsistent measurement units across the 2 studies, SMD was used as the effect measure for meta-analysis. Results showed that compared with the control group, DHIs did not significantly improve the 2MWT (SMD 0.29, 95% CI −0.63 to 1.21; τ²=0; *P*=.75; Figure S7 in Multimedia Appendix 3), with no observed heterogeneity between studies (*I*²=0%). The 95% CI includes zero, indicating that the average intervention effect was not statistically significant. At follow-up, DHIs demonstrated a statistically significant positive effect on the 2MWT (SMD 0.37, 95% CI 0.24-0.50; *I*²=0%; τ²=0; *P*=.96; Figure S8 in Multimedia Appendix 3), representing a small to moderate effect size. However, all of the above analyses were based on only 2 studies [58,61], resulting in a limited evidence base, which constrains the reliability of the findings and precludes subgroup analyses and GRADE assessment.

##### The 6MWT

Two studies [56,64] reported data on 6MWT. Given the inconsistency in measurement units across the 2 studies, SMD was used as the effect measure to enable meta-analysis. The results indicated that, compared with the control group, DHIs did not significantly improve 6MWT (SMD 0.55, 95% CI −8.30 to 9.40; τ²=0.78; *P*=.03; Figure S9 in Multimedia Appendix 3), with high heterogeneity observed between studies (*I*²=78.4%). The 95% CI includes zero, suggesting that the average intervention effect did not reach statistical significance. Due to the lack of a significant intervention effect on the 6MWT outcome and the limited number of studies, no GRADE assessment, subgroup analysis, or sensitivity analysis was conducted.

##### The TUGT

Nine studies [55,56,60,61,63-66,70] assessed TUGT, with one study [70] providing follow-up data. After splitting the sample sizes of shared control groups in multiarm trials [55,63,65], 12 study arms were ultimately included in the analysis. The pooled results indicated that, compared with the control group, DHIs significantly improved TUGT (WMD –0.52, 95% CI –1.02 to –0.03, 95% PI –1.93 to 0.85; τ²=0.35; *P*=.03; Figure S10 in Multimedia Appendix 3). Moderate heterogeneity was observed across studies (*I*²=47.5%). The 95% CI confirms the statistical significance of the average effect; however, the wide 95% PI, which crosses the null line, suggests notable variability in intervention effects across future studies or populations, ranging from beneficial to null or even slightly unfavorable. In long-term follow-up, DHIs continued to show a significant improvement in TUGT (WMD −0.52, 95% CI −1.02 to –0.03, 95% PI −1.93 to 0.86; *I*²=47.6%; τ²=0.35; *P*=.03; Figure S11 in Multimedia Appendix 3). The 95% CI still supports the statistical significance of the average effect, while the 95% PI again crosses zero, reflecting persistent uncertainty in long-term outcomes. Given the limited number of included studies, the reliability of this 95% PI should be interpreted with caution. According to the GRADE evidence assessment, the quality of evidence supporting the effect of DHIs on TUGT improvement was rated as “low,” primarily due to risk of bias in some studies and imprecision in results (Multimedia Appendix 2).

##### The FTSST

Three studies [58,59,63] reported FTSST, with one study [58] providing follow-up data. After splitting the shared control group of a multiarm trial [63], 4 study arms were included in the meta-analysis. The pooled results indicated that, compared with the control group, DHIs did not improve FTSST (WMD −1.63, 95% CI −4.26 to 0.99, 95% PI −8.04 to 4.71; *I*²=85.8%; τ²=2.37; *P*<.001; Figure S12 in Multimedia Appendix 3), with substantial heterogeneity across studies. The 95% CI included zero, indicating that the mean intervention effect was not statistically significant. The 95% PI also crossed zero, suggesting that DHIs may remain ineffective or potentially unfavorable in future studies or different populations. The reliability of this 95% PI may be limited due to the small number of included studies. During follow-up, the effect direction was largely consistent with the postintervention results (WMD −1.80, 95% CI −4.37 to 0.76, 95% PI −8.19 to 4.45; *I*²=85.4%; τ²=2.28; *P*<.001; Figure S13 in Multimedia Appendix 3). The 95% CI still included the null value, and the 95% PI again crossed the null line, further supporting the lack of stability and statistical significance of the effect over the longer term. Similarly, given the limited number of studies, the reliability of this prediction interval should be interpreted with caution, as sparse data may reduce the accuracy of estimates of the distribution of future effects. The values of *I*² and τ² confirmed substantial variability in true effect sizes across studies, beyond what would be expected from random sampling error alone. Sensitivity analysis showed that removing any single study did not meaningfully alter the overall results, with no statistically significant changes observed (Figure S1 in Multimedia Appendix 5), indicating robustness of the primary meta-analysis findings. Given the nonsignificant effect of the intervention on FTSST and the limited number of studies, no GRADE assessment or subgroup analysis was performed for this outcome.

##### The 30CST

Five studies [56,60,61,63,64] measured the 30CST. After splitting the shared control group sample size in a multiarm trial [63], 6 study arms were included in the analysis. The pooled analysis showed that DHIs significantly improved 30CST compared with control (WMD 2.19, 95% CI 0.89-3.48, 95% PI –1.59 to 5.66; τ^2^=0.09; *P*=.15; Figure S14 in Multimedia Appendix 3), despite moderate heterogeneity (*I*²=38.1%). The 95% PI crossed zero, indicating that DHIs may not improve 30CST in future studies or different populations. The reliability of this 95% PI may be limited by the small number of included studies. Due to risk of bias, inconsistency, and imprecision, the quality of evidence for the effect of DHIs on the 30CST was rated as “very low” (Multimedia Appendix 2).

Subgroup results indicated that the improvement in the 30CST was greater in the ≤8 weeks subgroup (WMD 2.28, 95% CI 0.02-4.54, 95% PI −4.70 to 7.87; *I*²=40.1%; τ²=0.03; *P*=.19; Multimedia Appendix 4) than in the ≥12 weeks subgroup (WMD 2.26, 95% CI −5.02 to 9.55, 95% PI −12.30 to 16.21; *I*²=55.8%; τ²=4.27; *P*=.10; Multimedia Appendix 4). However, the between-subgroup difference was not statistically significant (*χ*²_₁_=0; *P*=.99). Regarding intervention type, application-based interventions showed a significant effect (WMD 2.28, 95% CI 0.02-4.54, 95% PI −4.48 to 7.93; *I*²=40.1%; τ²=0.03; *P*=.19; Multimedia Appendix 4). However, no significant differences in effect size were observed between different digital formats (*χ*²_₂_=0.14; *P*=.93). In summary, although varying trends in effect sizes were noted across the subgroups analyzed, no statistically significant between-subgroup differences were found in this analysis.

##### Balance

Six studies assessed balance [57,60,63,64,66,67]. Following adjustment for shared control groups in multiarm trials [57,63], 9 study arms were included in the analysis. The pooled results demonstrated a moderate improvement in balance with DHIs compared to controls (SMD 0.61, 95% CI 0-1.21, 95% PI –0.94 to 2.13; *I*²=82.3%; τ²=0.49; *P*<.001; Figure S15 in Multimedia Appendix 3). The lower limit of the 95% CI being zero indicates borderline statistical significance. Considerable heterogeneity was observed across studies. The 95% PI included zero, suggesting that the effect may vary substantially across different populations or future studies, and could potentially be null. The reliability of this prediction interval is limited by the small number of studies. Sensitivity analysis showed that the statistical significance of the intervention effect was lost upon removal of any single study, indicating that the findings lack robustness (Figure S2 in Multimedia Appendix 5). Due to risk of bias, inconsistency, and imprecision, the evidence regarding the effect of DHIs on balance was rated as “very low” (Multimedia Appendix 2).

To explore potential moderating factors affecting the improvement of balance function through DHIs, exploratory subgroup analyses were conducted based on intervention duration, comparator type, digital intervention type, delivery mode, and setting. Subgroup results indicated that only the exergaming group showed significant improvement in balance function (SMD 0.83, 95% CI 0.26-1.40; Multimedia Appendix 4); tests for subgroup differences confirmed statistically significant variation across subgroups (*χ*²_₃_=9.89; *P*=0.0195). Because subgroup analyses are observational in nature, even when derived from RCTs, they cannot establish causality but may suggest potential moderators. These findings should therefore be interpreted with caution.

##### The SPPB

Two studies [67,70] reported data on SPPB, with one study [70] providing follow-up information. Results showed that compared with the control group, DHIs did not significantly improve the SPPB (WMD −0.02, 95% CI −0.38 to 0.34, 95% PI −0.83 to 0.75; τ²=0; *P*=.90; Figure S16 in Multimedia Appendix 3), with no observed heterogeneity between studies (*I*²=0%). The 95% CI includes zero, indicating that the average intervention effect was not statistically significant. During follow-up, the effect direction was largely consistent with the postintervention results (WMD −0.03, 95% CI −0.65 to 0.58, 95% PI −1.49 to 1.35; *I*²=0%; τ²=0.0001; *P*=.84; Figure S17 in Multimedia Appendix 3). The 95% CI still included the null value, and the 95% PI also crossed zero, indicating no significant long-term effect. However, all of the above analyses were based on only 2 studies, resulting in a limited evidence base that constrains the reliability of the findings and precludes subgroup analyses and GRADE assessment.

### Effects of DHIs on Quality of Life

Six studies [55,62,65-67,69] assessed quality of life. Following the splitting of shared control group sample sizes from multiarm trials [55,65], 8 study arms were included in the analysis. Pooled results indicated a small but significant improvement in quality of life with DHIs compared to the control group (SMD 0.16, 95% CI 0.05-0.27, 95% PI 0.04-0.28; τ²=0; *P*=.97; Figure S18 in Multimedia Appendix 3). The 95% CI did not cross zero, confirming statistical significance. No heterogeneity was observed across studies (*I*²=0%). Similarly, the 95% PI did not cross zero, suggesting that the direction of the beneficial effect is likely to remain consistent in future studies or different populations. However, caution is warranted as the reliability of this 95% PI may be limited by the small number of included studies. Due to risk of bias and imprecision, the quality of evidence regarding the effect of DHIs on QoL was rated as “low” (Multimedia Appendix 2).

### Effects of DHIs on BMI

Three studies [57,59,67] evaluated BMI. By dividing the sample size of the shared control group in a multiarm trial [57], 5 study arms were ultimately included for analysis. The pooled analysis showed that DHIs did not significantly improve BMI compared with control (WMD 0.09, 95% CI –0.36 to 0.53, 95% PI –0.85 to 1.04; τ^2^=0.07; *P*=.16; Figure S19 in Multimedia Appendix 3), despite moderate heterogeneity (*I*²=38.6%). The 95% CI included zero, indicating that the mean intervention effect was not statistically significant. The 95% PI also included the null value, indicating that DHIs could be ineffective or even harmful in future research or other populations. Owing to the small number of included studies, the robustness of this 95% PI should be viewed as restricted. Given the nonsignificant effect of the intervention on BMI and the limited number of studies, no GRADE assessment or subgroup analysis was performed for this outcome.

### Small-Study Effects Assessment

We conducted small-study effect analyses using funnel plots combined with Egger regression test for outcome measures with at least 10 study arms included. Funnel plots (Figures S1-S4 in Multimedia Appendix 6) were constructed to visually explore potential asymmetry. Visual inspection revealed no marked asymmetry in the funnel plots for any outcome measure. Egger test results indicated no significant small-study effect for any outcome: grip strength (*P*=.92), ASMI (*P*=.54), gait speed (*P*=.59), or TUGT (*P*=.47). It should be noted that observed funnel plot asymmetry may not only reflect publication bias but could also arise from between-study heterogeneity, random error, or methodological differences. Therefore, the *P* values from Egger test in this study serve only as an indicator of potential small-study effects and should not be interpreted as definitive evidence of publication bias. This is especially relevant given the limited number of studies included, as these biases may remain undetected due to insufficient statistical power.

## Discussion

### Principal Findings

#### Overview

This study evaluated the effectiveness of DHIs in improving muscle function (muscle strength and muscle mass), physical function, and quality of life among patients with frailty and sarcopenia. Sixteen RCTs involving 1109 participants were included. The results indicated that DHIs significantly improved skeletal muscle mass, gait speed, TUGT, and 30CST. Additionally, a moderate benefit was seen in balance ability, alongside a slight but statistically significant enhancement in quality of life. In contrast, DHIs did not significantly improve grip strength, ASMI, FTSST, SPPB, and BMI. Subgroup analyses revealed that the effect on ASMI might be influenced by intervention duration, and the effect on balance ability could vary by intervention type. The reliability of these findings may be tempered by clinical heterogeneity across studies, including variations in intervention protocols and participant characteristics.

#### Muscle Function

Multiple diagnostic guidelines for frailty and sarcopenia consistently identify muscle strength, muscle mass, and physical function as core influencing factors of these conditions [5,38,71]. Muscle function primarily includes muscle strength and muscle mass, with grip strength serving as a straightforward and effective metric for assessing muscle strength [72]. Grip strength is also a critical component of the frailty and sarcopenia phenotype [73]. The comprehensive analysis of this study reveals that no significant improvement in grip strength was observed following DHIs, which contradicts previous research findings [29]. The 95% PI for muscle strength ranged from −1.43 to 2.42, indicating that in real-world clinical intervention settings, the efficacy of DHIs may vary significantly due to factors such as intervention protocols and participant characteristics, and in some instances, may exhibit no discernible effect. The lack of improvement in grip strength following DHIs may result from multiple factors. First, the training dose (intensity, frequency, and duration) in current DHIs is likely suboptimal for inducing strength gains in hand and forearm muscles [74]. Optimizing dose-response parameters, including volume, intensity, and progression, is crucial for the efficacy of future interventions. Furthermore, DHIs exhibit significant heterogeneity and frequently lack standardization, particularly in terms of incorporating targeted hand exercises. Furthermore, the majority of DHIs did not incorporate combined nutritional support, such as protein supplementation, which may constrain the anabolic response of muscles. Consequently, future research should consider the integration of structured exercise regimens with nutritional co-interventions.

For muscle mass outcomes, the meta-analysis demonstrated a significant beneficial effect of DHIs, although the effect on ASMI did not reach statistical significance. This finding is consistent with previous meta-analyses reporting improvements in total skeletal muscle mass but differs from conclusions regarding ASMI [29]. For skeletal muscle mass, the 95% CI did not intersect the null line, indicating a consistent average benefit of DHIs. However, the 95% PI did intersect the null, suggesting uncertainty in clinical application and potential variability in benefits across different studies or settings, including the possibility of no benefit. Similarly, the 95% PI for the ASMI intersected the null, indicating potential inconsistency in real-world effects. Subgroup analysis suggested a potential differential association between intervention duration and ASMI, with interventions lasting 12 weeks or more showing a statistically significant improvement in ASMI. Nonetheless, the 95% PI in this subgroup still intersected the null, underscoring substantial variability in long-term effects and indicating that the benefits of DHIs may vary considerably across populations or contexts. This variability may also be attributed to the small sample size of the subgroup and unmeasured confounding factors. It is important to note that these subgroup comparisons are observational in nature and deviate from the randomized design of the original studies, with the limited sample size further constraining generalizability. Considering these limitations, the observed differences are inadequate to guide clinical decisions concerning the optimal duration of interventions for enhancing ASMI. Future large-scale, high-quality RCTs specifically designed to compare intervention durations are necessary to substantiate these preliminary findings.

In conclusion, the observed variation in the effects of DHIs on these 2 metrics may be attributable to 3 principal considerations. First, there exists an intrinsic difference in the nature of the metrics. Total muscle mass represents an absolute value that is readily influenced by fluctuations in body weight. ASMI is a height-standardized relative measure that more specifically reflects limb muscle status in relation to physical function [75]. Second, many existing DHI programs lack targeted stimulation of limb muscle groups and exhibit considerable interprotocol heterogeneity, which may partially explain their limited effectiveness in improving ASMI. Finally, the current body of evidence has limitations, including small sample sizes, insufficient statistical power, and potential inconsistencies in muscle mass measurement methodologies across studies. These findings highlight the potential value of considering both absolute muscle mass and standardized metrics such as ASMI in clinical assessments to more thoroughly evaluate the impact of DHIs. Future research would benefit from prioritizing large-scale, methodologically rigorous clinical trials that integrate targeted limb training within intervention protocols and standardize the reporting of both absolute and standardized muscle mass measures. Such an approach is expected to help clarify the specific effects of DHIs on different dimensions of muscle health, which could support the development of a more reliable foundation for their targeted clinical application.

#### Physical Performance

Physical performance is a core feature of frailty and sarcopenia [76] and a key driver of their associations with adverse health outcomes [77]. This study evaluated various physical performance metrics, such as gait speed, 6MWT, 2MWT, TUGT, 30CST, FTSST, SPPB, and balance. The results of the meta-analysis indicated that DHIs led to significant enhancements in gait speed, TUGT, 30CST, and balance among patients with frailty and sarcopenia. The observed improvement in gait speed aligns with findings from previous research [28,30]. The 95% PI for gait speed encompassed the null value, indicating that, despite evidence of a positive intervention effect, substantial uncertainty persists regarding the real-world effectiveness of DHIs. This uncertainty is attributed to factors such as population heterogeneity, diverse clinical settings, and variations in implementation. Additionally, the observed improvement in gait speed (0.09 m/s) did not meet the MCID threshold of 0.10 m/s [78]. This suggests that, although statistically significant, the change may not confer a clinically meaningful functional benefit. Therefore, future large-scale RCTs are necessary to ascertain whether DHIs can consistently achieve the MCID for gait speed and to elucidate their efficacy across various clinical contexts.

In addition to gait speed, the improvement in TUGT was also statistically significant. Notably, the 95% PI for TUGT similarly includes the null value, suggesting potential substantial heterogeneity in its real-world clinical effect. Furthermore, the magnitude of improvement in TUGT (−0.52 s) did not reach the established MCID threshold (2.1 s) [79], indicating that the observed change may not yet translate into clinically meaningful functional benefits. Future large-scale RCTs are needed to determine whether DHIs can consistently achieve the MCID for both gait speed and TUGT, and to clarify their robustness across diverse clinical settings.

For the 30CST, DHIs resulted in a statistically significant improvement of 2.19 repetitions, exceeding the prespecified MCID of 2 repetitions [80]. This suggests a potential for clinically meaningful functional benefit. However, the 95% PI included the null value, indicating substantial uncertainty in the effect’s consistency across different populations or clinical settings. Subgroup analyses by intervention duration, delivery mode, and intervention type suggested numerical differences in effect sizes, though none of these comparisons were statistically significant, which does not support these factors as clear determinants of the treatment effect on the 30CST. In clinical interpretation, it is imperative to prioritize the potential clinical benefits of the average effect, while also recognizing its inherent variability. It is inadvisable to make definitive efficacy predictions solely based on current subgroup characteristics. To effectively translate this potential effect into a stable and generalizable clinical practice, future large-scale, stratified clinical trials are necessary. These trials should be designed to ascertain whether the intervention can consistently meet and surpass the clinically significant threshold across diverse populations, thereby offering conclusive evidence for precise clinical recommendations.

DHIs exhibited a notable enhancement in balance. Nonetheless, the 95% PI for balance included the null value, and no MCID for balance has been established in populations with frailty and sarcopenia. Therefore, whether this improvement translates into clinically meaningful benefit remains unclear. Future studies should focus on establishing an MCID for balance in these populations, followed by large-scale trials to determine whether DHIs can consistently achieve clinically significant improvements. Subgroup analysis indicated that exergaming is an effective DHI modality for improving balance in patients with frailty and sarcopenia, which aligns with findings from systematic reviews in healthy older adults [81]. Despite the fact that only one study within the analysis used exergaming, it underscored the potential of highly specific, interactive applications to improve balance. By merging exercise with gameplay, these applications engage patients in physical activity in an enjoyable manner, potentially enhancing mental well-being, exercise enjoyment, and ultimately balance [82]. This interactive approach is likely to increase patient motivation and acceptance, thereby improving adherence and sustained engagement. These factors may contribute to its enhanced effectiveness, though this interpretation should be approached with caution.

#### Quality of Life

DHIs demonstrated a statistically significant positive impact on the quality of life among patients with frailty and sarcopenia. The 95% CI for quality of life did not intersect the null value, thereby confirming the statistical significance of this positive effect. Furthermore, the 95% PI remained entirely within the positive range and excluded zero, indicating a consistent beneficial effect across various clinical settings or populations. Nonetheless, the effect size was small, necessitating cautious interpretation of its clinical relevance, which should be considered in light of individual circumstances and specific contexts. Given the multidimensional nature of quality of life, future research should aim to develop and evaluate integrated digital health programs that incorporate psychosocial and multidisciplinary components, with the goal of achieving more clinically meaningful improvements beyond the observed small effect [83].

#### BMI Changes

This study demonstrated that DHIs did not significantly improve BMI, primarily due to the wide range of baseline BMI values among the included populations (BMI 22.26‐31.70 kg/m²), which to some extent attenuated the overall effect size of the intervention. Existing evidence indicates that multicomponent exercise interventions incorporating aerobic, resistance, and functional training are more effective [84,85], suggesting that standalone DHIs are limited in weight management owing to the lack of a structured design. Meanwhile, BMI cannot distinguish between muscle and fat mass, thus limiting its evaluative value in older adults with sarcopenia [84]. Therefore, future studies should rigorously control baseline characteristics to reduce heterogeneity, develop structured multimodal intervention programs, and adopt a comprehensive assessment combining BMI with multiple indicators such as muscle mass and body composition.

#### Follow-Up

The TUGT was the only intervention that demonstrated sustained improvement during the follow-up period. Given the limited number of studies assessing long-term effects, additional research is necessary to elucidate its enduring efficacy [86].

#### Heterogeneity Across Studies

Exploratory subgroup analyses were undertaken for outcomes exhibiting moderate to high heterogeneity, which was predefined as involving more than 5 studies. The pooled analyses indicated moderate heterogeneity for the TUGT, 30CST, gait speed, and ASMI. In contrast, greater heterogeneity was observed for the 6MWT, FTSST, and balance assessments. Due to an insufficient number of studies, subgroup analyses were not conducted for the 6MWT, FTSST, SPPB, and BMI. The observed heterogeneity is likely attributable to variations in intervention protocols, such as frequency, duration, components, and specific methods, as well as differences in study population characteristics, including age and sample size.

### Limitations

This study has several limitations. First, although the search strategy did not restrict language, only English-language publications were ultimately included, which may introduce language bias and reduce the applicability of the findings to non–English-speaking contexts. Second, only RCTs were selected to ensure methodological rigor, which may have excluded non-RCT evidence relevant to the real-world implementation and long-term feasibility of digital health tools. Third, most included studies were conducted in China, limiting the applicability of the results to other health care systems and cultural settings. Finally, substantial heterogeneity in outcome measures and control conditions across studies complicates result synthesis and limits the clarity of conclusions regarding DHI effectiveness. Future research should address these limitations through high-quality, multicenter, and cross-cultural clinical trials to verify the effectiveness of DHIs and clarify their optimal implementation models.

### Implications for Clinical Practice

While the overall certainty of evidence remains limited, DHIs have the potential to serve as a scalable and patient-centered complement to traditional rehabilitation methods for individuals experiencing frailty and sarcopenia. To facilitate the integration of DHIs into existing health care systems, clinicians should consider providing structured support across 3 critical domains: accessibility, user engagement, and technical literacy. Regarding accessibility, DHIs should be thoughtfully integrated into clinical pathways, with particular attention given to patients facing functional limitations, mobility challenges, or residing in remote locations, thereby helping to mitigate geographical and physical barriers. Furthermore, the establishment of regular follow-up procedures may aid in addressing ongoing usability concerns and in evaluating sustained outcomes. To improve patient engagement, it is advisable to recommend high-interactivity tools, such as exergames, that align with individual patient interests. For older adults, DHIs should prioritize senior-friendly features, including simplified interfaces and voice assistants, to minimize barriers and enhance adherence. In terms of technical literacy, clinical teams should develop proficiency in the operation of these tools and in providing patient guidance through comprehensive training programs. Initial support in digital health literacy should be extended to patients, while family members or caregivers should be encouraged to assist individuals with limited technical adaptability, thereby ensuring the sustainability of the intervention.

### Conclusion

This study is the first systematic review to examine the impact of DHIs on muscle function (including muscle mass and muscle strength), physical performance, and quality of life among older adults experiencing frailty and sarcopenia. The results indicate that DHIs generally surpass control conditions, resulting in improvements in muscle mass and specific physical performance measures, such as gait speed, TUGT, 30CST, and balance. Additionally, DHIs have a positive impact on quality of life. Nevertheless, these results warrant cautious interpretation. Except for quality of life, all other outcomes exhibited wide PIs encompassing the null value, indicating significant heterogeneity across studies. This variability suggests that statistically significant improvements may not be consistently observed across different populations or clinical settings. The conclusions of this review must be viewed considering its methodological limitations. The wide prediction intervals for muscle mass, gait speed, TUGT, 30CST, and balance, especially the high heterogeneity and instability in balance outcomes, limit the findings’ generalizability. Some included RCTs had methodological issues, such as blinding challenges and unclear allocation concealment, potentially leading to an overestimation of DHIs’ effects. According to the GRADE framework, the evidence quality for outcomes such as muscle mass, gait speed, TUGT, 30CST, balance, and quality of life is rated very low to low due to concerns about bias, inconsistency, and imprecision, reducing confidence in the intervention’s benefits.

Nevertheless, DHIs continue to be regarded as a viable strategy for improving the accessibility and cost-effectiveness of managing frailty and sarcopenia. These interventions are particularly valuable for addressing treatment gaps among populations that face barriers to adequate health care services, such as limited regional resources, time and transportation constraints, or physical functional limitations. Future research endeavors should prioritize the development of a standardized core outcome set for DHIs and the execution of robust, multicenter, large-scale, high-quality RCTs that include nutritional support components. These trials should also assess long-term health benefits and cost-effectiveness. Generating such high-quality evidence will facilitate the standardized application and effective integration of DHIs into the clinical management of frailty and sarcopenia, thereby establishing a foundation for their evolution into scalable and sustainable public health solutions to meet the global prevention and control requirements of these conditions.

## Supplementary material

10.2196/88374 Multimedia Appendix 1 The search strategies.

10.2196/88374 Multimedia Appendix 2 Grading of Recommendations, Assessment, Development, and Evaluation evidence profile.

10.2196/88374 Multimedia Appendix 3 Forest plot.

10.2196/88374 Multimedia Appendix 4 Subgroup analysis.

10.2196/88374 Multimedia Appendix 5 Sensitivity analysis.

10.2196/88374 Multimedia Appendix 6 Funnel plots.

10.2196/88374 Checklist 1 PRISMA checklist.

## Abbreviations

2MWT: 2-minute walk test
30CST: 30-second Chair Stand Test
6MWT: 6-minute walk test
ASMI: Appendicular Skeletal Muscle Mass Index
AWGS: Asian Working Group for Sarcopenia
DHI: digital health intervention
EWGSOP: European Working Group on Sarcopenia in Older People
FTSST: Five Times Sit to Stand Test
GRADE: Grading of Recommendations, Assessment, Development, and Evaluation
HKSJ: Hartung-Knapp-Sidik-Jonkman
ITT: intention-to-treat
MCID: Minimal Clinically Important Difference
MeSH: Medical Subject Headings
PI: prediction interval
PICOS: Population, Intervention, Comparison, Outcomes, and Study Design
PRISMA: Preferred Reporting Items for Systematic Reviews and Meta-Analyses
PRISMA-S: Preferred Reporting Items for Systematic Reviews and Meta-Analyses search extension
RCT: randomized controlled trial
RoB 2.0: Cochrane Risk of Bias tool
SMD: standardized mean difference
SPPB: Short Physical Performance Battery
TUGT: Timed Up and Go Test
WMD: weighted mean difference
