# Supplementary material for: Effectiveness of Digital Health Interventions in Older Adults With Frailty and Sarcopenia: Systematic Review and Meta‐Analysis of Randomized Controlled Trials
Source: J Med Internet Res. 2026 May 11;28:e88374. doi: 10.2196/88374 (PMC13161750; doi:10.2196/88374)
Supplement: Multimedia Appendix 1 [file jmir-v28-e88374-s001.docx]

# Table S1. Search strategy.

**S1.1 PubMed N =** **573**

| Steps | Search terms | Results |
| --- | --- | --- |
| #1 | "sarcopenia"[MeSH Terms] OR "sarcopeni*"[Title/Abstract] OR "frail"[Title/Abstract] OR "frailty"[Title/Abstract] OR "prefrail"[Title/Abstract] OR "prefrailty"[Title/Abstract] OR "pre-frail"[Title/Abstract] OR "pre-frailty"[Title/Abstract] | 71,818 |
| #2 | "muscle strength"[MeSH Terms] OR "muscle mass"[Title/Abstract] OR "muscle index"[Title/Abstract] OR "muscle size"[Title/Abstract] OR "muscle thickness"[Title/Abstract] OR "fat free mass"[Title/Abstract] OR "lean mass"[Title/Abstract] OR "muscle strength"[Title/Abstract] OR "muscular strength"[Title/Abstract] OR "muscle power"[Title/Abstract] OR "hand strength"[MeSH Terms] OR "handgrip strength"[Title/Abstract] OR "grip strength"[Title/Abstract] OR "Physical Activity"[Title/Abstract] OR "musc* function*"[Title/Abstract] OR "musc* size"[Title/Abstract] OR "musc* volume"[Title/Abstract] OR "physical fitness"[Title/Abstract] OR "physical function*"[Title/Abstract] OR "physical capacity"[Title/Abstract] OR "physical perform*"[Title/Abstract] | 376,961 |
| #3 | ((("Digital Health"[Mesh] OR "Internet"[Mesh] OR "Therapy, Computer-Assisted"[Mesh] OR "Mobile Applications"[Mesh] OR "Telemedicine"[Mesh] OR "Distance Counseling"[Mesh] OR "Computers, Handheld"[Mesh] OR "Video Games"[Mesh]) OR ("remote"[Title/Abstract] OR smartphone[Title/Abstract] OR phone[Title/Abstract] OR app[Title/Abstract] OR web[Title/Abstract] OR website[Title/Abstract] OR "information technology"[Title/Abstract] OR "distance counseling"[Title/Abstract] OR online*[Title/Abstract] OR tablet*[Title/Abstract] OR compute*[Title/Abstract] OR WAT[Title/Abstract] OR "Augmented Reality"[Title/Abstract] OR AR[Title/Abstract] OR "digital health"[Title/Abstract] OR "digital technology"[Title/Abstract] OR "telehealth literacy"[Title/Abstract] OR ehealth[Title/Abstract] OR "mobile health"[Title/Abstract] OR mhealth[Title/Abstract] OR "e-mail contact"[Title/Abstract] OR telecare[Title/Abstract] OR "mobile app"[Title/Abstract] OR "mobile application"[Title/Abstract] OR "online health"[Title/Abstract] OR internet[Title/Abstract] OR telehealth[Title/Abstract] OR telemedicine[Title/Abstract] OR software[Title/Abstract] OR fitbit[Title/Abstract] OR "fitness tracker"[Title/Abstract] OR "activity tracker"[Title/Abstract] OR "video game"[Title/Abstract] OR "computer game"[Title/Abstract] OR "virtual reality"[Title/Abstract] OR VR[Title/Abstract] OR "mixed reality"[Title/Abstract] OR exergame[Title/Abstract] OR "video gaming"[Title/Abstract] OR AI[Title/Abstract] OR Kinect[Title/Abstract] OR wii[Title/Abstract] OR tele[Title/Abstract])) OR (wearable[Title/Abstract])) | 2,378,565 |
| #4 | ((((Randomized controlled trial[Title/Abstract])) OR (Randomi*[Title/Abstract])) OR (trial[Title/Abstract])) OR (clinical trial[Title/Abstract]) | 1,431,415 |
| #5 | #1 AND #2 AND #3 ADN #4 | 573 |

**S1.2 Embase N = 1065**

| Steps | Search terms | Results |
| --- | --- | --- |
| #1 | 'Sarcopenia'/exp OR 'sarcopeni*':ab,ti OR 'frail':ab,ti OR 'frailty':ab,ti OR 'prefrail':ab,ti OR 'prefrailty':ab,ti OR 'pre-frail':ab,ti OR 'pre-frailty':ab,ti | 109,555 |
| #2 | 'muscle strength'/exp OR 'muscle mass':ab,ti OR 'muscle index':ab,ti OR 'muscle size':ab,ti OR 'muscle thickness':ab,ti OR 'fat free mass':ab,ti OR 'lean mass':ab,ti OR 'muscle strength':ab,ti OR 'muscular strength':ab,ti OR 'muscle power':ab,ti OR 'hand strength'/exp OR 'handgrip strength':ab,ti OR 'grip strength':ab,ti OR 'Physical Activity':ab,ti OR 'musc* function*':ab,ti OR 'musc* size':ab,ti OR 'musc* volume':ab,ti OR 'physical fitness':ab,ti OR 'physical function*':ab,ti OR 'physical capacity':ab,ti OR 'physical perform*':ab,ti | 568,680 |
| #3 | 'Digital Health'/exp OR 'Internet'/exp OR 'Therapy, Computer-Assisted'/exp OR 'Mobile Applications'/exp OR 'Telemedicine'/exp OR 'Distance Counseling'/exp OR 'Computers, Handheld'/exp OR 'Video Games'/exp OR 'remote':ab,ti OR smartphone:ab,ti OR phone:ab,ti OR app:ab,ti OR web:ab,ti OR website:ab,ti OR 'information technology':ab,ti OR 'distance counseling':ab,ti OR online*:ab,ti OR tablet*:ab,ti OR compute*:ab,ti OR WAT:ab,ti OR 'Augmented Reality':ab,ti OR AR:ab,ti OR 'digital health':ab,ti OR 'digital technology':ab,ti OR 'telehealth literacy':ab,ti OR ehealth:ab,ti OR 'mobile health':ab,ti OR mhealth:ab,ti OR 'e-mail contact':ab,ti OR telecare:ab,ti OR 'mobile app':ab,ti OR 'mobile application':ab,ti OR 'online health':ab,ti OR internet:ab,ti OR telehealth:ab,ti OR telemedicine:ab,ti OR software:ab,ti OR fitbit:ab,ti OR 'fitness tracker':ab,ti OR 'activity tracker':ab,ti OR 'video game':ab,ti OR 'computer game':ab,ti OR 'virtual reality':ab,ti OR VR:ab,ti OR 'mixed reality':ab,ti OR exergame:ab,ti OR 'video gaming':ab,ti OR AI:ab,ti OR Kinect:ab,ti OR wii:ab,ti OR tele:ab,ti OR wearable:ab,ti | 3,150,105 |
| #4 | ‘Randomized controlled trial’:ab,ti OR ‘Randomi*’:ab,ti OR ‘trial’:ab,ti OR ‘clinical trial’:ab,ti | 2,348,558 |
| #4 | #1 AND #2 AND #3 AND #4 | 1,065 |

**S1.3 Cochrane N = 565**

| Steps | Search terms | Results |
| --- | --- | --- |
| #1 | MeSH descriptor: [Sarcopenia] explode all trees | 1,119 |
| #2 | (sarcopeni*):ti,ab,kw OR (frail):ti,ab,kw OR (frailty):ti,ab,kw OR (prefrail):ti,ab,kw OR (prefrailty):ti,ab,kw OR (pre-frail):ti,ab,kw OR (pre-frailty):ti,ab,kw | 4,896 |
| #3 | #1 OR #2 | 5857 |
| #4 | MeSH descriptor: [Muscle Strength] explode all trees | 9,954 |
| #5 | MeSH descriptor: [Hand Strength] explode all trees | 2,724 |
| #6 | (muscle strength):ab,ti OR (muscle mass):ab,ti OR (muscle index):ab,ti OR (muscle size):ab,ti OR (muscle thickness):ab,ti OR (fat free mass):ab,ti OR (lean mass):ab,ti OR (muscle strength):ab,ti OR (muscular strength):ab,ti OR (muscle power):ab,ti OR (hand strength) OR (handgrip strength):ab,ti OR (grip strength):ab,ti OR (Physical Activity):ab,ti OR (musc* function*):ab,ti OR (musc* size):ab,ti OR (musc* volume):ab,ti OR (physical fitness):ab,ti OR (physical function*):ab,ti OR (physical capacity):ab,ti OR (physical perform*):ab,ti | 179,670 |
| #7 | #4 OR #5 OR #6 | 181,073 |
| #8 | MeSH descriptor: [Digital Health] explode all trees | 98 |
| #9 | MeSH descriptor: [Internet] explode all trees | 7,083 |
| #10 | MeSH descriptor: [Therapy, Computer-Assisted] explode all trees | 3,268 |
| #11 | MeSH descriptor: [Mobile Applications] explode all trees | 2,781 |
| #12 | MeSH descriptor: [Telemedicine] explode all trees | 5,942 |
| #13 | MeSH descriptor: [Distance Counseling] explode all trees | 26 |
| #14 | MeSH descriptor: [Computers, Handheld] explode all trees | 1,776 |
| #15 | MeSH descriptor: [Video Games] explode all trees | 1,417 |
| #16 | (remote):ab,ti OR (smartphone):ab,ti OR (phone):ab,ti OR (app):ab,ti OR (web):ab,ti OR (website):ab,ti OR (information technology):ab,ti OR (distance counseling):ab,ti OR (online*):ab,ti OR (tablet*):ab,ti OR (compute*):ab,ti OR (WAT):ab,ti OR (Augmented Reality):ab,ti OR (AR):ab,ti OR (digital health):ab,ti OR (digital technology):ab,ti OR (telehealth literacy):ab,ti OR (ehealth):ab,ti OR (mobile health):ab,ti OR (mhealth):ab,ti OR (e-mail contact):ab,ti OR (telecare):ab,ti OR (mobile app):ab,ti OR (mobile application):ab,ti OR (online health):ab,ti OR (internet):ab,ti OR (telehealth):ab,ti OR (telemedicine):ab,ti OR (software):ab,ti OR (fitbit):ab,ti OR (fitness tracker):ab,ti OR (activity tracker):ab,ti OR (video game):ab,ti OR (computer game):ab,ti OR (virtual reality):ab,ti OR (VR):ab,ti OR (mixed reality):ab,ti OR (exergame):ab,ti OR (video gaming):ab,ti OR (AI):ab,ti OR (Kinect):ab,ti OR (wii):ab,ti OR (tele):ab,ti OR (wearable):ab,ti | 269,486 |
| #17 | #8 OR #9 OR #10 OR #11 OR #12 OR #13 OR #14 OR #15 OR #16 | 272,777 |
| #18 | (Randomized controlled trial):ab,ti OR (Randomi*):ab,ti OR (trial):ab,ti OR (clinical trial):ab,ti | 1,329,770 |
| #19 | #3 AND #7 AND #17 AND #18 | 565 |

**S1.4 Supplement table 4- Web of Science N = 654**

| Steps | Search terms | Results |
| --- | --- | --- |
| #1 | TS=("sarcopenia" OR "sarcopeni*" OR "frail" OR "frailty" OR "prefrail" OR "prefrailty" OR "pre-frail" OR "pre-frailty") | 82,731 |
| #2 | TS=("muscle strength" OR "muscle mass"OR "muscle index"OR "muscle size"OR "muscle thickness"OR "fat free mass"OR "lean mass"OR "muscle strength"OR "muscular strength"OR "muscle power"OR "hand strength" OR "handgrip strength"OR "grip strength"OR "Physical Activity"OR "musc* function*"OR "musc* size"OR "musc* volume"OR "physical fitness"OR "physical function*"OR "physical capacity"OR "physical perform*" ) | 396,825 |
| #3 | TS=("Digital Health" OR "Internet" OR "Therapy, Computer-Assisted" OR "Mobile Applications" OR "Telemedicine" OR "Distance Counseling" OR "Computers, Handheld" OR "Video Games" OR "remote" OR “smartphone” OR “phone” OR “app” OR “web” OR “website” OR "information technology" OR "distance counseling" OR “online*” OR “tablet*” OR “compute*” OR “WAT” OR "Augmented Reality" OR AR OR "digital health" OR "digital technology" OR "telehealth literacy" OR ehealth OR "mobile health" OR “mhealth” OR "e-mail contact" OR “telecare” OR "mobile app" OR "mobile application" OR "online health" OR ”internet” OR “telehealth” OR “telemedicine” OR “software” OR “fitbit” OR "fitness tracker" OR "activity tracker" OR "video game" OR "computer game" OR "virtual reality" OR VR OR "mixed reality" OR “exergame” OR "video gaming" OR “AI” OR “Kinect” OR “wii” OR “tele” OR “wearable”) | 3,851,467 |
| #4 | TS= (“Randomized controlled trial” OR “Randomi*” OR “trial” OR “clinical trial”) | 1,865,784 |
| #5 | #1 AND #2 AND #3 AND #4 | 654 |

**S1.5 Supplement table 5 Ovid MEDLINE N = 633**

| Steps | Search terms | Results |
| --- | --- | --- |
| #1 | **sarcopenia** | 14222 |
| #2 | XB Sarcopeni* or frailty or frail or prefrailty or prefrail or pre-frailty or pre-frail | 76384 |
| #3 | #1 OR #2 | 31507 |
| #4 | Muscle Strength/ | 24173 |
| #5 | Hand Strength/ | 392845 |
| #6 | (muscle strength or muscle mass or muscle index or muscle size or muscle thickness or fat free mass or lean mass or muscle strength or muscular strength or muscle power or hand strength or handgrip strength or grip strength or Physical Activity or musc* function* or musc* size or musc* volume or physical fitness or physical function* or physical capacity or physical perform*) | 76384 |
| #7 | #4 OR #5 OR #6 | 392845 |
| #8 | Digital Health | 1292 |
| #9 | Internet | 87653 |
| #10 | Therapy, Computer-Assisted | 7074 |
| #11 | Mobile Applications | 16390 |
| #12 | Telemedicine | 47574 |
| #13 | Distance Counseling | 82 |
| #14 | Computers, Handheld | 4284 |
| #15 | Video Games | 8760 |
| #16 | (remote or smartphone or phone or app or web or website or information technology or distance counseling or online* or tablet* or compute* or WAT or Augmented Reality or AR or digital health or digital technology or telehealth literacy or ehealth or mobile health or mhealth or e-mail contact or telecare or mobile app or mobile application or online health or internet or telehealth or telemedicine or software or fitbit or fitness tracker or activity tracker or video game or computer game or virtual reality or VR or mixed reality or exergame or video gaming or AI or Kinect or wii or tele or wearable) | 3156105 |
| #17 | 8 or 9 or 10 or 11 or 12 or 13 or 14 or 15 or 16 | 3158545 |
| #18 | Randomized controlled trial or Randomi* or trial or clinical trial | 1973613 |
| #19 | 3 and 7 and 17 and 18 | 633 |

Note: The last search dates for the above databases were all January 11, 2026.
