# Supplementary material for: Effectiveness of Digital Health Interventions in Older Adults With Frailty and Sarcopenia: Systematic Review and Meta‐Analysis of Randomized Controlled Trials
Source: J Med Internet Res. 2026 May 11;28:e88374. doi: 10.2196/88374 (PMC13161750; doi:10.2196/88374)
Supplement: Multimedia Appendix 2 [file jmir-v28-e88374-s002.docx]

**Author(s):** Ting Dai, Changsheng Guo, Lingli Gao, Jinyu Huang, Yan Chen, Yujie Zhang, Jing Gao, Xiaodong Feng

**Question:** Effectiveness of Digital Health Interventions in Older Adults with Frailty and Sarcopenia

**Setting:** Hospitals, senior care centers, communities, and homes

**Bibliography:**

| **Certainty assessment** | | | | | | | **№ of patients** | | **Effect** | | **Certainty** | **Importance** |
| --- | --- | --- | --- | --- | --- | --- | --- | --- | --- | --- | --- | --- |
| **№ of studies** | **Study design** | **Risk of bias** | **Inconsistency** | **Indirectness** | **Imprecision** | **Other considerations** | **[Digital Health Interventions]** | **[comparison]** | **Relative (95% CI)** | **Absolute (95% CI)** |  |  |
| **grip strength** | | | | | | | | | | | | |
| 10 | randomised trials | serious^a^ | not serious | not serious | serious^b,c^ | none | 369 | 324 | - | MD **0.49 SD higher** (0.43 lower to 1.41 higher) | ⨁⨁◯◯ Low^a,b,c^ | CRITICAL |
| **skeletal muscle mass** | | | | | | | | | | | | |
| 3 | randomised trials | serious^a^ | serious^d^ | not serious | serious^c^ | none | 204 | 108 | - | MD **1.01 higher** (0.08 higher to 1.94 higher) | ⨁◯◯◯ Very low^a,c,d^ | CRITICAL |
| **Skeletal muscle mass index** | | | | | | | | | | | | |
| 8 | randomised trials | serious^a^ | serious^e^ | not serious | serious^b^ | none | 366 | 220 | - | MD **0.18 higher** (0.04 lower to 0.4 higher) | ⨁◯◯◯ Very low^a,b,e^ | CRITICAL |
| **gait speed** | | | | | | | | | | | | |
| 8 | randomised trials | serious^a^ | not serious | not serious | serious^c^ | none | 490 | 306 | - | MD **0.09 SD higher** (0.03 higher to 0.15 higher) | ⨁⨁◯◯ Low^a,c^ | CRITICAL |
| **30-second chair stand test** | | | | | | | | | | | | |
| 5 | randomised trials | serious^a^ | serious^d^ | not serious | serious^c^ | none | 141 | 107 | - | MD **2.19 higher** (0.89 higher to 5.66 higher) | ⨁◯◯◯ Very low^a,c,d^ | CRITICAL |
| **Timed Up and Go Test** | | | | | | | | | | | | |
| 9 | randomised trials | serious^a^ | not serious | not serious | serious^c^ | none | 354 | 265 | - | MD **0.52 lower** (1.02 lower to 0.03 lower) | ⨁⨁◯◯ Low^a,c^ | CRITICAL |
| **Balance** | | | | | | | | | | | | |
| 6 | randomised trials | serious^a^ | serious^f^ | not serious | serious^c^ | none | 385 | 259 | - | SMD **0.61 SD higher** (0 to 1.21 higher) | ⨁◯◯◯ Very low^a,c,f^ | CRITICAL |
| **quality of Life** | | | | | | | | | | | | |
| 6 | randomised trials | serious^a^ | not serious | not serious | serious^c^ | none | 268 | 218 | - | SMD **0.16 SD higher** (0.05 higher to 0.27 higher) | ⨁⨁◯◯ Low^a,c^ | CRITICAL |

**CI:** confidence interval; **MD:** mean difference; **SMD:** standardised mean difference

#### Explanations

a. Some studies fail to adequately describe the implementation of randomization procedures, allocation concealment, and blinding of outcome assessors.

b. The 95% confidence interval for the pooled effect estimate is wide and crosses the line of no effect.

c. In terms of sample size, there are significant differences among the studies. Some studies have relatively small sample sizes, which may lead to wider confidence intervals for effect estimates, making the estimates less precise.

d. Differences in research design, such as intervention measures, study populations, and outcome indicators, led to significant inconsistency between the results of one study and those of the other studies.

e. There are significant inconsistencies in the results between different studies.

f. High heterogeneity
