# Supplementary material for: Effectiveness of Digital Health Interventions in Older Adults With Frailty and Sarcopenia: Systematic Review and Meta‐Analysis of Randomized Controlled Trials
Source: J Med Internet Res. 2026 May 11;28:e88374. doi: 10.2196/88374 (PMC13161750; doi:10.2196/88374)
Supplement: Multimedia Appendix 3 [file jmir-v28-e88374-s003.docx]

Forest Plot Meta-Analyses for Different Outcomes

Figure S1. The effects of digital health interventions on grip strength compared with control groups.


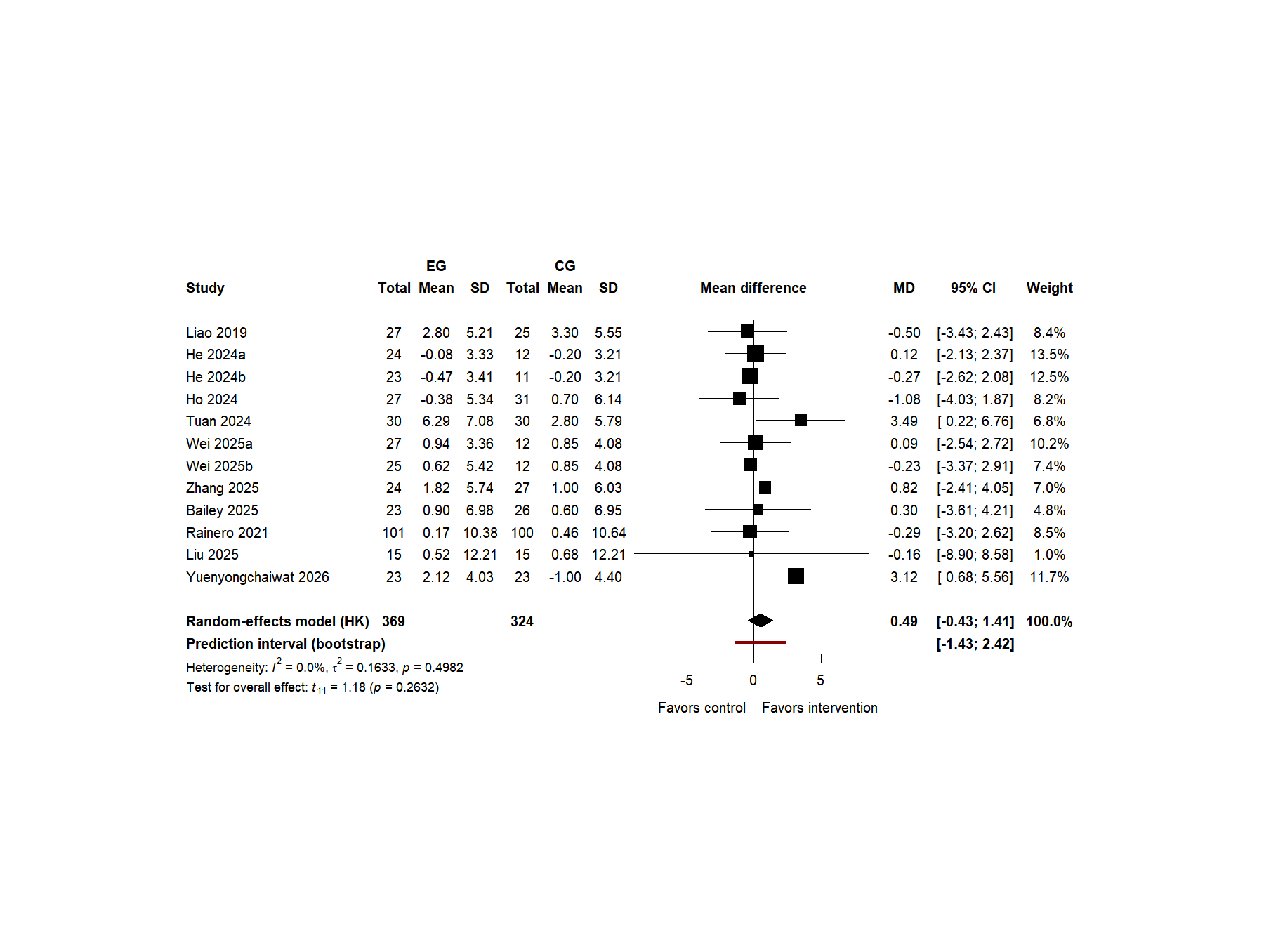


Note: CG: control group; EG: experimental group; HK: Hartung-Knapp (method); MD: mean difference; Labels a and b denote distinct intervention arms from the same multi‑arm trial. To avoid double counting, the sample size of the shared control group was split equally between intervention arms (He 2024a/b, Wei 2025a/b).

Figure S2. The Effects of digital health interventions on Grip Strength Compared with Control Groups: A 3-Month Follow-Up Study


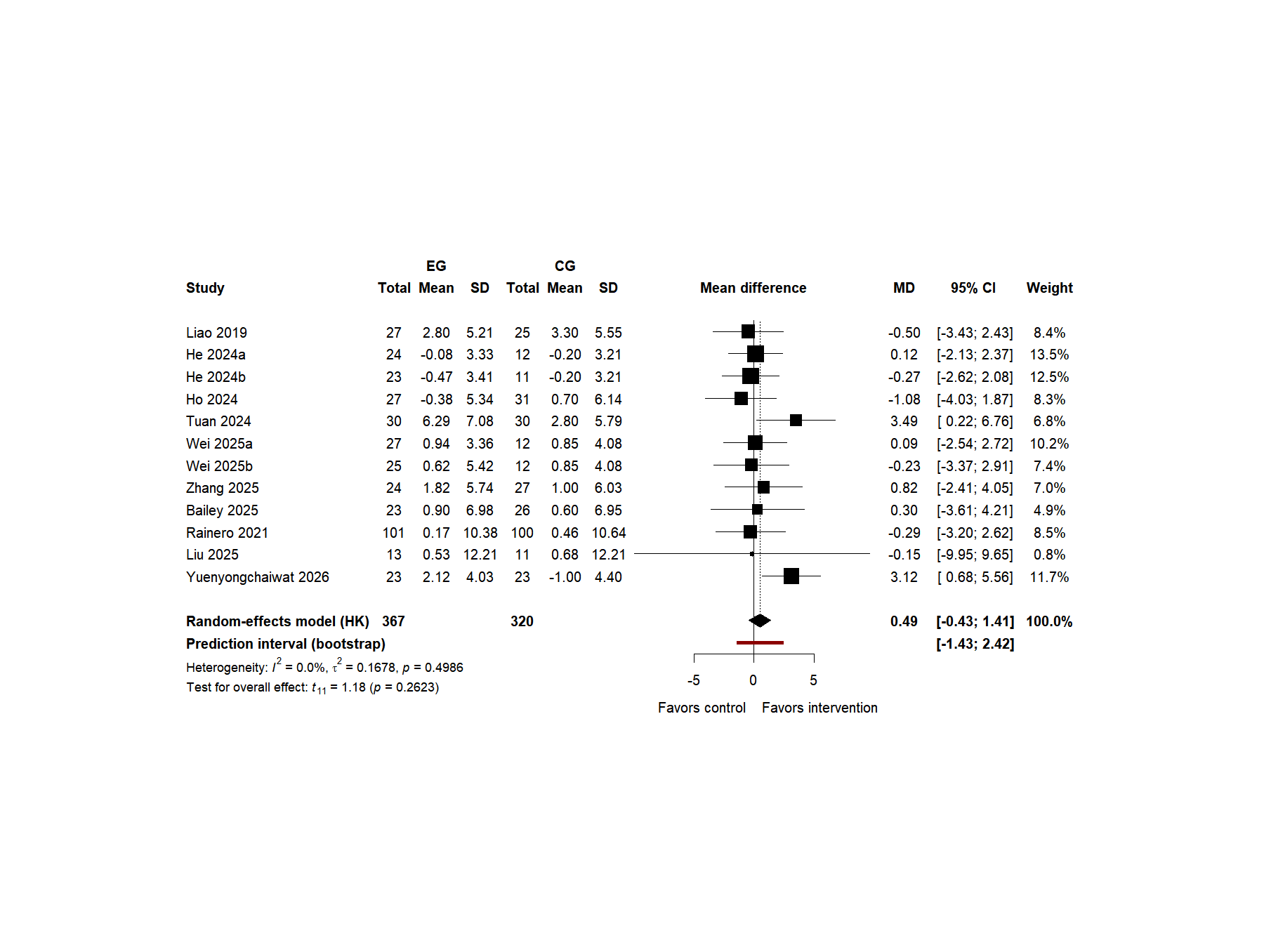


Note: CG: control group; EG: experimental group; HK: Hartung-Knapp (method); MD: mean difference; Labels a and b denote distinct intervention arms from the same multi‑arm trial. To avoid double counting, the sample size of the shared control group was split equally between intervention arms (He 2024a/b, Wei 2025a/b).

Figure S3. The effects of digital health interventions on skeletal muscle mass compared with control groups.


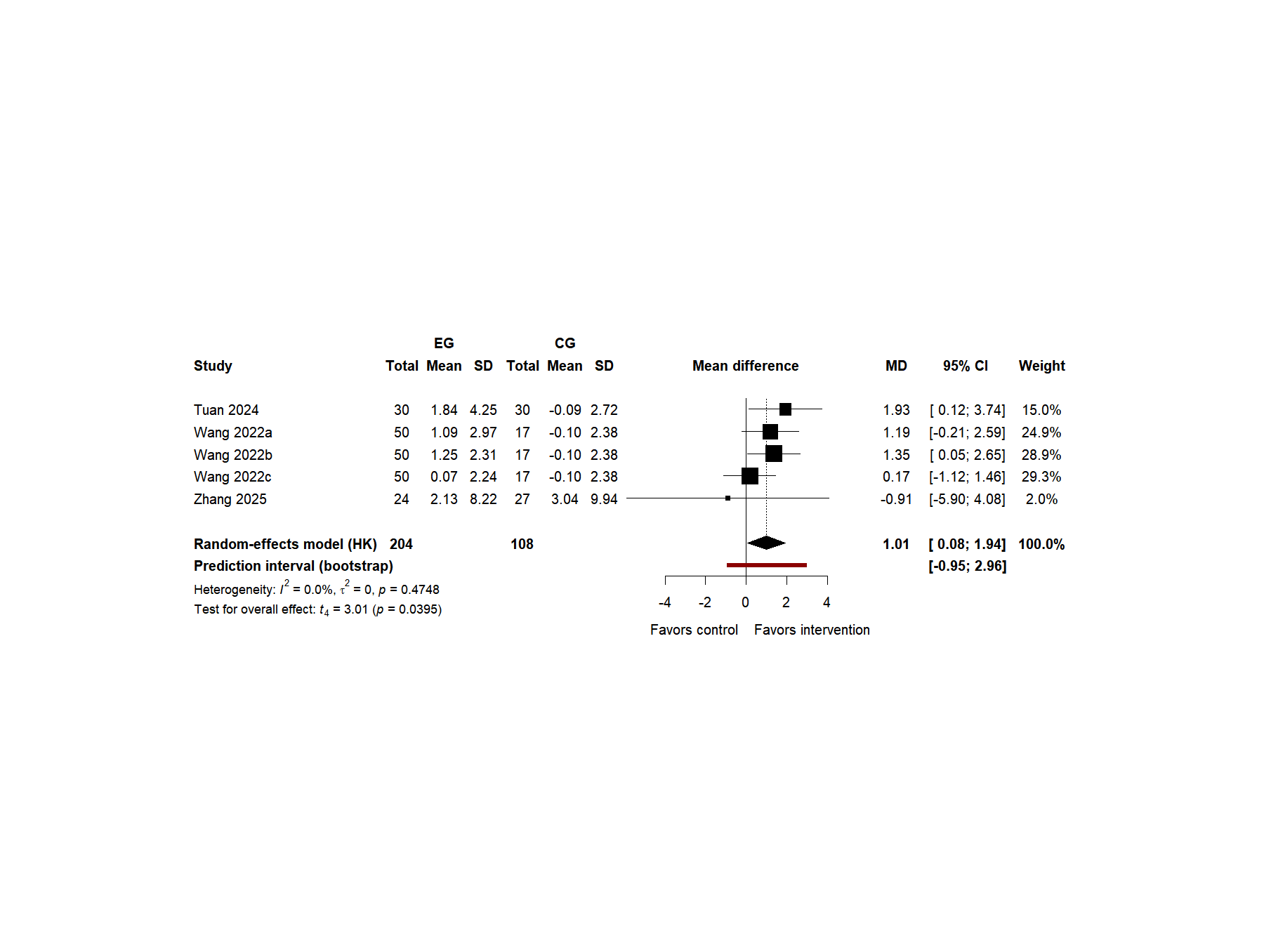


Note: CG: control group; EG: experimental group; HK: Hartung-Knapp (method); MD: mean difference; Labels a, b, and c denote distinct intervention arms from the same multi‑arm trial. To avoid double counting, the sample size of the shared control group was split equally between intervention arms (Wang 2022a/b/c).

Figure S4. The effects of digital health interventions on ASMI compared with control groups.


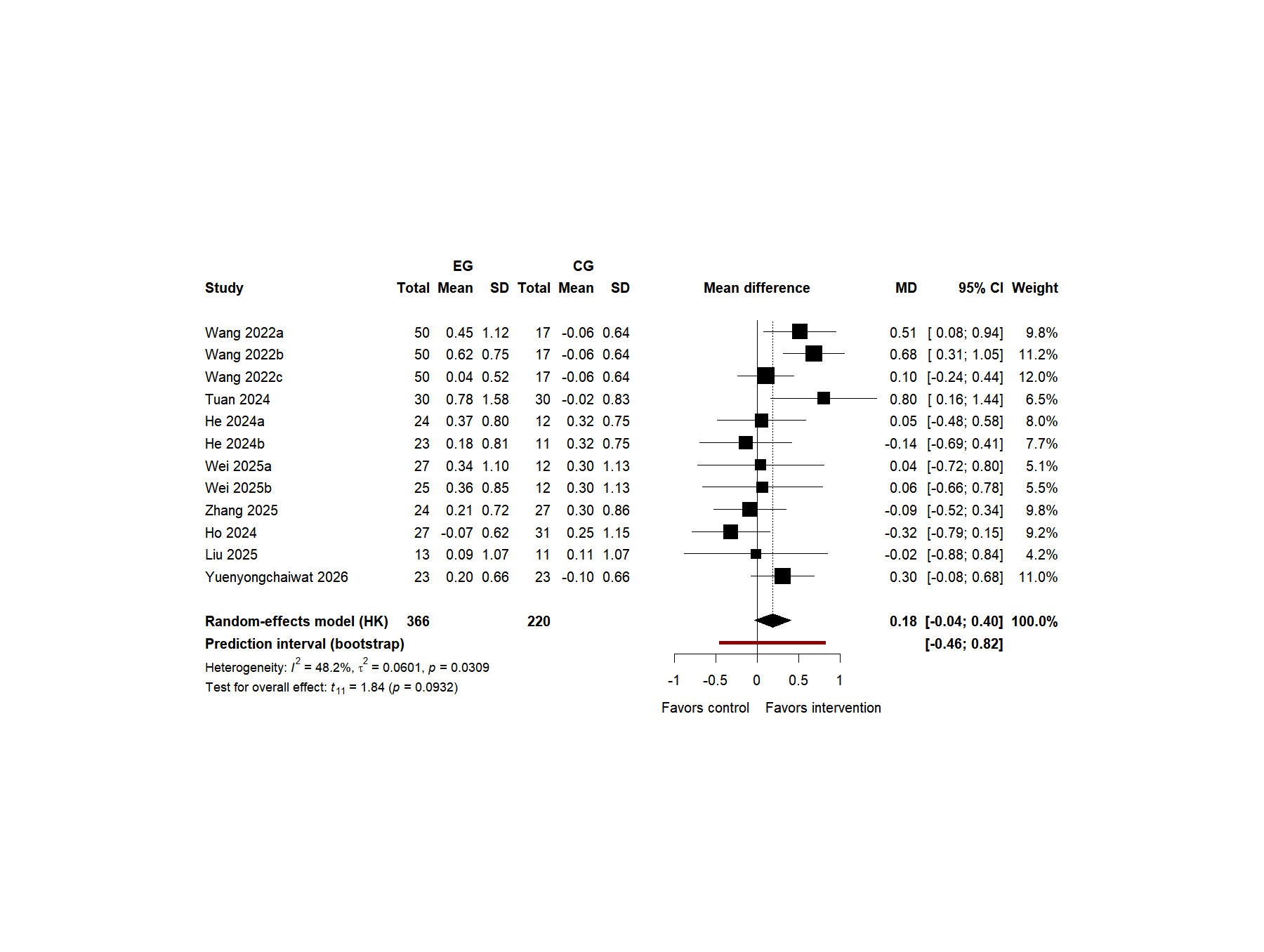


Note: CG: control group; EG: experimental group; HK: Hartung-Knapp (method); MD: mean difference; Labels a, b, and c denote distinct intervention arms from the same multi‑arm trial. To avoid double counting, the sample size of the shared control group was split equally between intervention arms (Wang 2022a/b/c, He 2024a/b, Wei 2025a/b).

Figure S5. The effects of digital health interventions on ASMI compared with control groups: A 3-Month Follow-Up Study


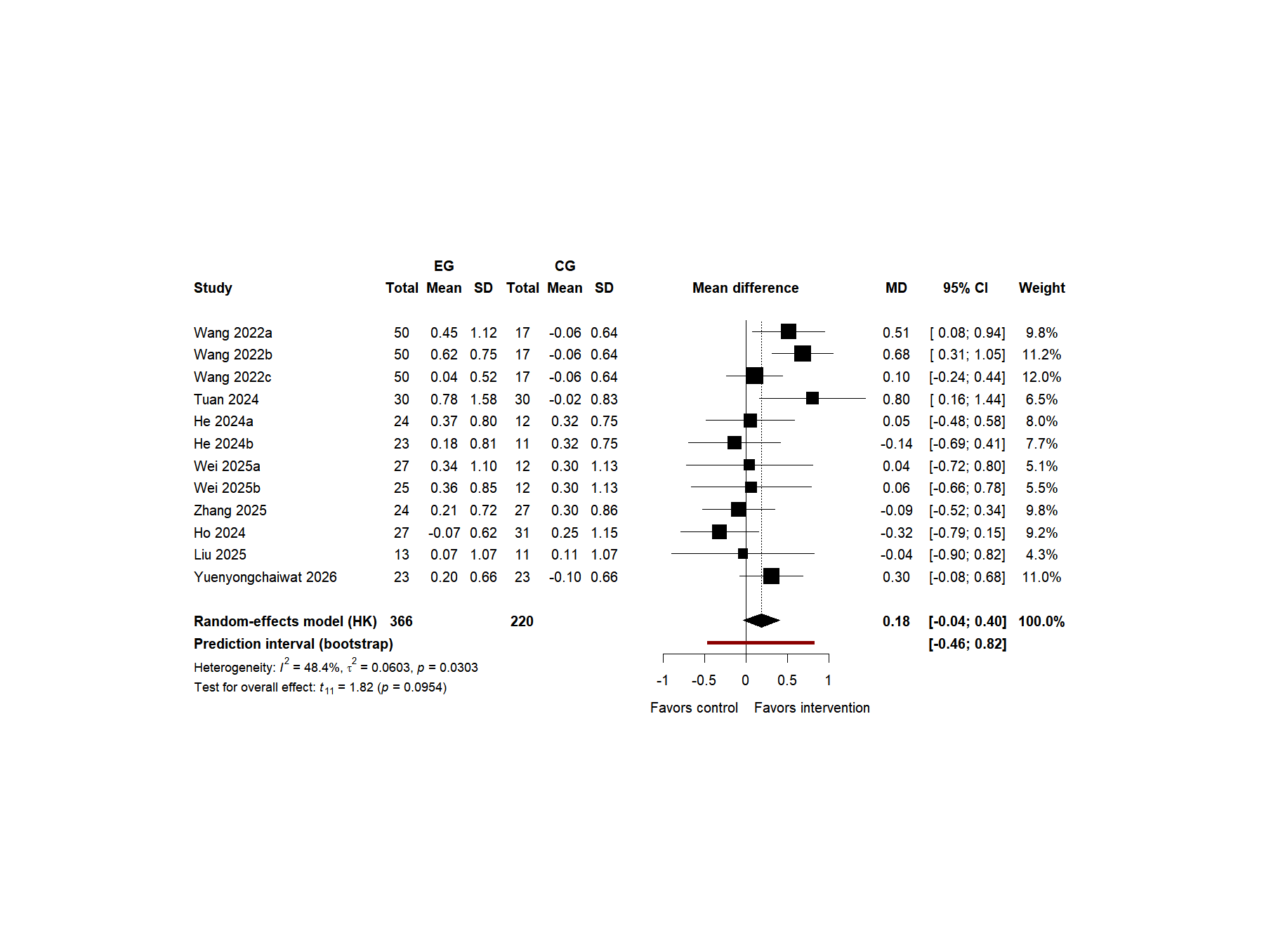


Note: CG: control group; EG: experimental group; HK: Hartung-Knapp (method); MD: mean difference; Labels a, b, and c denote distinct intervention arms from the same multi‑arm trial. To avoid double counting, the sample size of the shared control group was split equally between intervention arms (Wang 2022a/b/c, He 2024a/b, Wei 2025a/b).

Figure S6. The effects of digital health interventions on gait speed compared with control groups.


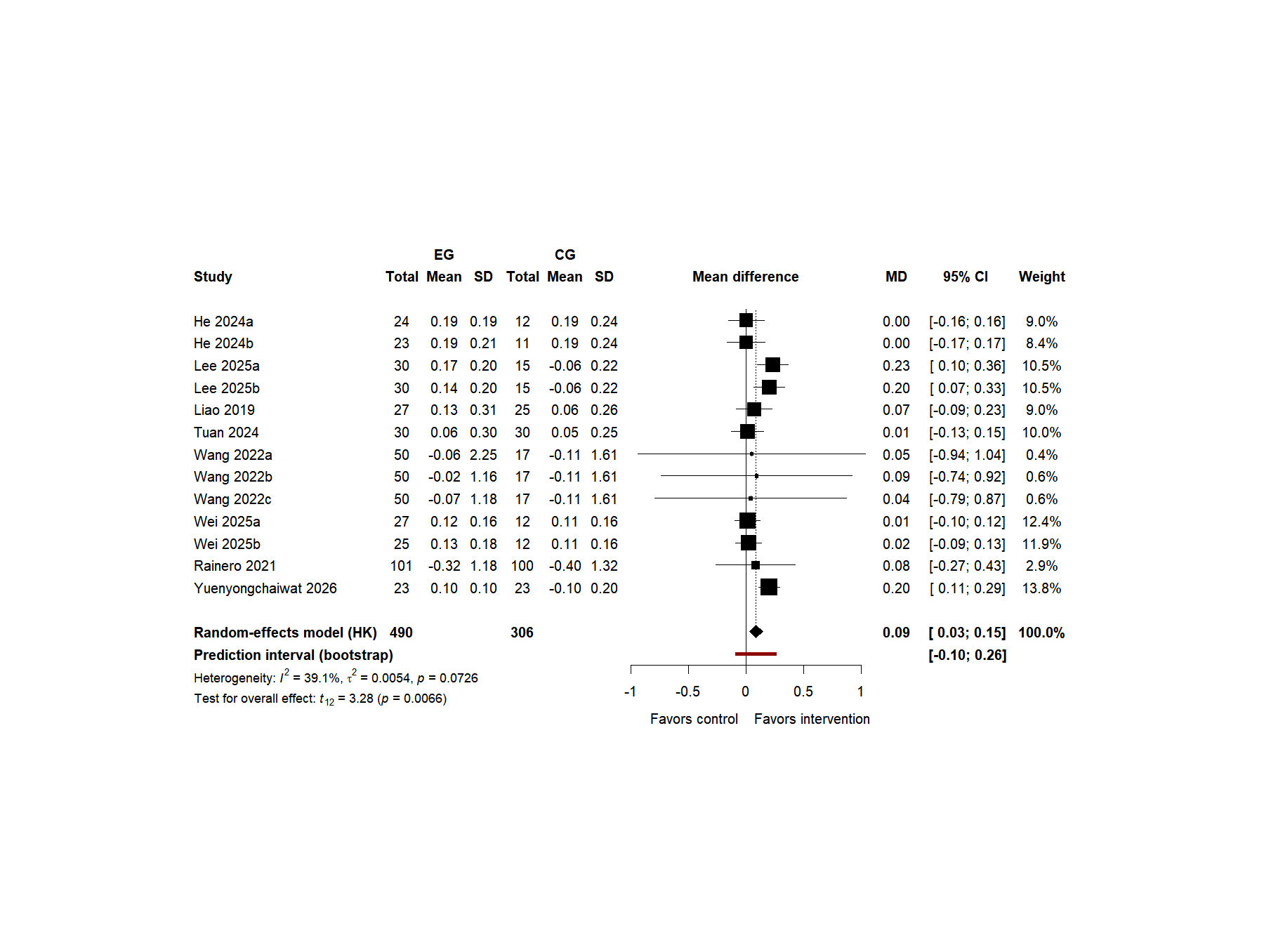


Note: CG: control group; EG: experimental group; HK: Hartung-Knapp (method); MD: mean difference; Labels a, b, and c denote distinct intervention arms from the same multi‑arm trial. To avoid double counting, the sample size of the shared control group was split equally between intervention arms (He 2024a/b, Lee 2025a/b, Wang 2022a/b/c, Wei 2025a/b).

Figure S7. The effects of Digital Health Interventions on the 2-Minute Walk Test compared with control groups.


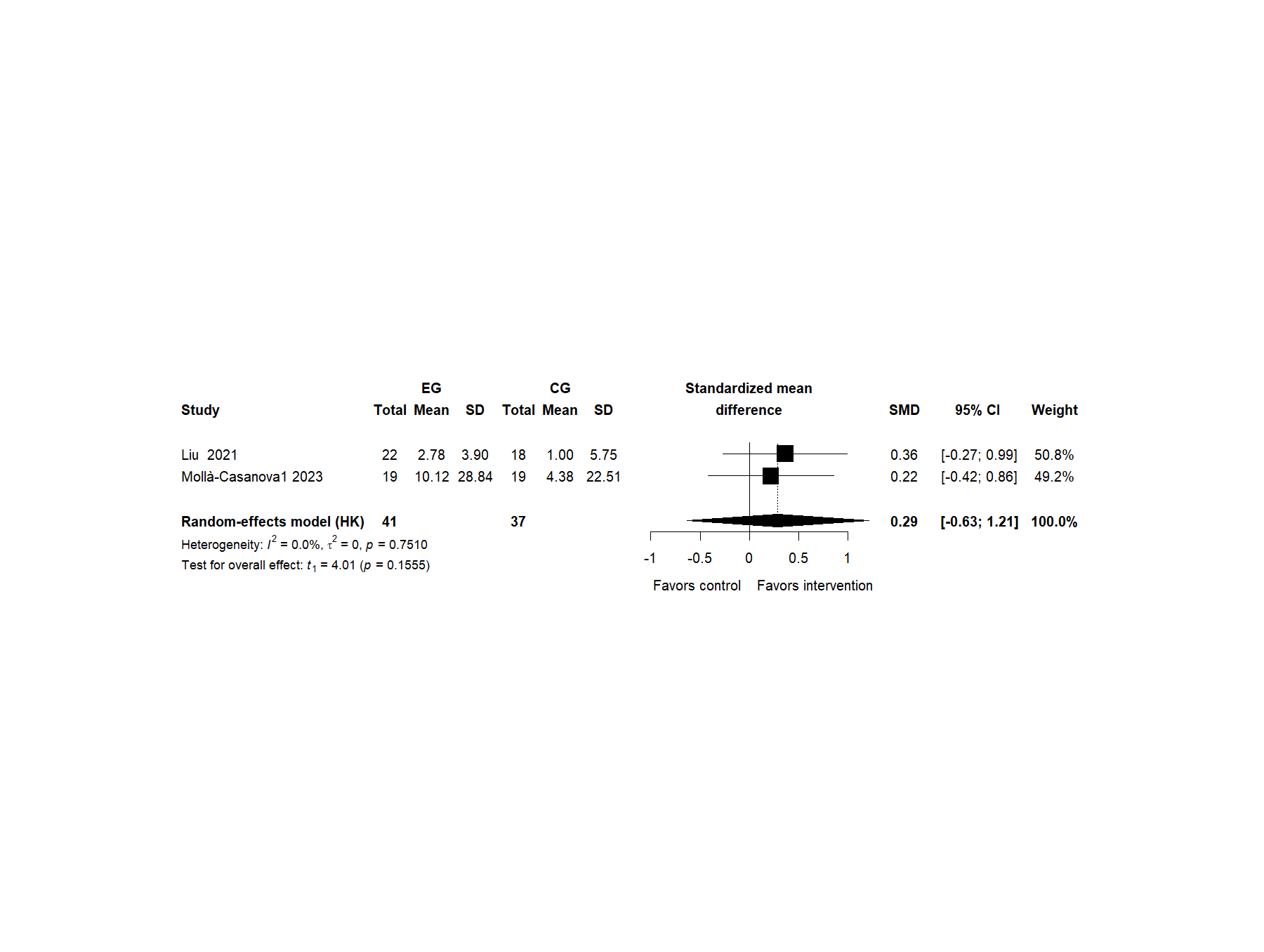


Note: CG: control group; EG: experimental group; HK: Hartung-Knapp (method); SMD: standardized mean difference.

Figure S8. Effects of Digital Health Interventions on the 2-Minute Walk Test During Follow-up: A Comparison with Control Groups


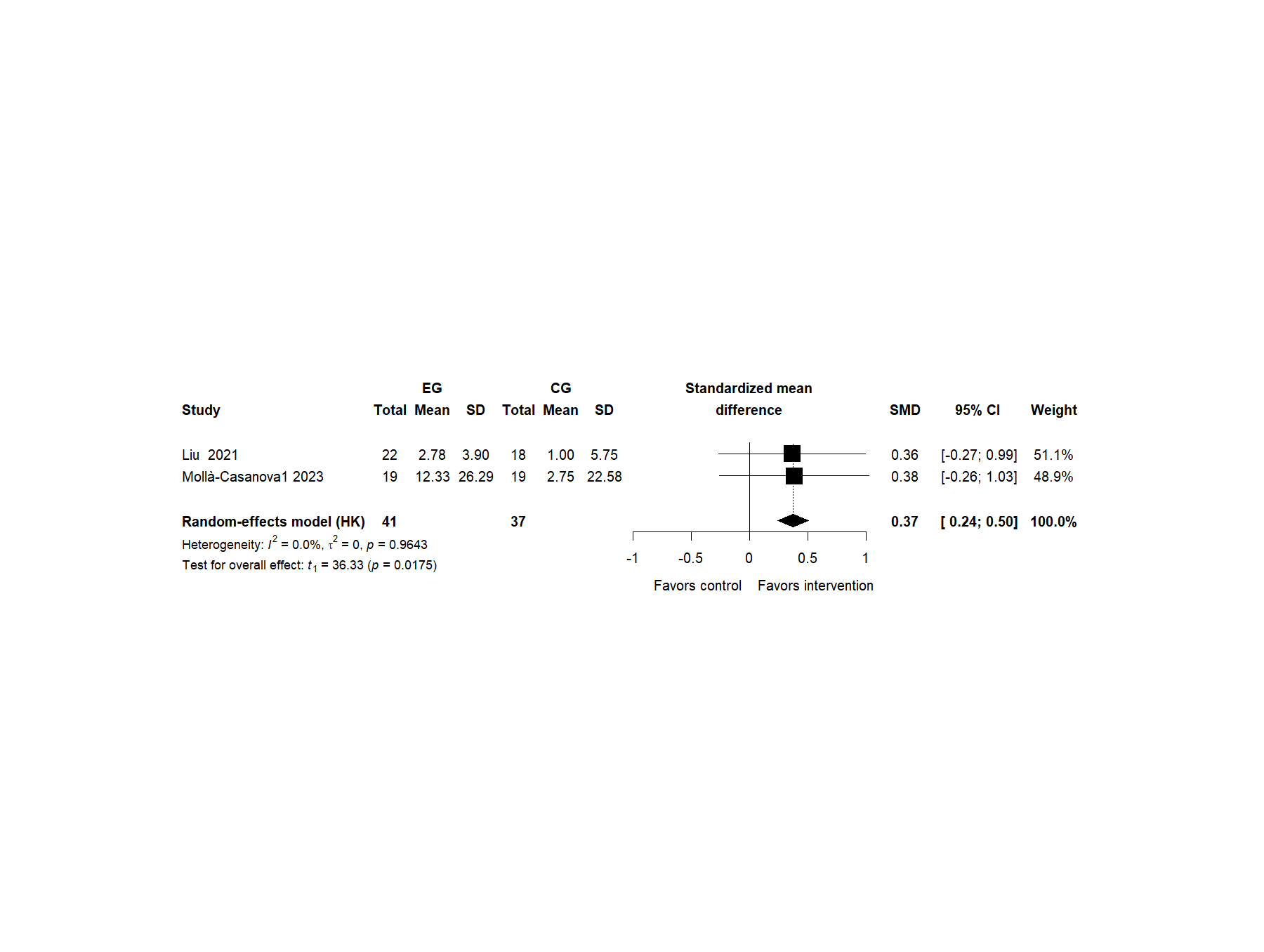


Note: CG: control group; EG: experimental group; HK: Hartung-Knapp (method); SMD: standardized mean difference.

Figure S9. The effects of digital health interventions on Six-Minute Walk Test compared with control groups.


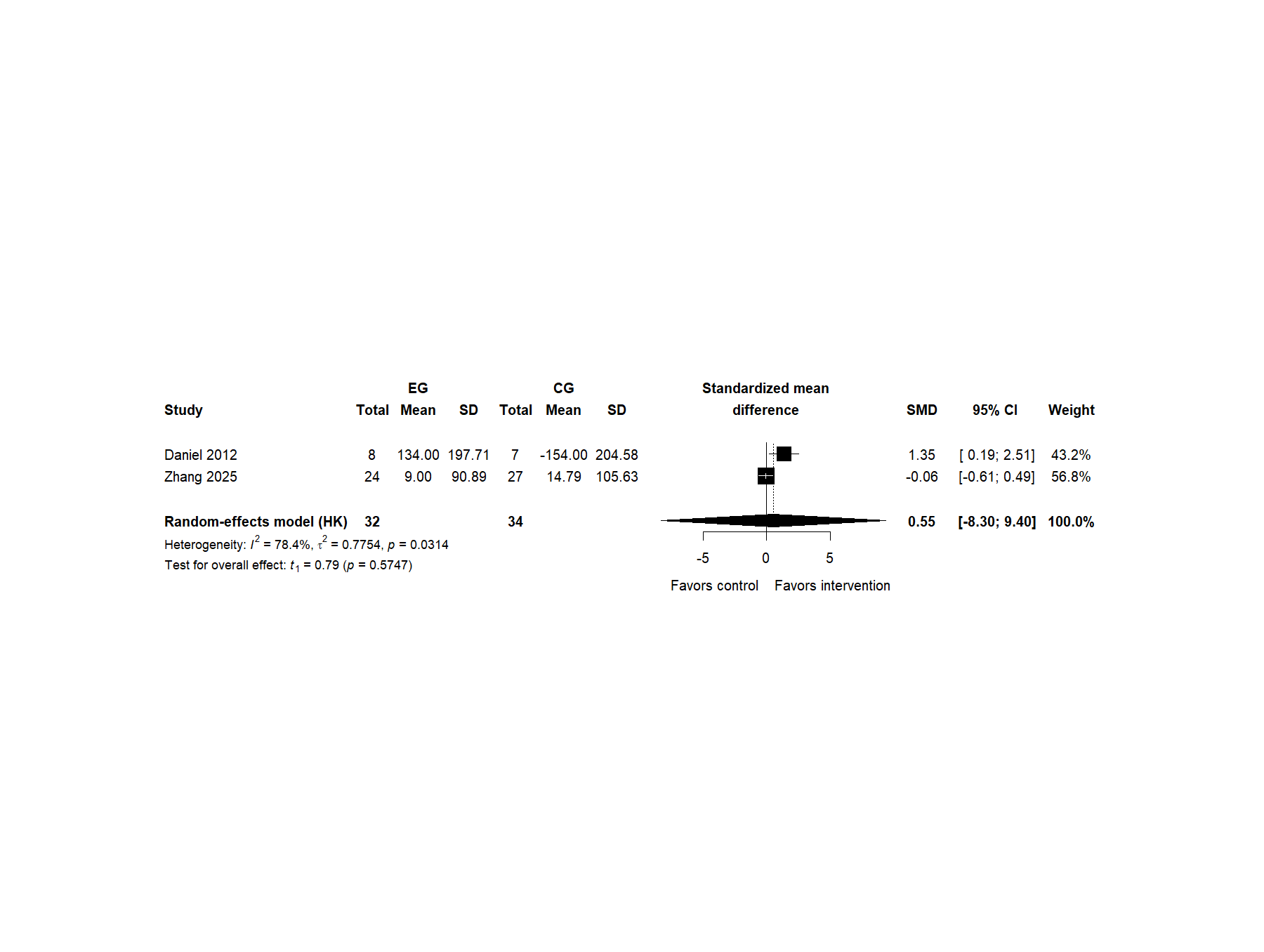


Note: CG: control group; EG: experimental group; HK: Hartung-Knapp (method); SMD: standardized mean difference.

Figure S10. The effects of digital health interventions on Timed Up and Go Test compared with control groups.


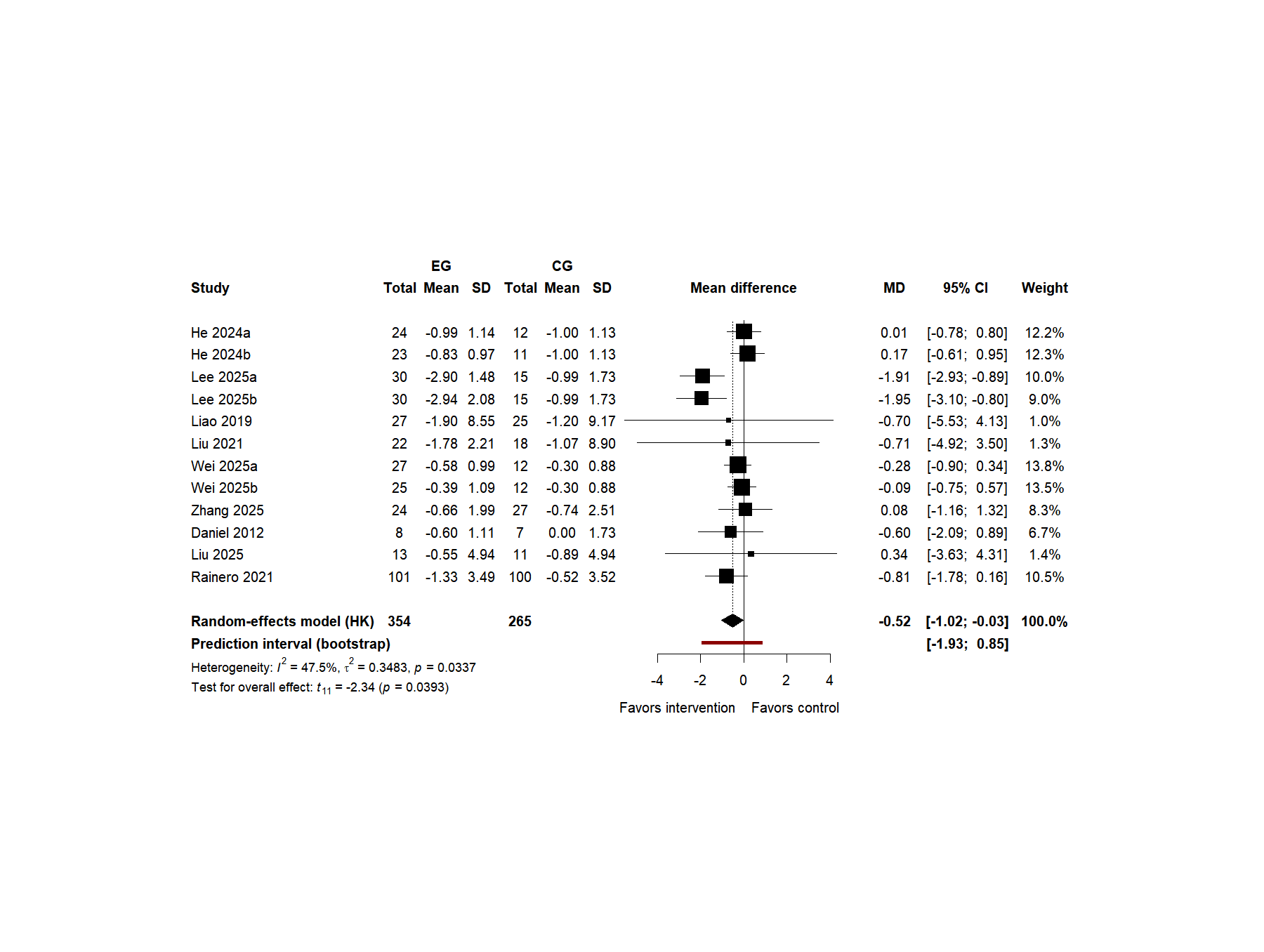


Note: CG: control group; EG: experimental group; HK: Hartung-Knapp (method); MD: mean difference; Labels a, b, and c denote distinct intervention arms from the same multi‑arm trial. To avoid double counting, the sample size of the shared control group was split equally between intervention arms (He 2024a/b, Lee 2025a/b, Wei 2025a/b).

Figure S11. The effects of digital health interventions on Timed Up and Go Test compared with control groups: A 3-Month Follow-Up Study


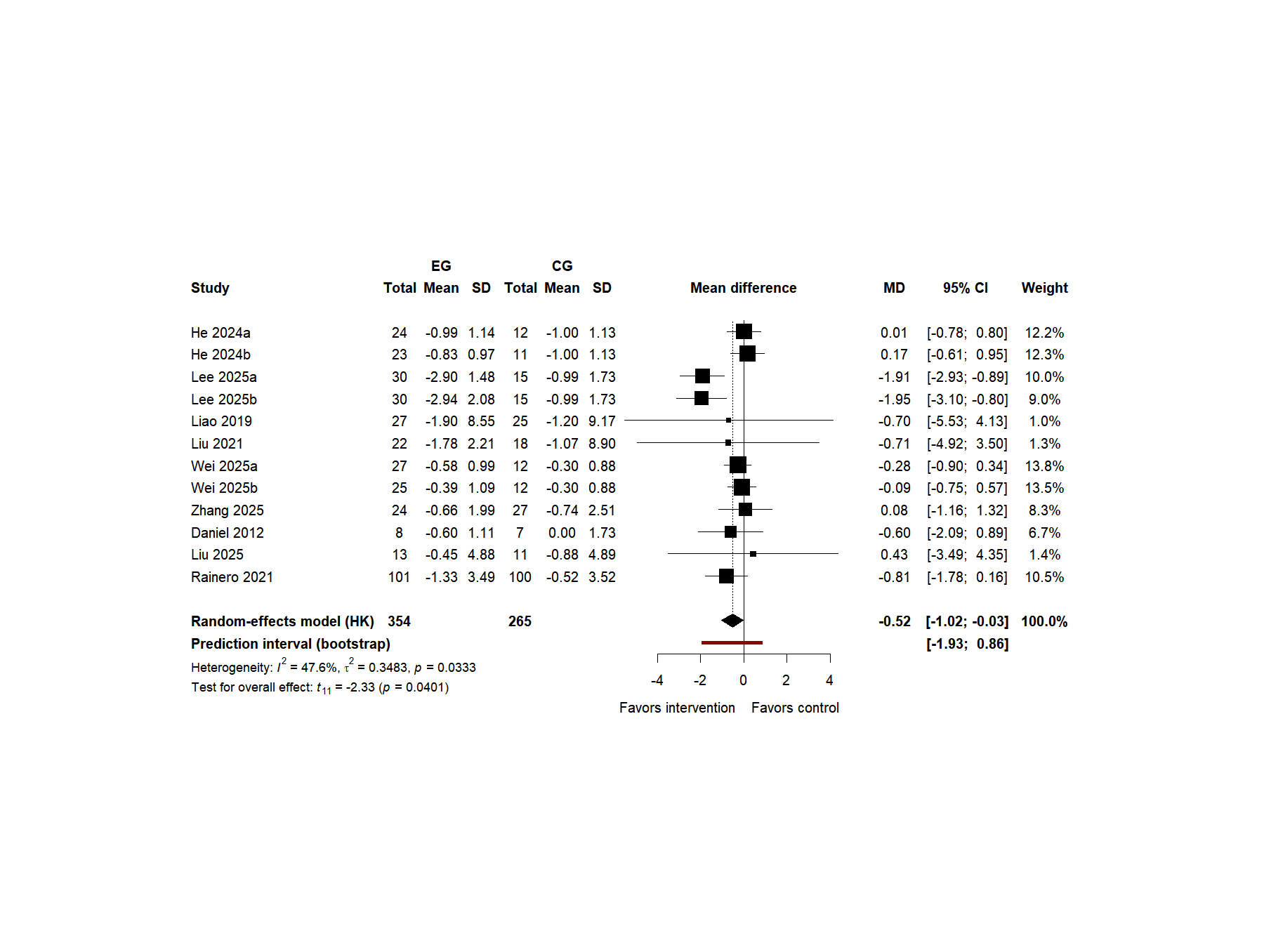


Note: CG: control group; EG: experimental group; HK: Hartung-Knapp (method); MD: mean difference; Labels a, b, and c denote distinct intervention arms from the same multi‑arm trial. To avoid double counting, the sample size of the shared control group was split equally between intervention arms (He 2024a/b, Lee 2025a/b, Wei 2025a/b).

Figure S12. The effects of digital health interventions on Five Times Sit-to-Stand Test compared with control groups.


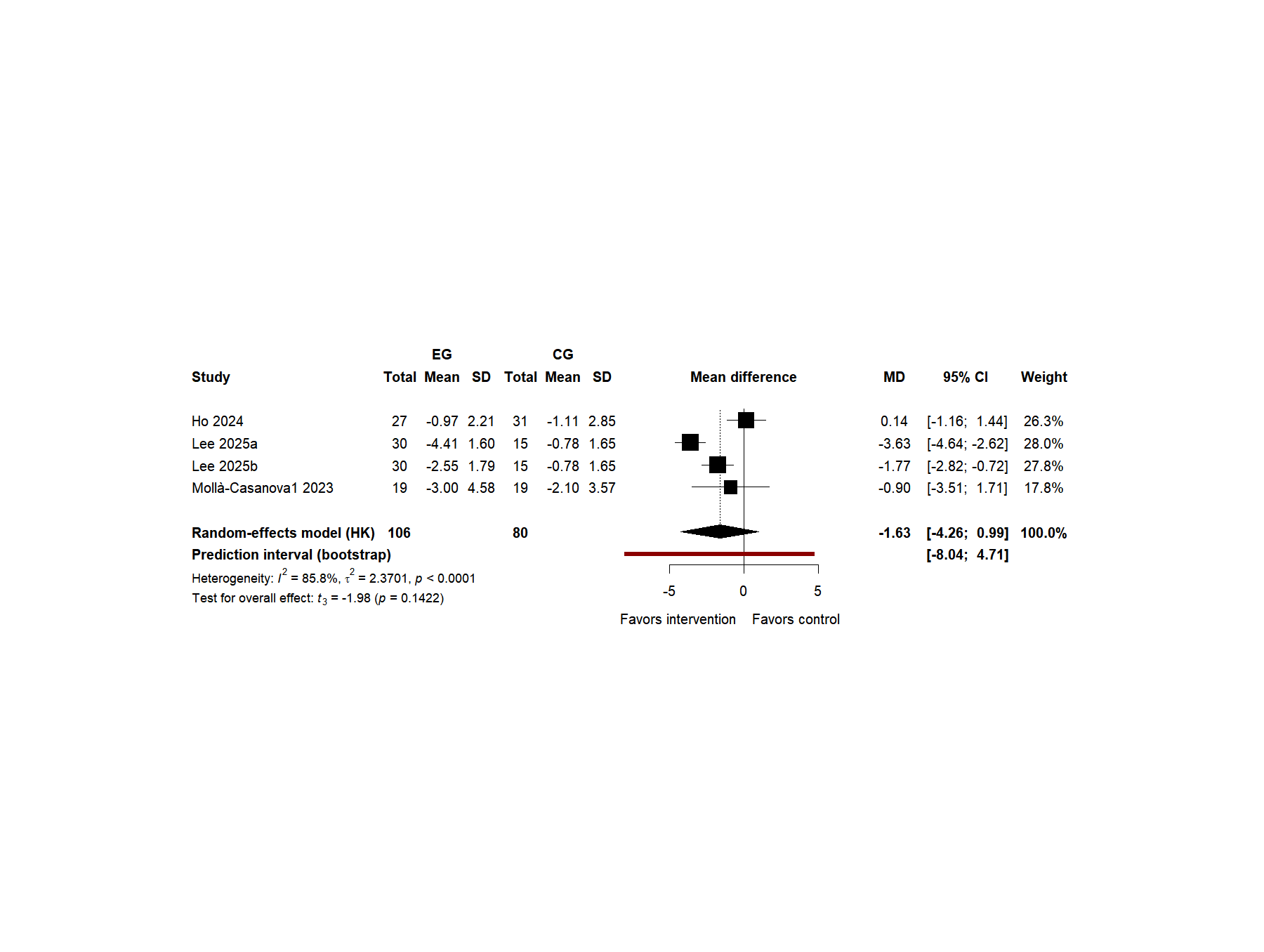


Note: CG: control group; EG: experimental group; HK: Hartung-Knapp (method); MD: mean difference; Labels a, b, and c denote distinct intervention arms from the same multi‑arm trial. To avoid double counting, the sample size of the shared control group was split equally between intervention arms (Lee 2025a/b).

S13. The effects of digital health interventions on Five Times Sit-to-Stand Test compared with control groups: A 1 Month Follow-Up Study


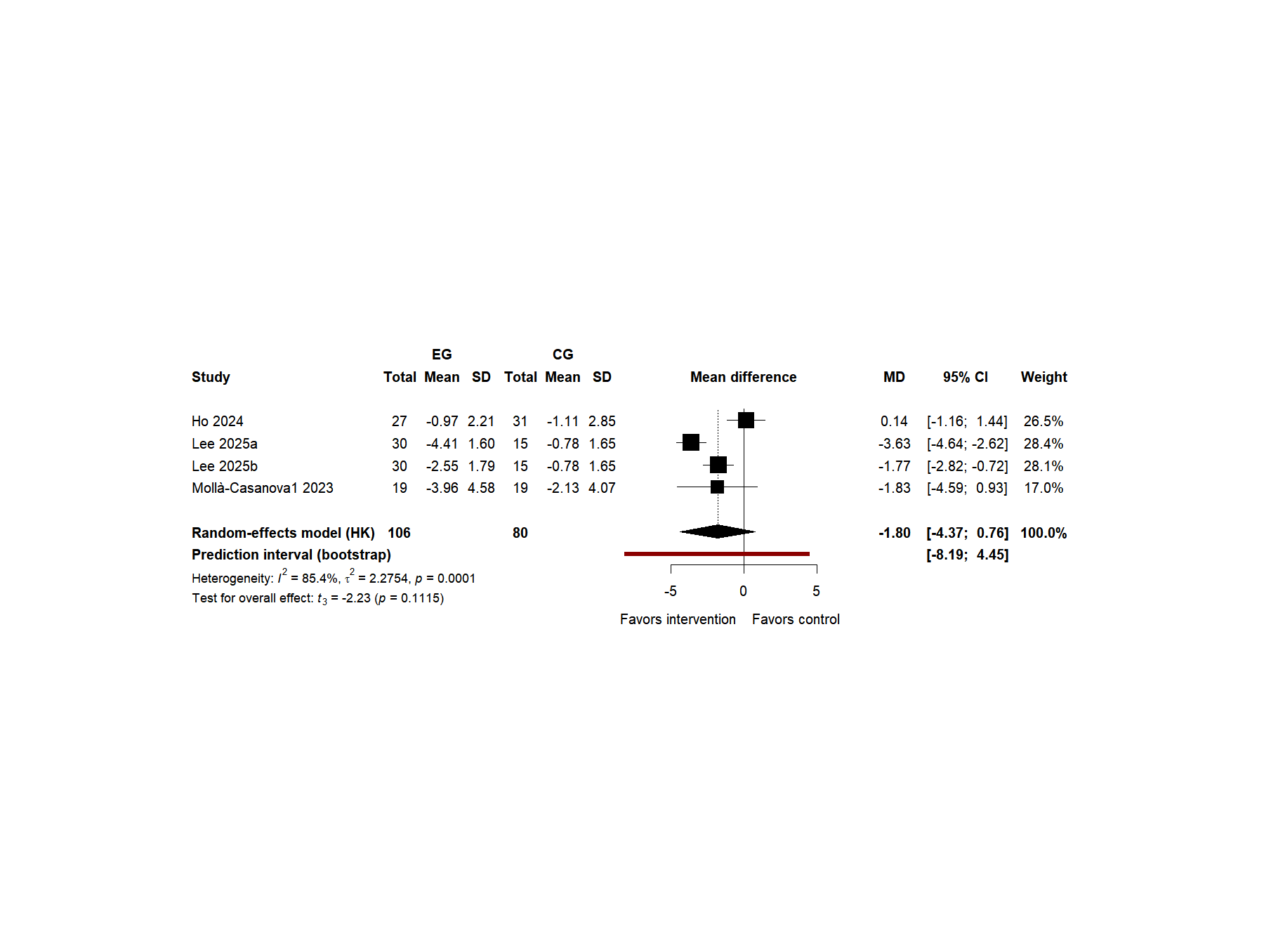


Note: CG: control group; EG: experimental group; HK: Hartung-Knapp (method); MD: mean difference; Labels a, b, and c denote distinct intervention arms from the same multi‑arm trial. To avoid double counting, the sample size of the shared control group was split equally between intervention arms (Lee 2025a/b).

S14. The effects of digital health interventions on 30-Second Chair Stand Test compared with control groups.


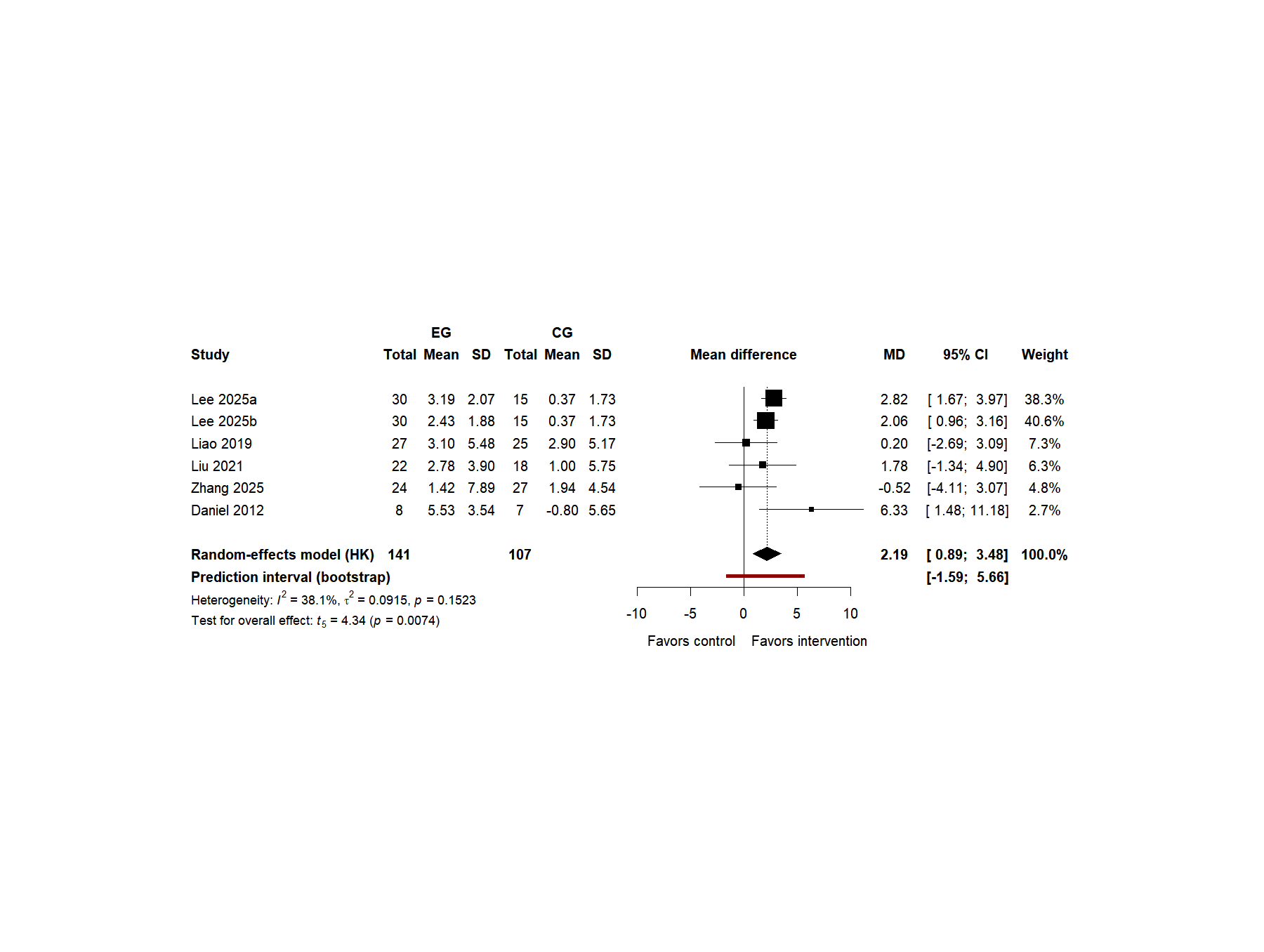


Note: CG: control group; EG: experimental group; HK: Hartung-Knapp (method); MD: mean difference; Labels a, b, and c denote distinct intervention arms from the same multi‑arm trial. To avoid double counting, the sample size of the shared control group was split equally between intervention arms (Lee 2025a/b).

S15. The effects of digital health interventions on Balance compared with control groups.


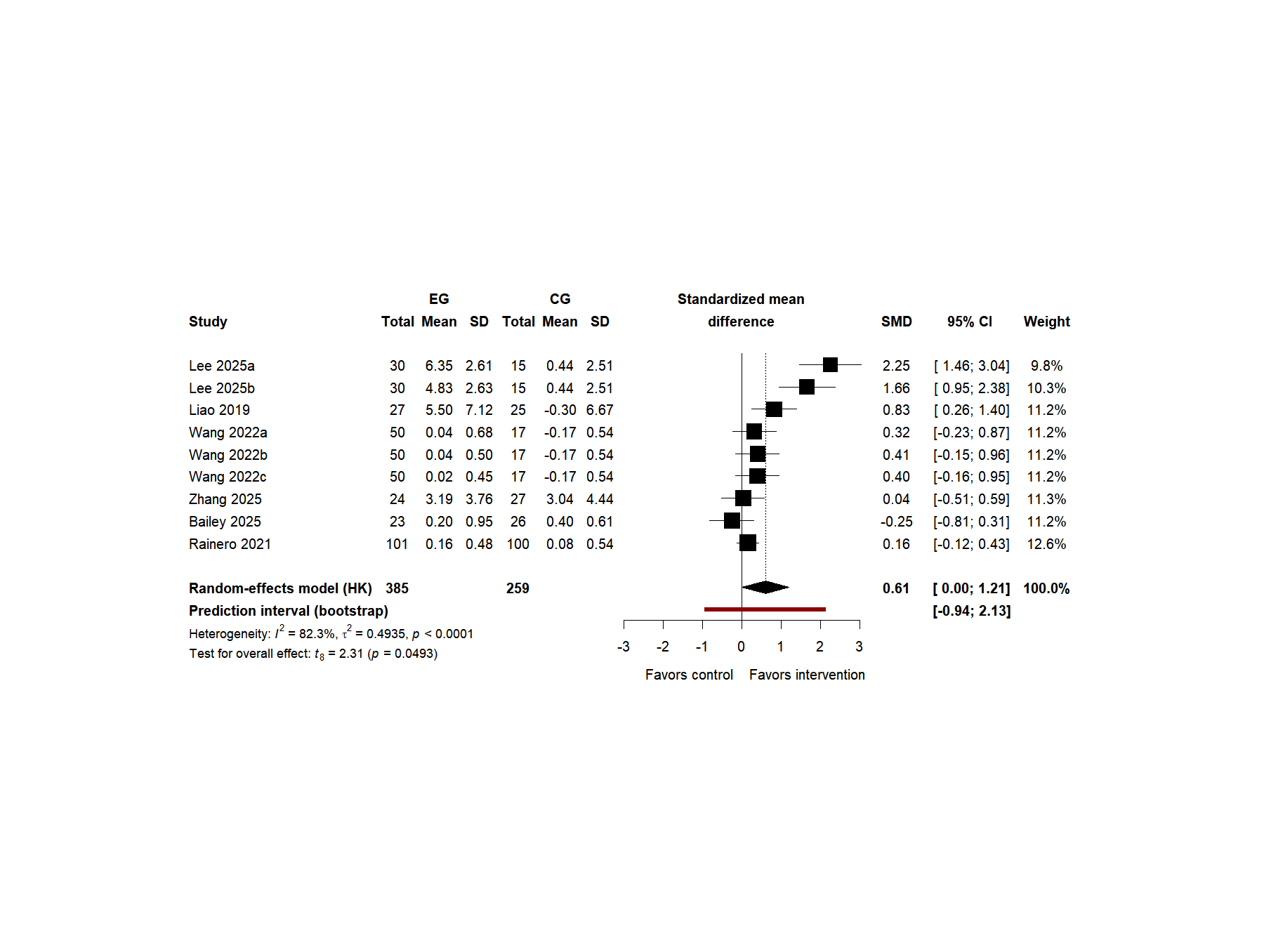


Note: CG: control group; EG: experimental group; HK: Hartung-Knapp (method); SMD: standardized mean difference; Labels a, b, and c denote distinct intervention arms from the same multi‑arm trial. To avoid double counting, the sample size of the shared control group was split equally between intervention arms (Lee 2025a/b, Wang 2025a/b/c).

S16 The effects of digital health interventions on Short Physical Performance Battery compared with control groups.


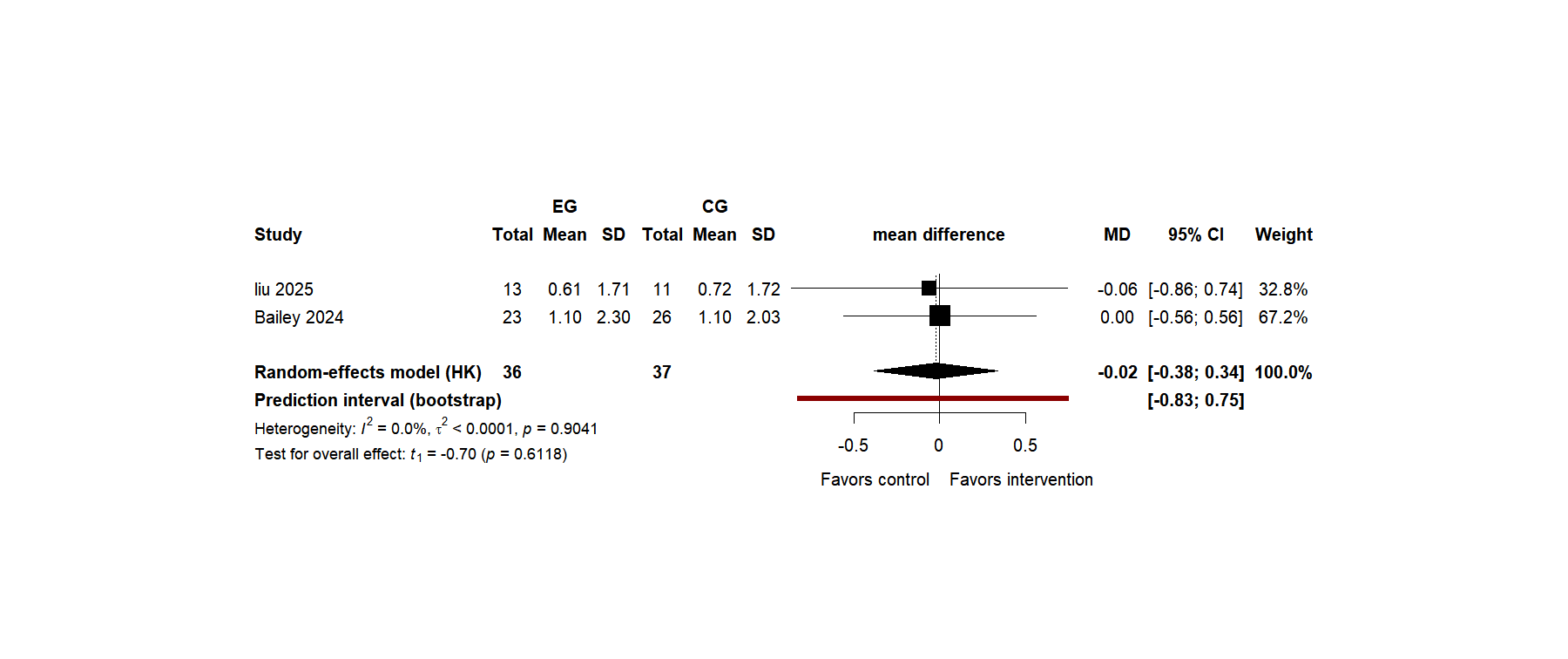


Note: CG: control group; EG: experimental group; HK: Hartung-Knapp (method); MD: mean difference.

S17 The effects of digital health interventions on Short Physical Performance Battery compared with control groups: A 3-Month Follow-Up Study.


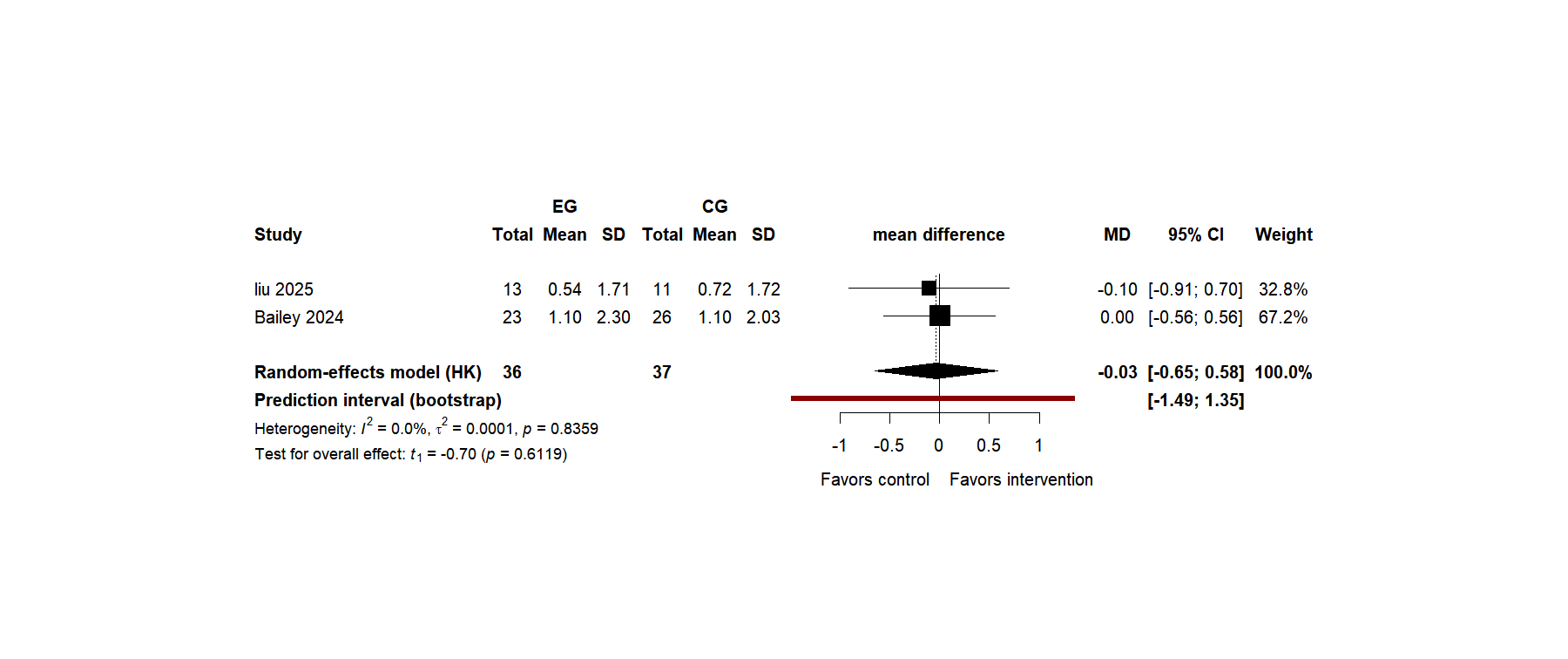


Note: CG: control group; EG: experimental group; HK: Hartung-Knapp (method); MD: mean difference.

S18. The effects of digital health interventions on Quality of Life compared with control groups.


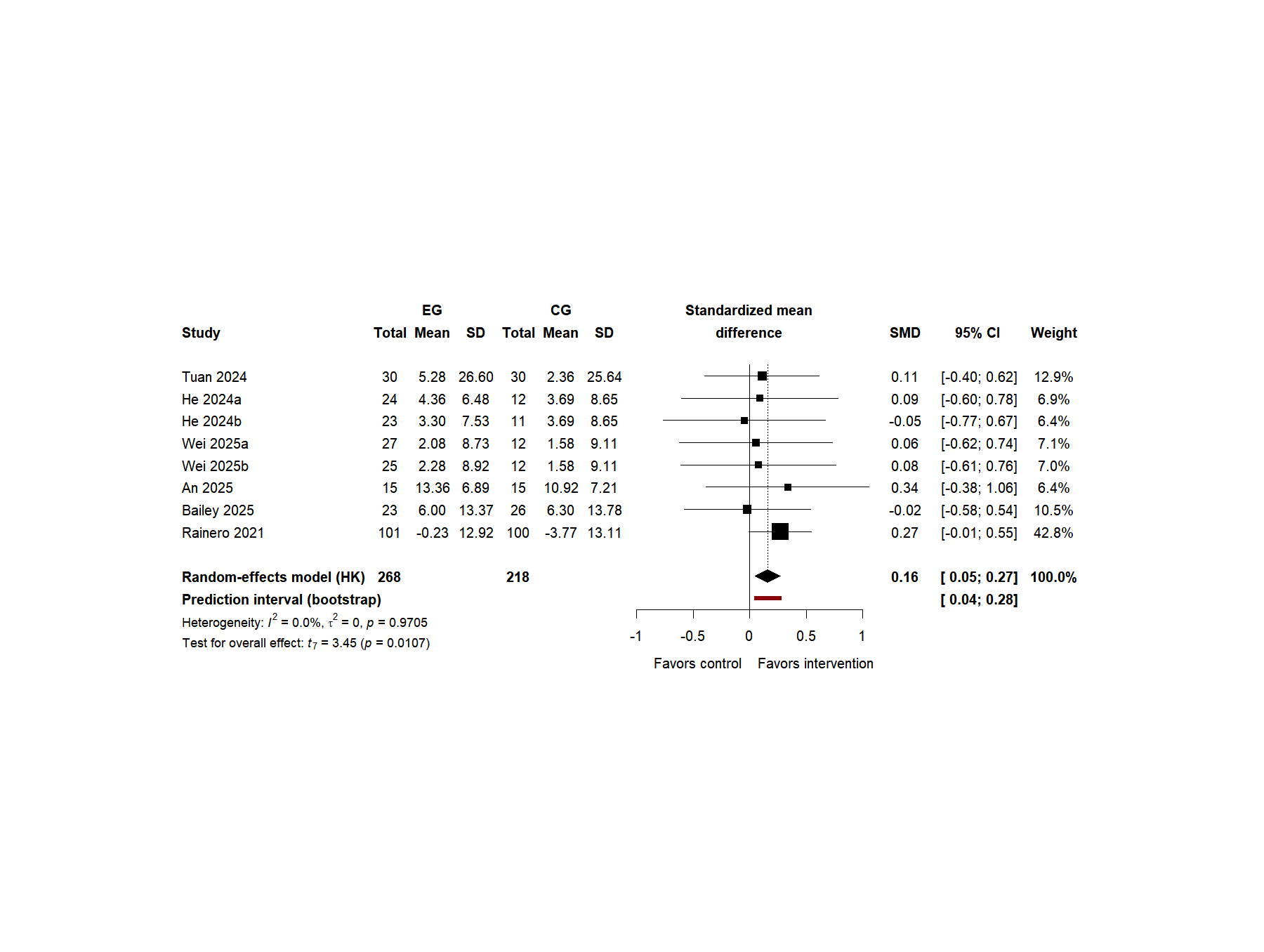


Note: CG: control group; EG: experimental group; HK: Hartung-Knapp (method); SMD: standardized mean difference; Labels a, b, and c denote distinct intervention arms from the same multi‑arm trial. To avoid double counting, the sample size of the shared control group was split equally between intervention arms (He 2024a/b, Wei 2025a/b).

S19. The effects of digital health interventions on body mass index compared with control groups.


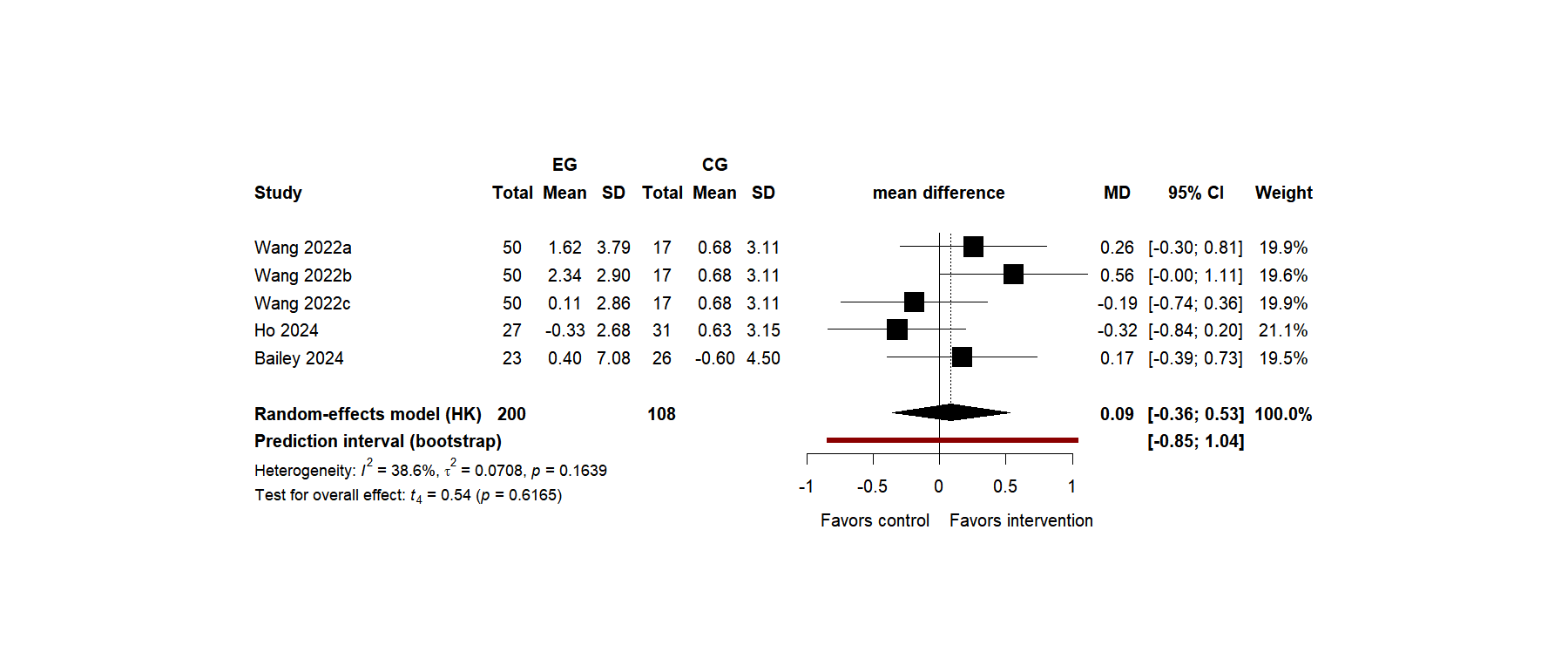


Note: CG: control group; EG: experimental group; HK: Hartung-Knapp (method); MD: mean difference; Labels a, b, and c denote distinct intervention arms from the same multi‑arm trial. To avoid double counting, the sample size of the shared control group was split equally between intervention arms (Wang 2025a/b/c).
