# Supplementary material for: Effectiveness of Digital Health Interventions in Older Adults With Frailty and Sarcopenia: Systematic Review and Meta‐Analysis of Randomized Controlled Trials
Source: J Med Internet Res. 2026 May 11;28:e88374. doi: 10.2196/88374 (PMC13161750; doi:10.2196/88374)
Supplement: Multimedia Appendix 4 [file jmir-v28-e88374-s004.docx]

**Forest Plot of Subgroup Analysis**

Note: CG: control group; EG: experimental group; HK: Hartung-Knapp (method); MD:mean difference; SMD: standardized mean difference; Labels a, b and c denote distinct intervention arms from the same multi-arm trial. To avoid double counting, the sample size of the shared control group was split equally between intervention arms (He 2024a/b, Wei 2025a/b, Wang 2022a/b/c, Lee 2025a/b).

**Figure 1. Subgroup Analyses of Each Outcome Based on Control Group Type**

**1.1. Subgroup Analysis of Appendicular Skeletal Muscle Mass Index (ASMI) Based on Control Group Type**

**
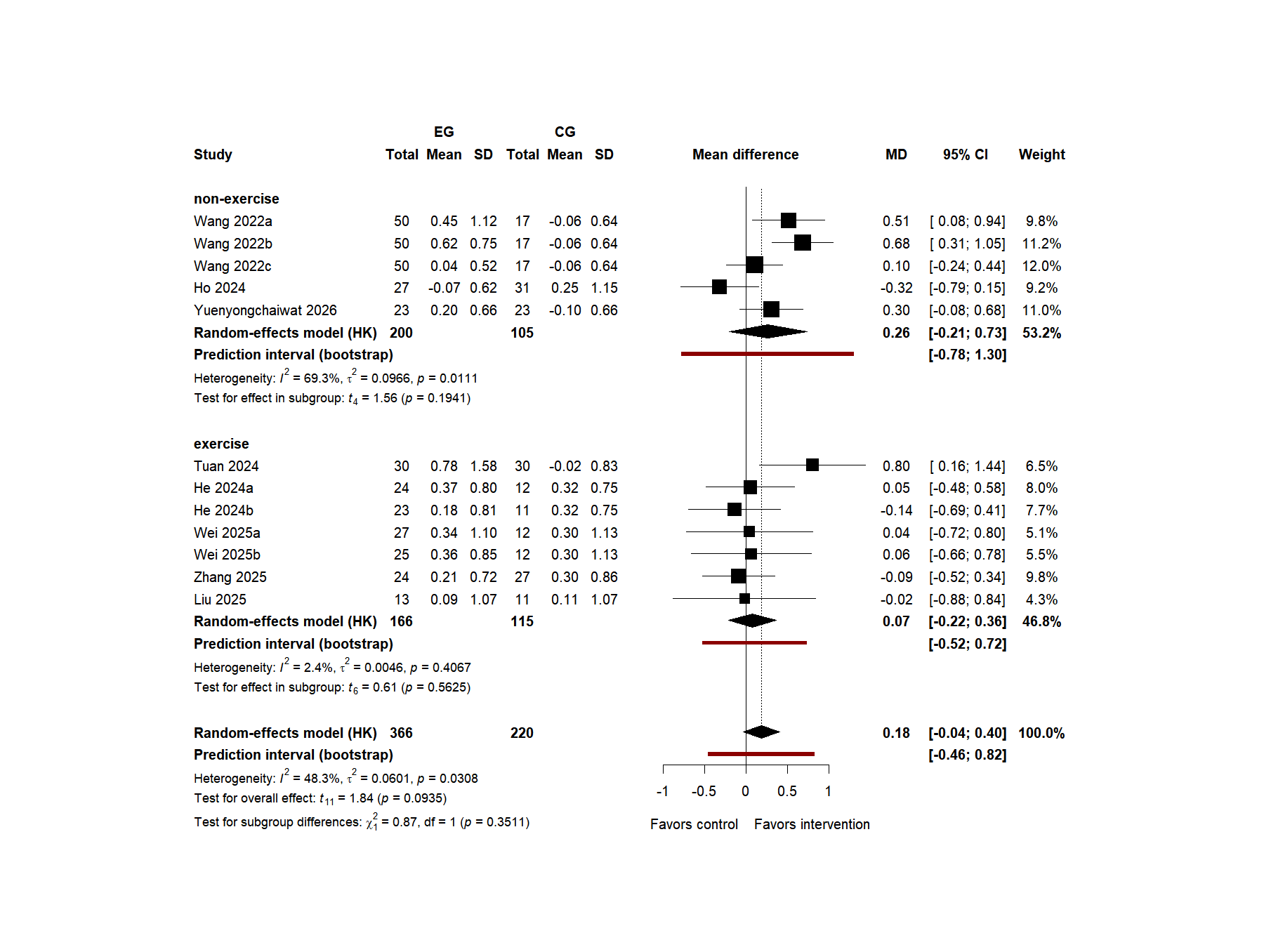
**

**1.2. Subgroup Analysis of 30-second Chair Stand Test (30CST) According to Control Type**

**
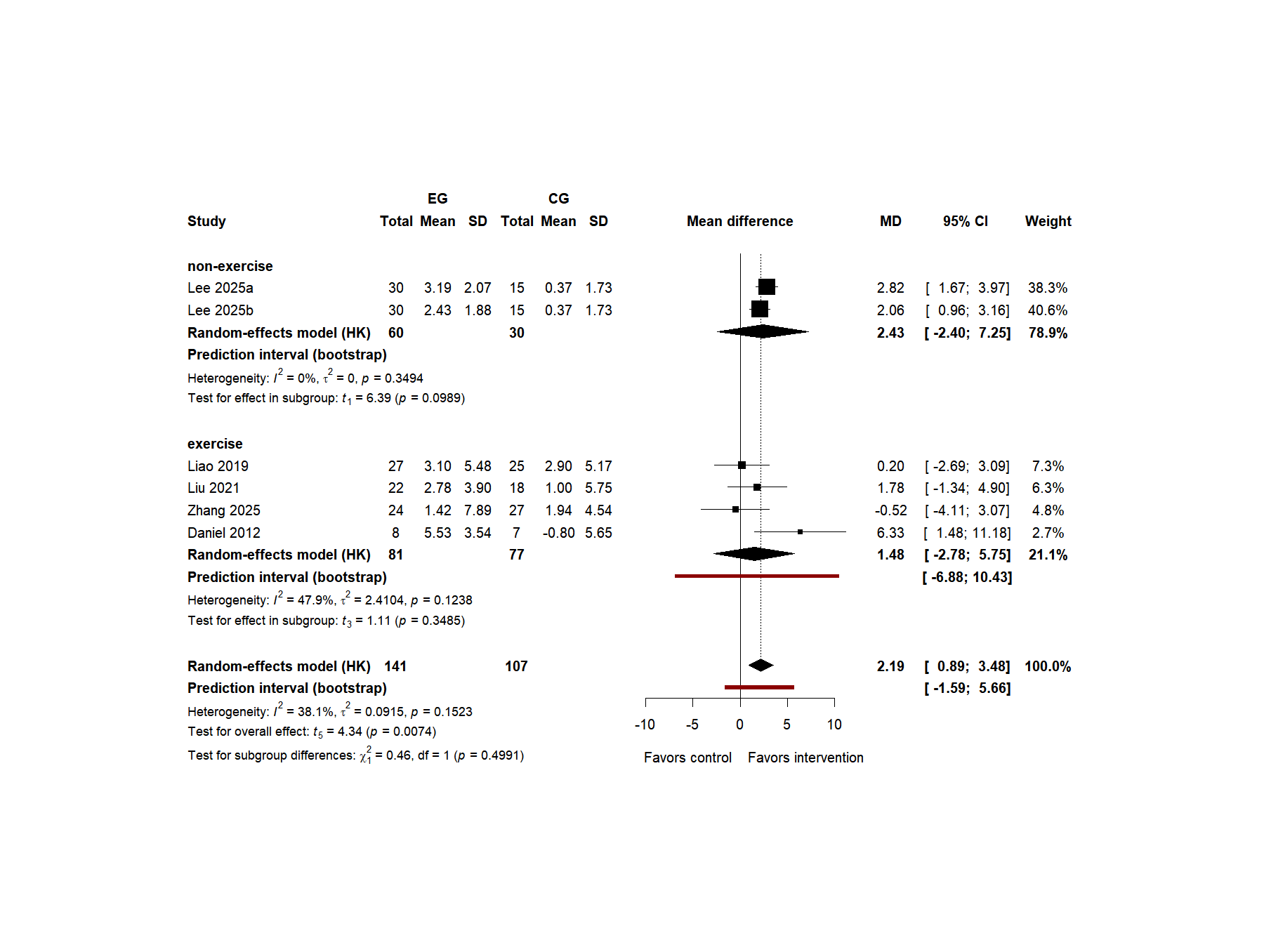
**

**1.3. Subgroup Analysis of Timed Up and Go Test (TUGT) According to Control Type**

**
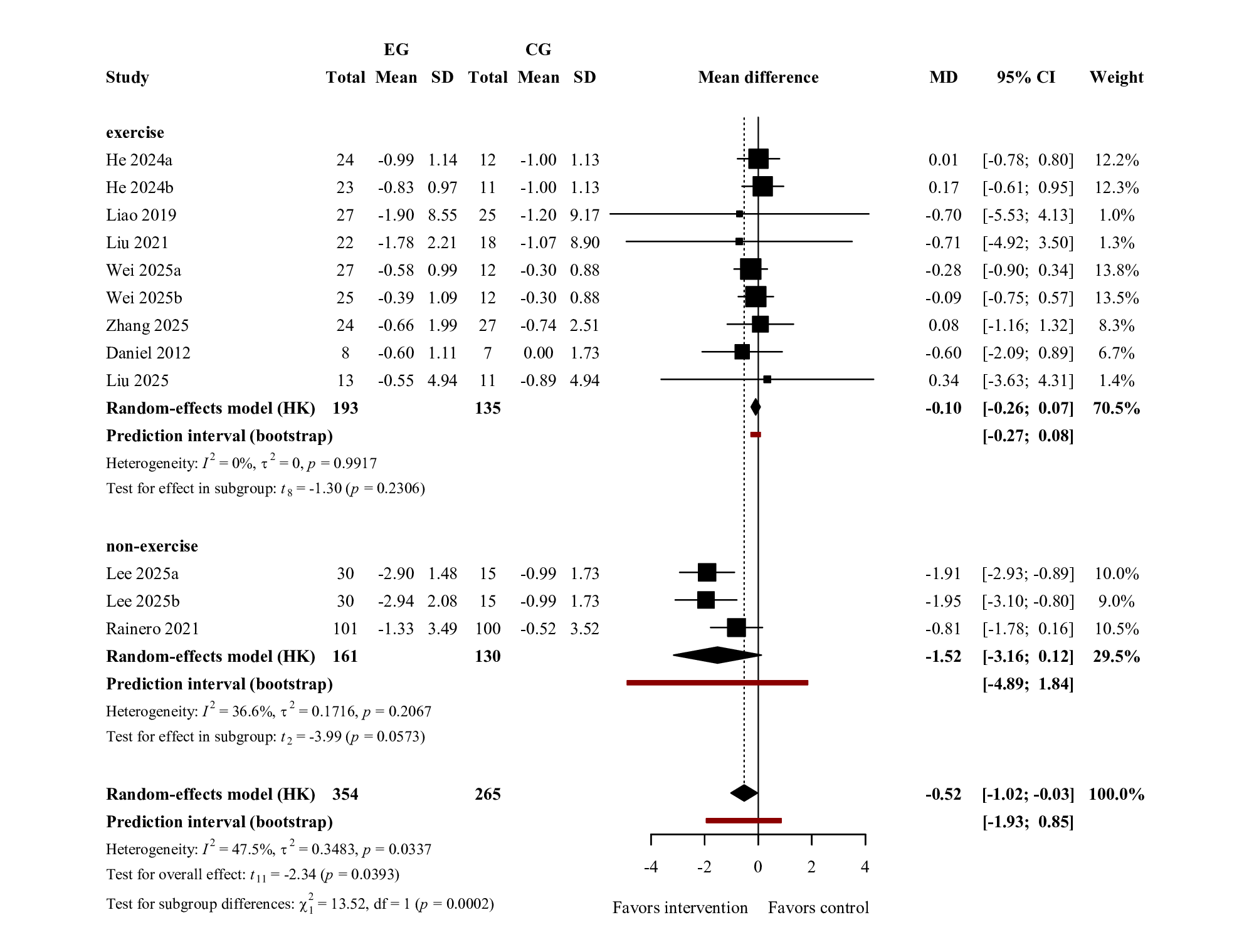
**

**Figure 1.4 Subgroup Analysis of Balance Based on Control Group Type**

**
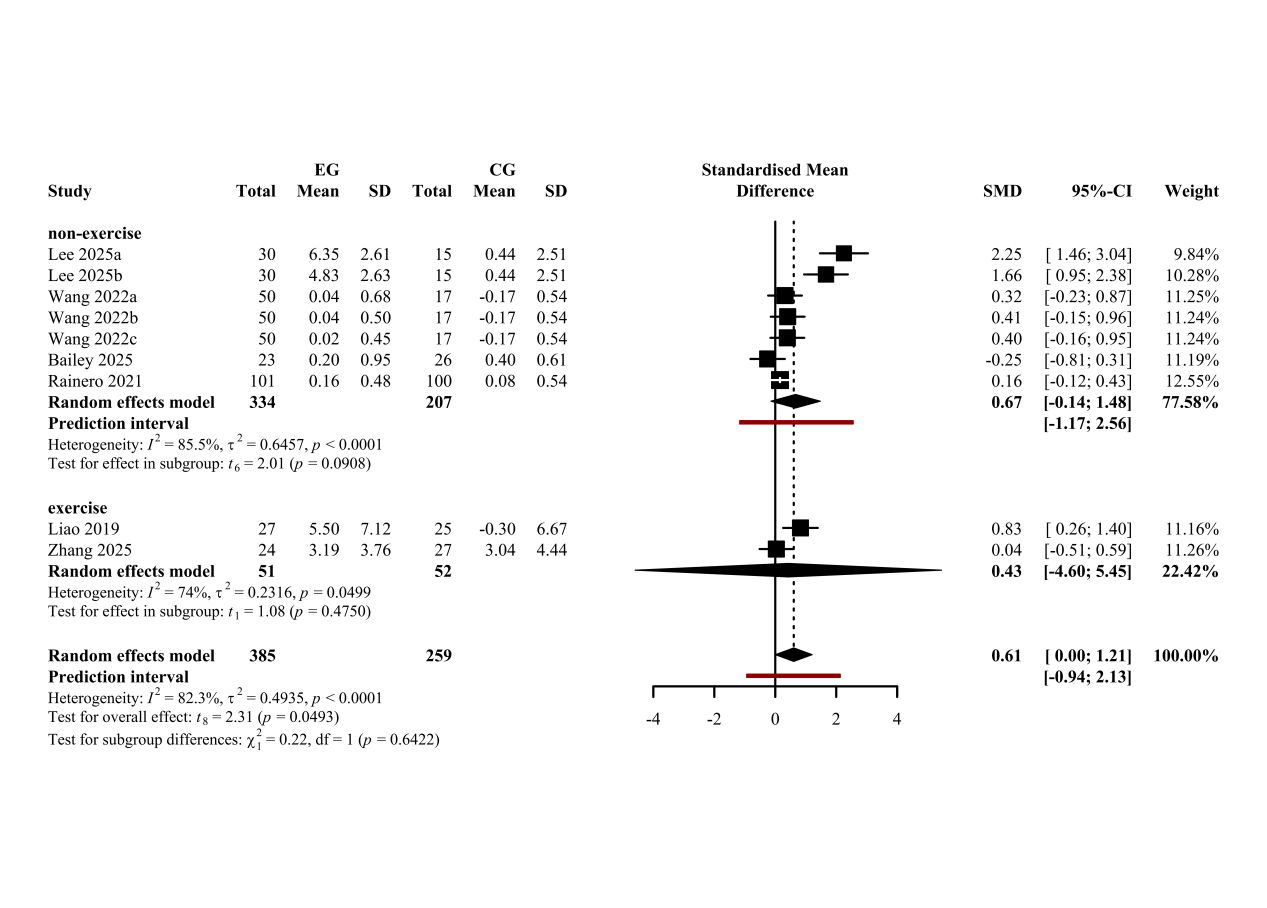
**

#### **Figure 2. Subgroup Analyses of Each Outcome According to T**ype of Digital Technology****

#### **Figure 2.1 Subgroup Analyses of Appendicular Skeletal Muscle Mass Index (ASMI) According to Type of Digital Technology**

#### **
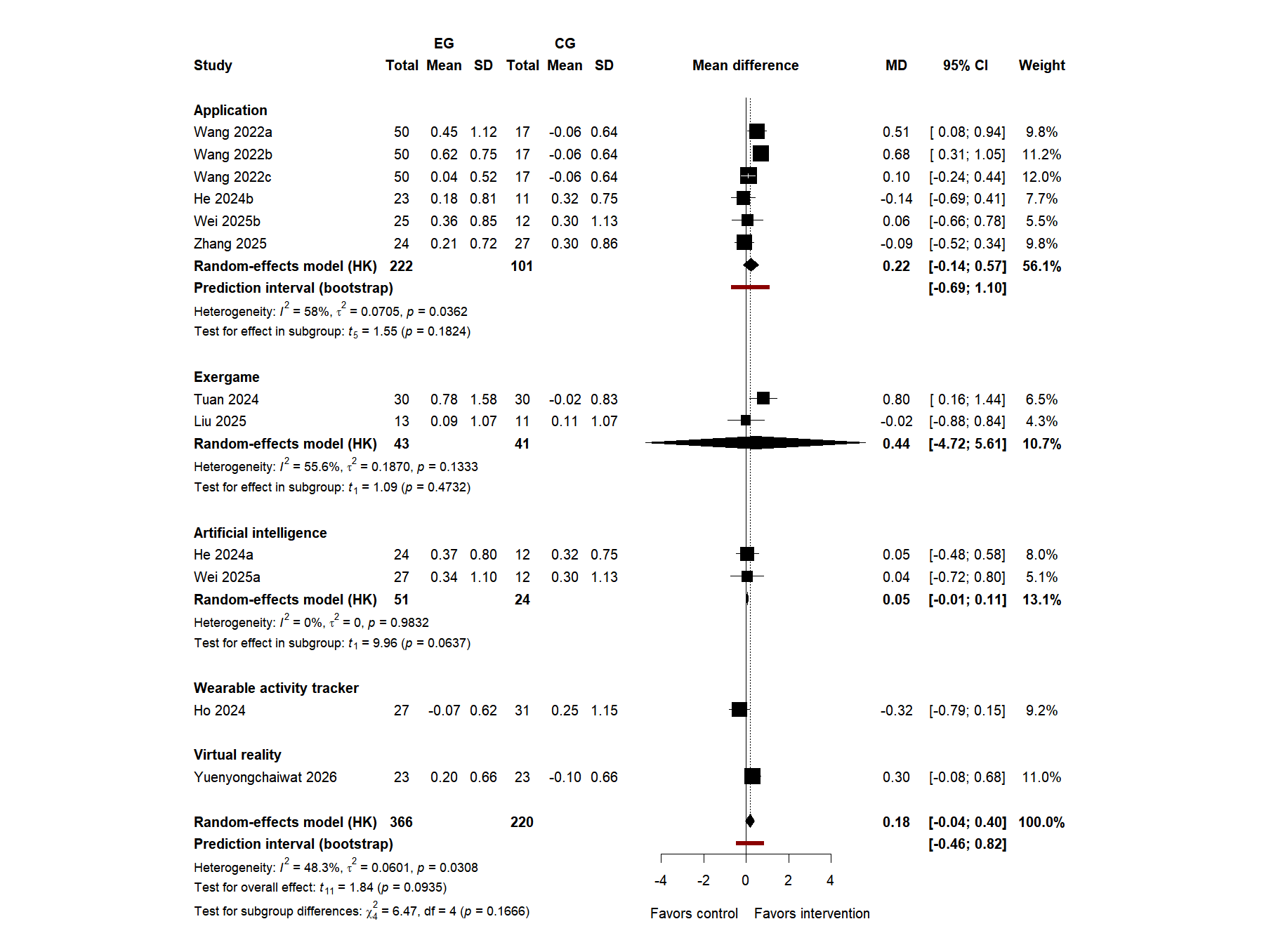
**

#### **Figure 2.2 Subgroup Analyses of 30-Second Chair Stand Test (30CST) According to Type of Digital Technology**

**
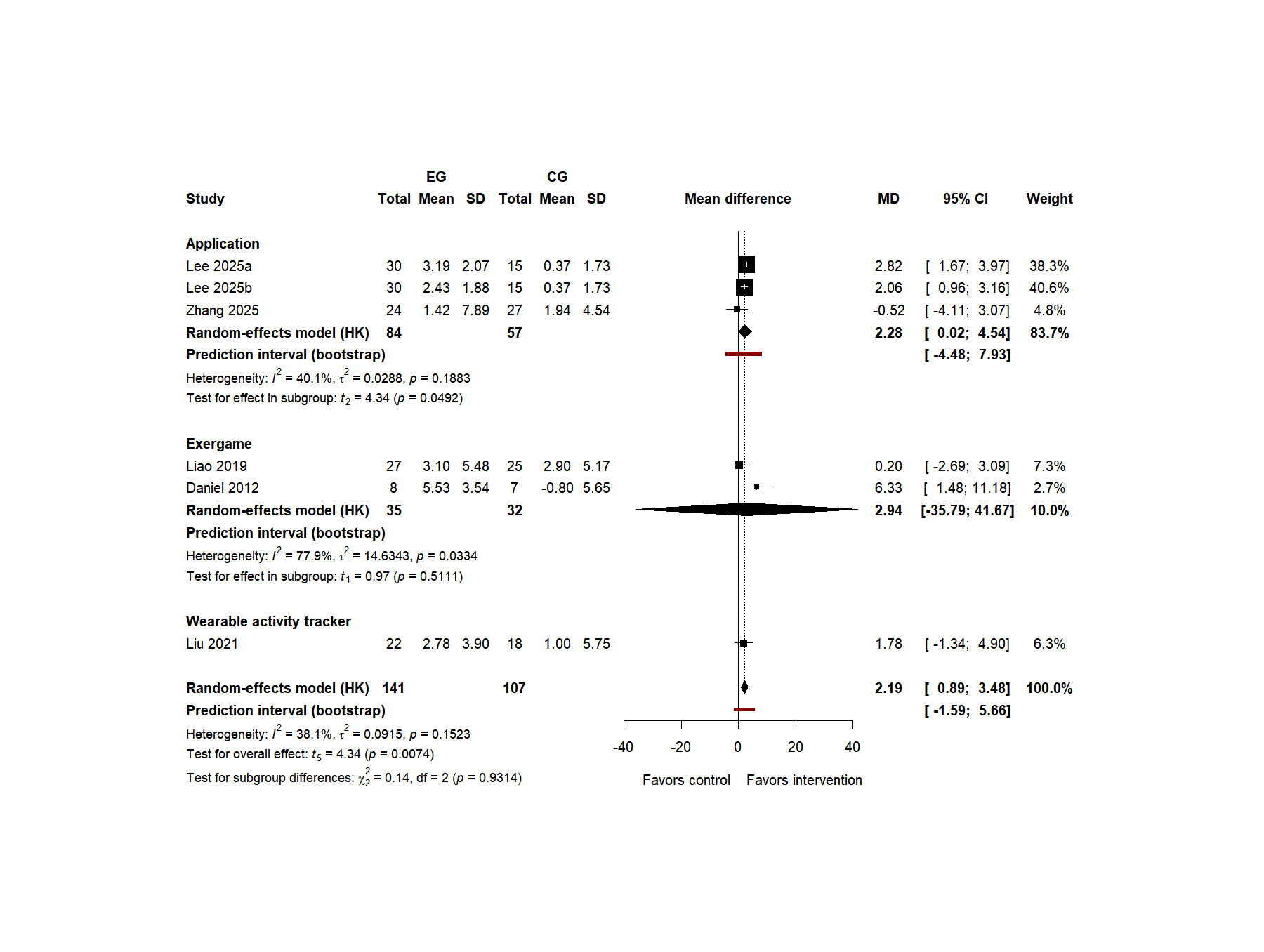
**

#### **Figure 2.3 Subgroup Analyses of Timed Up and Go Test (TUGT) According to Type of Digital Technology**

**
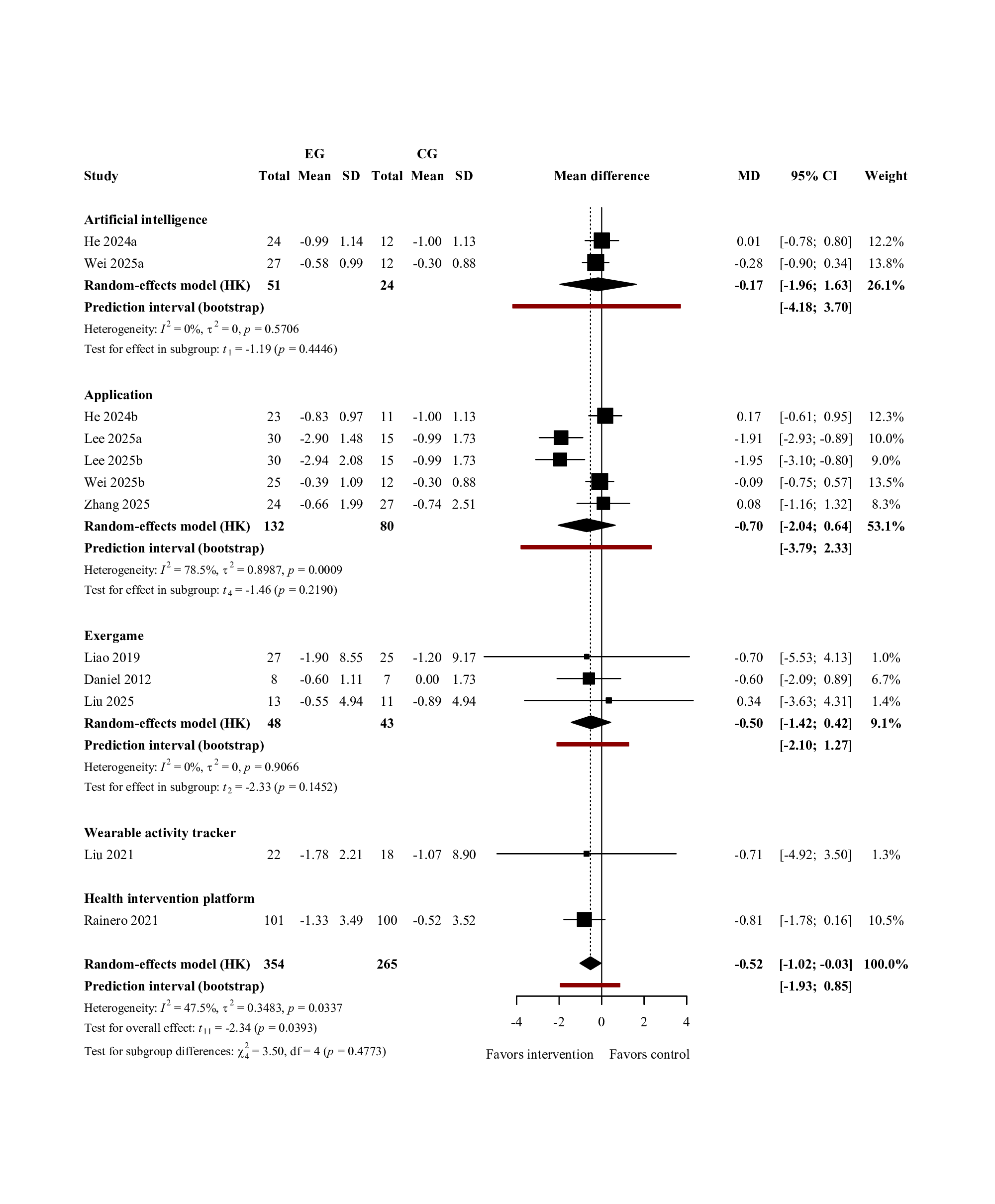
**

#### **Figure 2.4 Subgroup Analyses of Balance According to Type of Digital Technology**

**
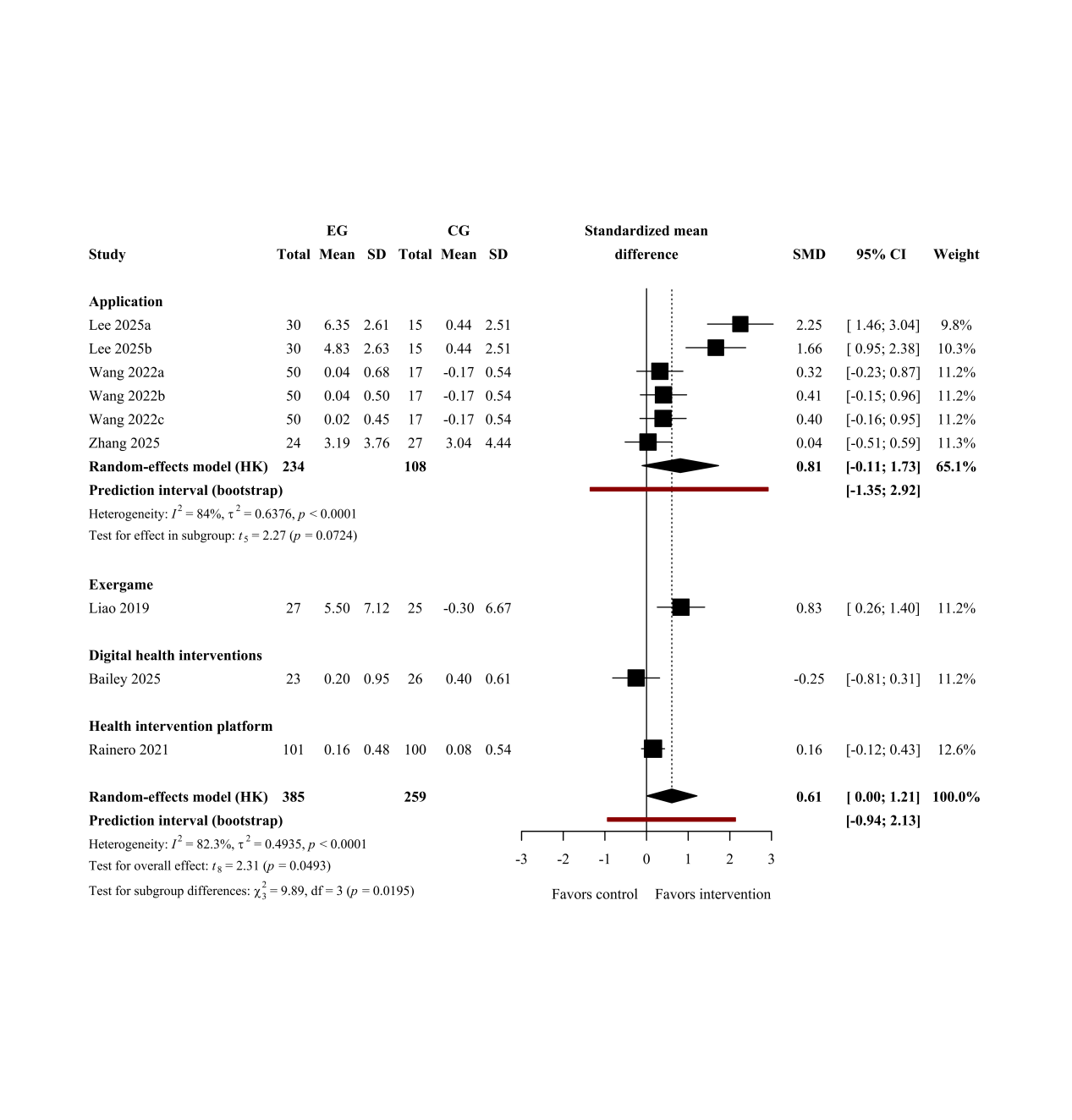
**

**Figure 3. Subgroup Analyses of Each Outcome According to Duration of Intervention**

**Figure 3.1. Subgroup Analysis of Appendicular Skeletal Muscle Mass Index (ASMI) According to Duration of Intervention**

**
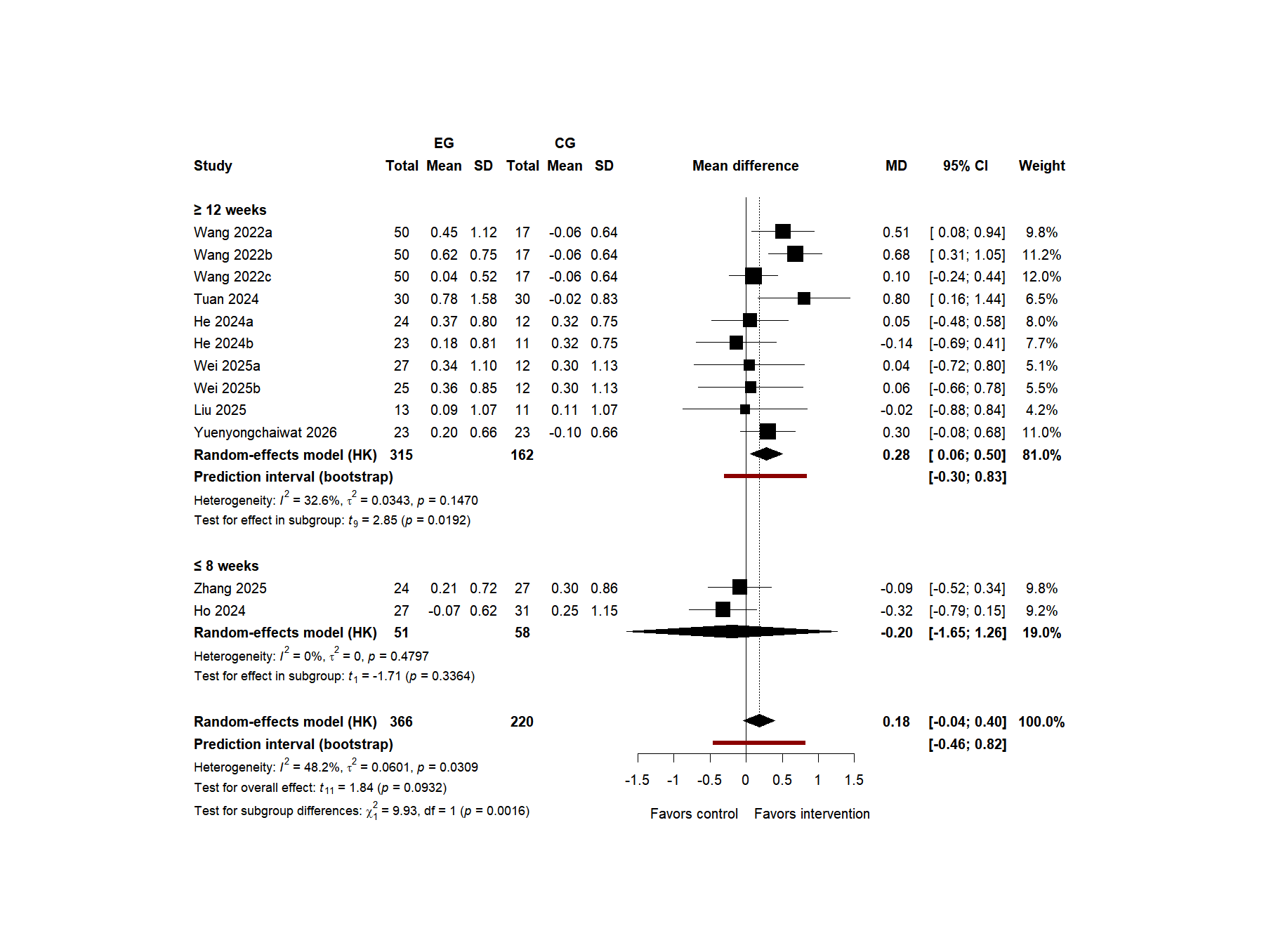
**

**Figure 3.2. Subgroup Analysis of 30-Second Chair Stand Test (30CST) According to Duration of Intervention**

**
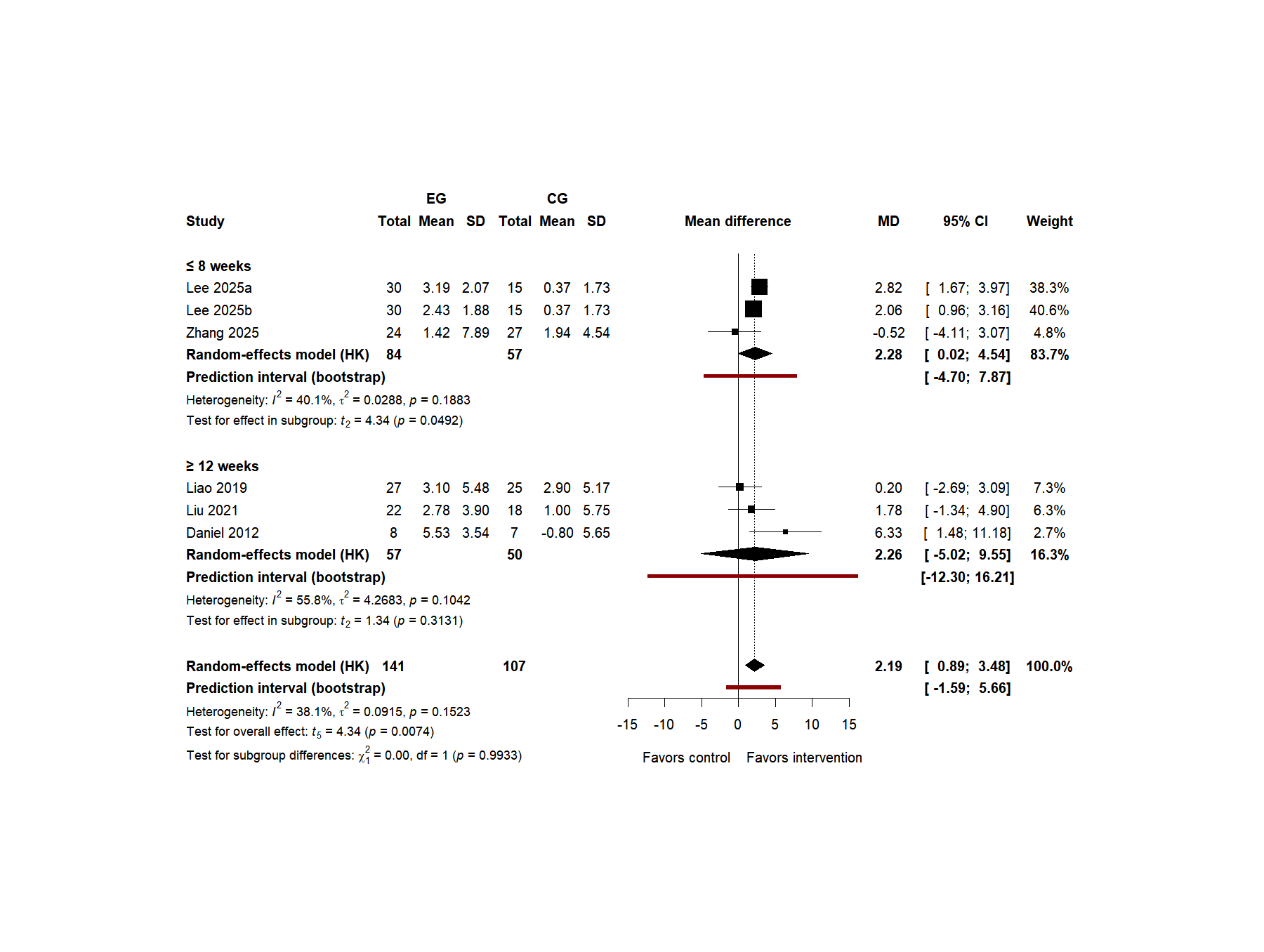
**

**Figure 3.3. Subgroup Analysis of Timed Up and Go Test (TUGT) According to Duration of Intervention**

**
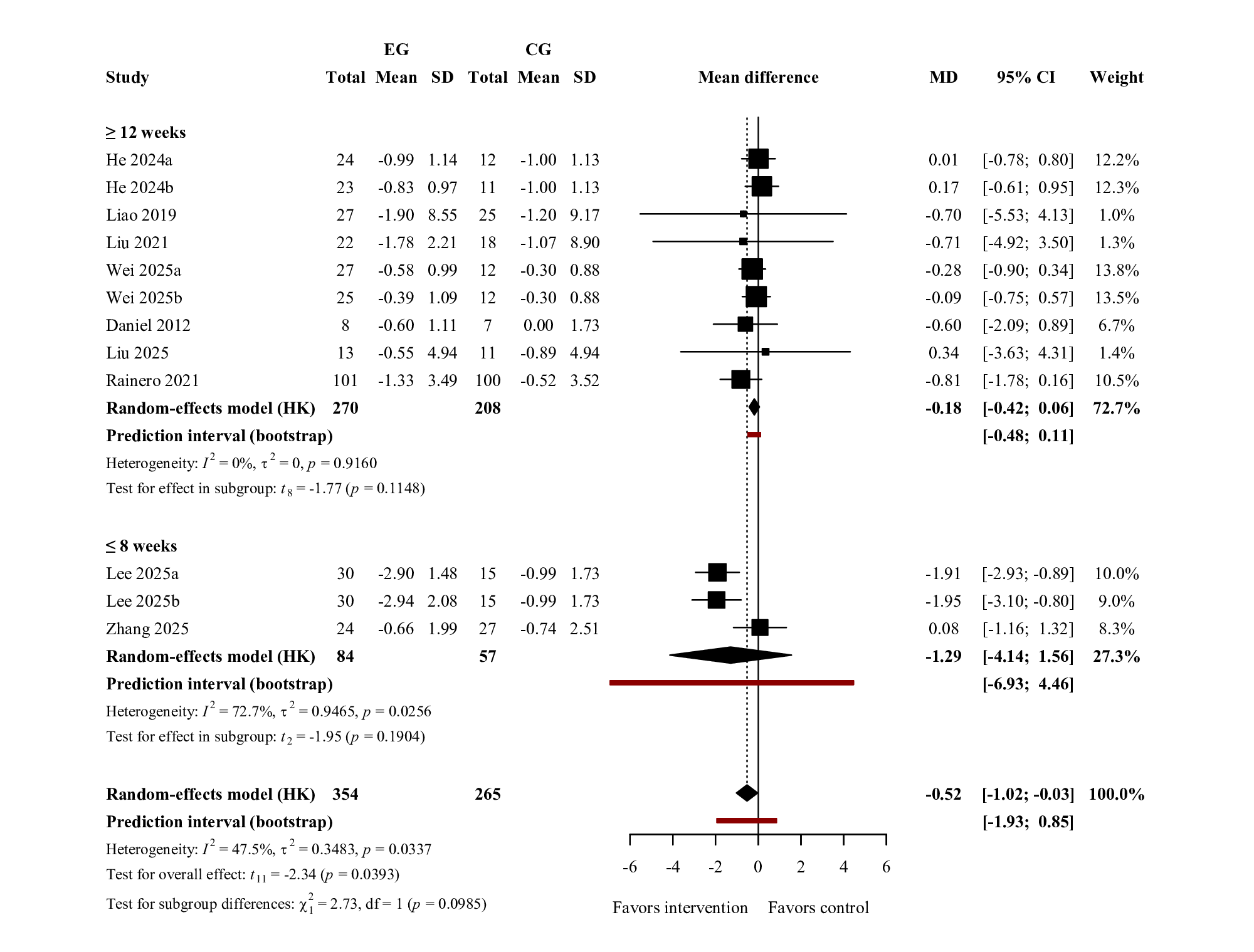
**

**Figure 3.5 Subgroup Analysis of Balance According to Duration of Intervention**

**
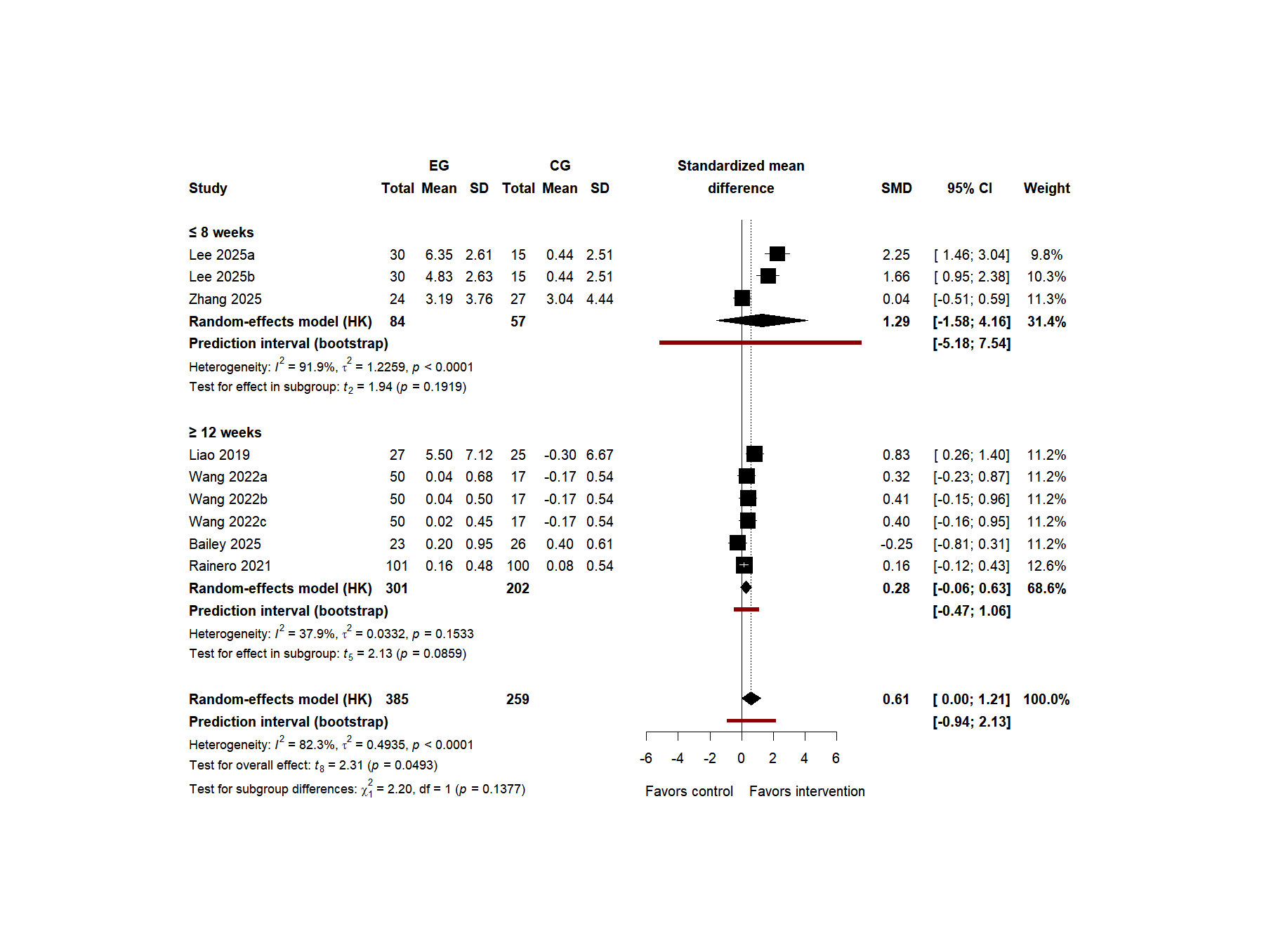
**

**Figure 4 Subgroup Analyses of Each Outcome According to Implementation Model: Synchronous versus Asynchronous**

**Figure 4.1 Subgroup Analyses of Appendicular Skeletal Muscle Mass Index (ASMI) According to Implementation Modality: Synchronous versus Asynchronous**

**
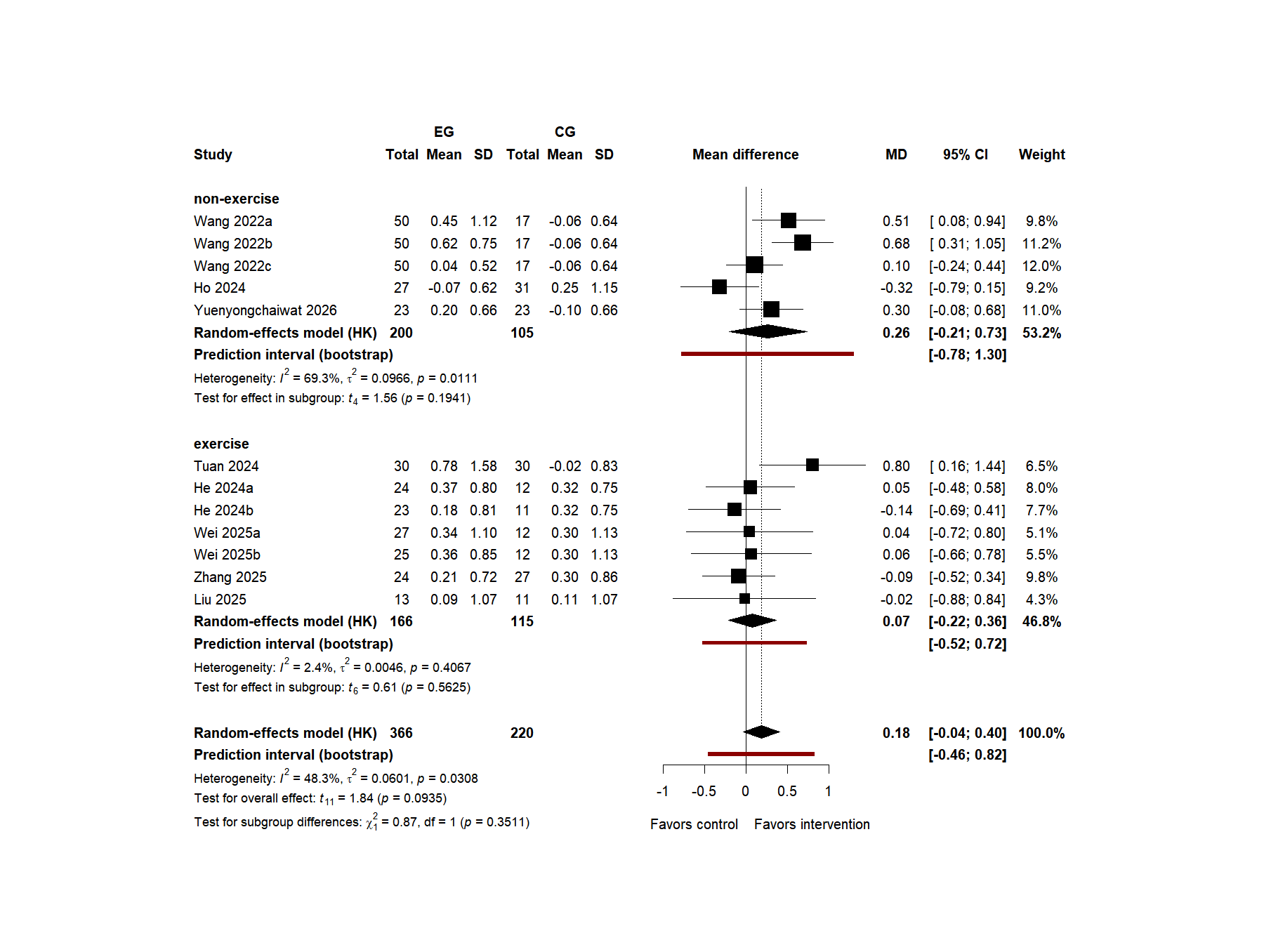
**

**Figure 4.2 Subgroup Analyses of 30-Second Chair Stand Test (30CST) According to Implementation Modality: Synchronous versus Asynchronous**

**
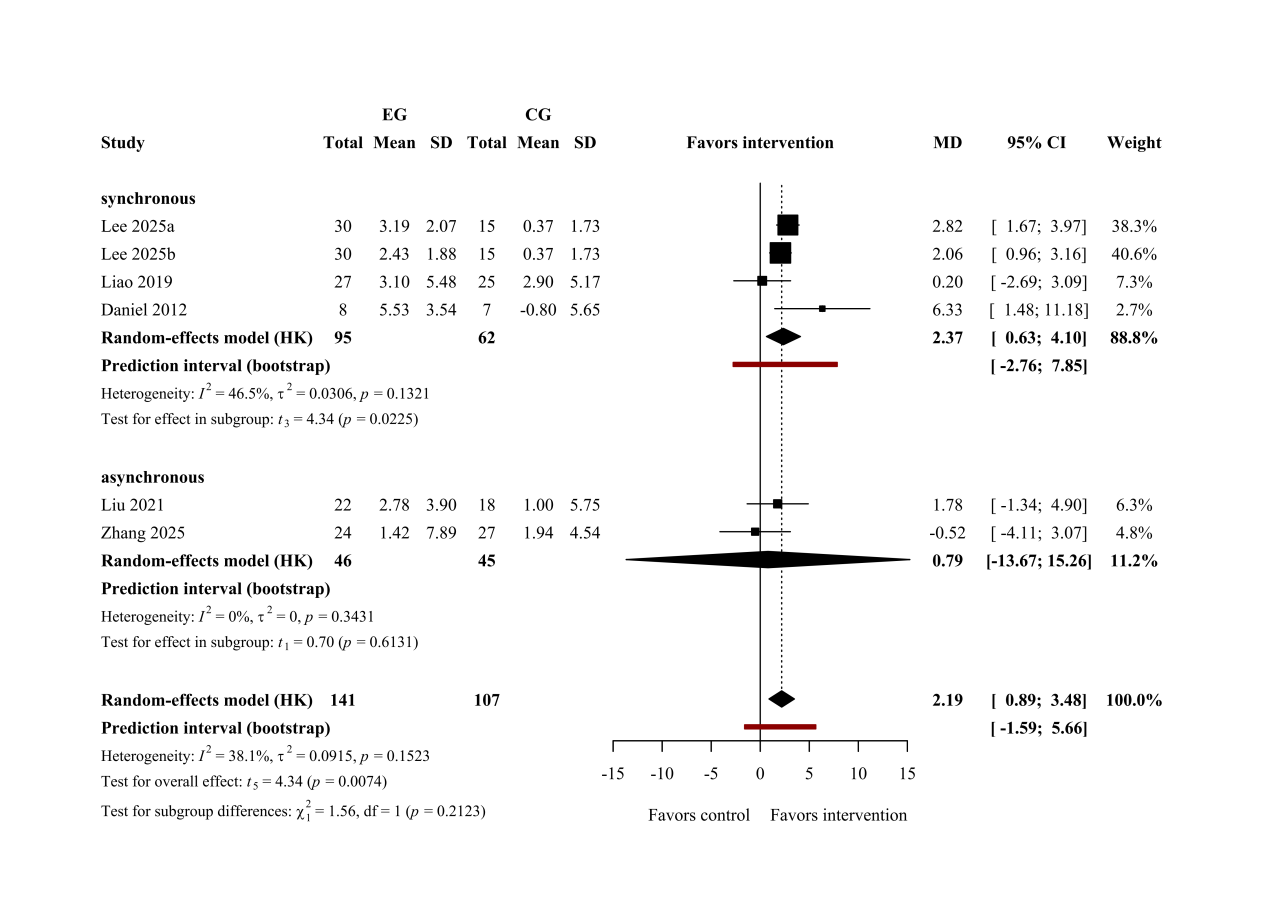
**

**Figure 4.3 Subgroup Analysis of Timed Up and Go Test (TUGT) According to Intervention Modality: synchronous versus asynchronous**

**
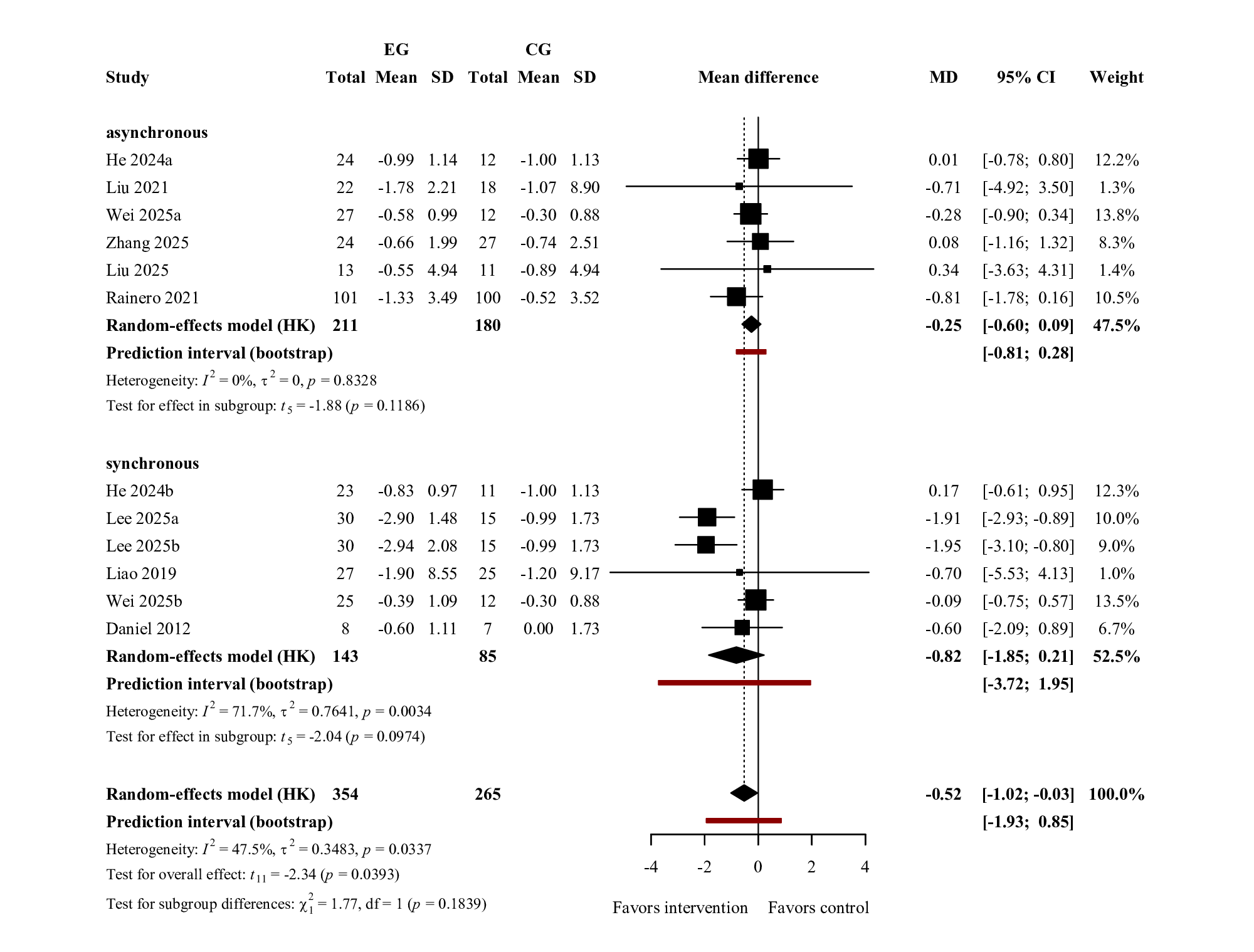
**

**Figure 4.4 Subgroup Analysis of Balance According to Intervention Modality: synchronous versus asynchronous**

**
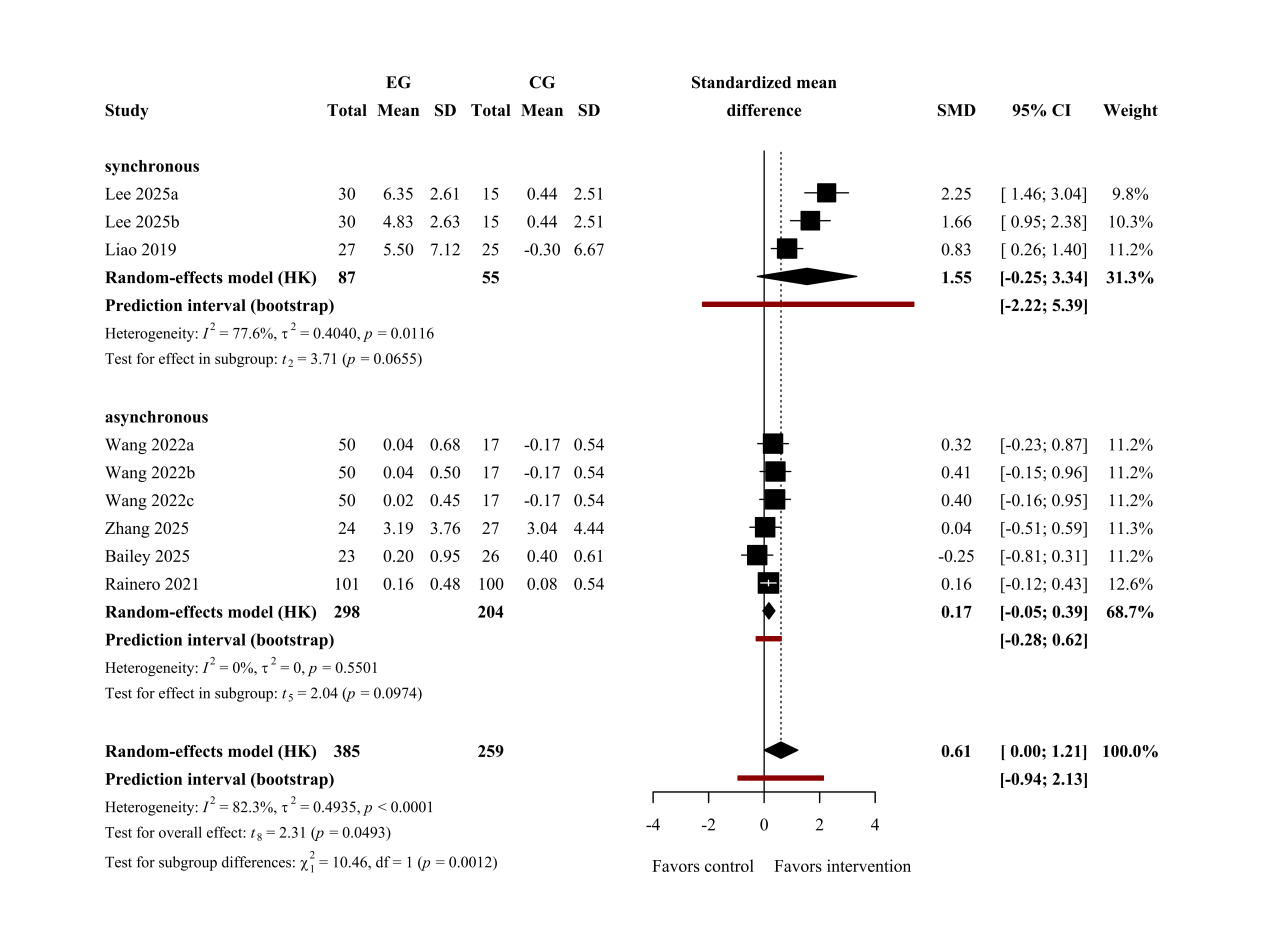
**

**Figure 5. Subgroup Analysis of Each Outcome According to Intervention Setting:home/remote versus center-based**

**Figure 5.1 Subgroup Analysis of Appendicular Skeletal Muscle Mass Index (ASMI) According to Intervention Setting: home/remote versus center-based**

**
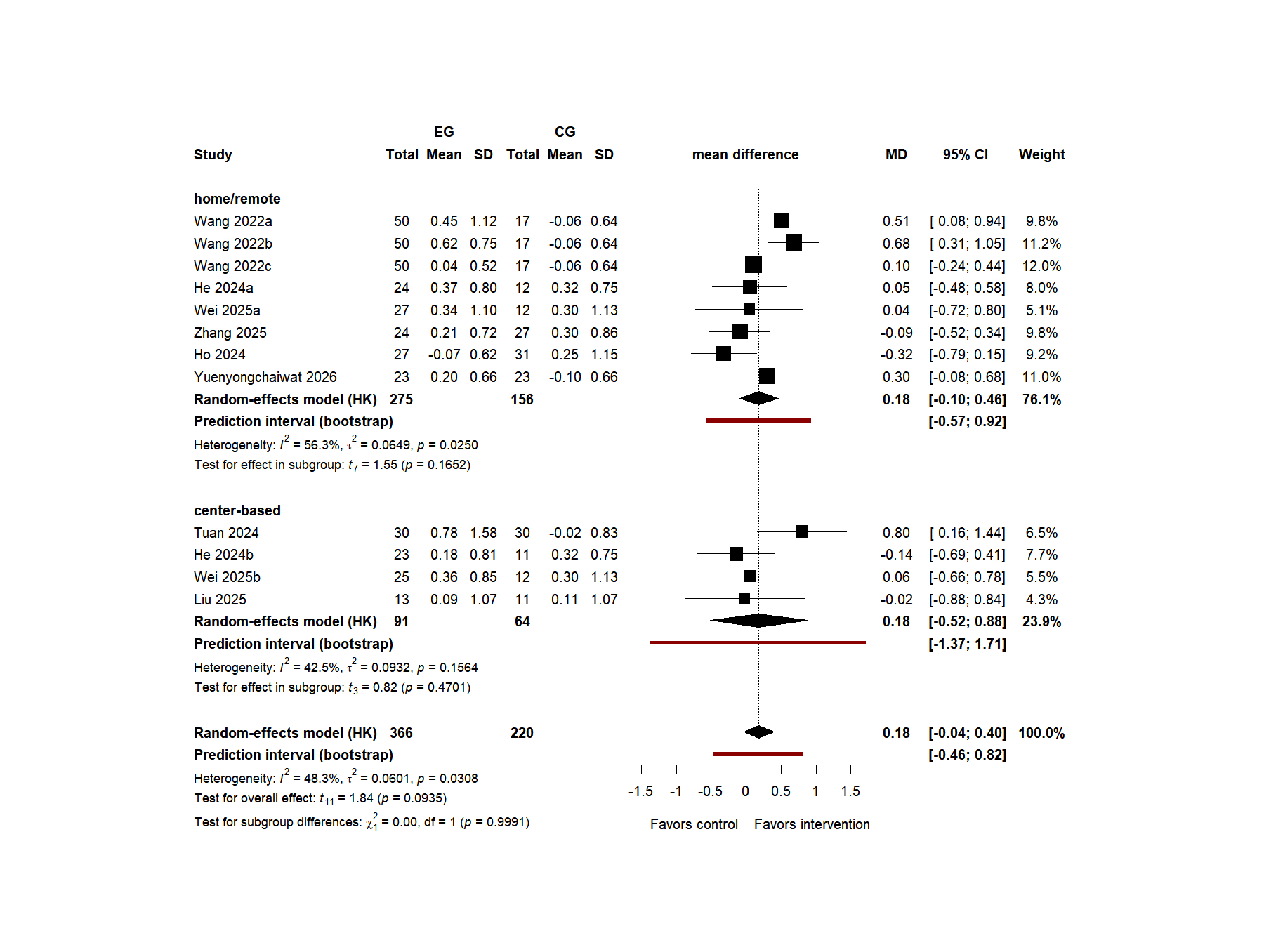
**

**Figure 5.2 Subgroup Analysis of Timed Up and Go Test (TUGT) According to Intervention Setting: home/remote versus center-based**

**
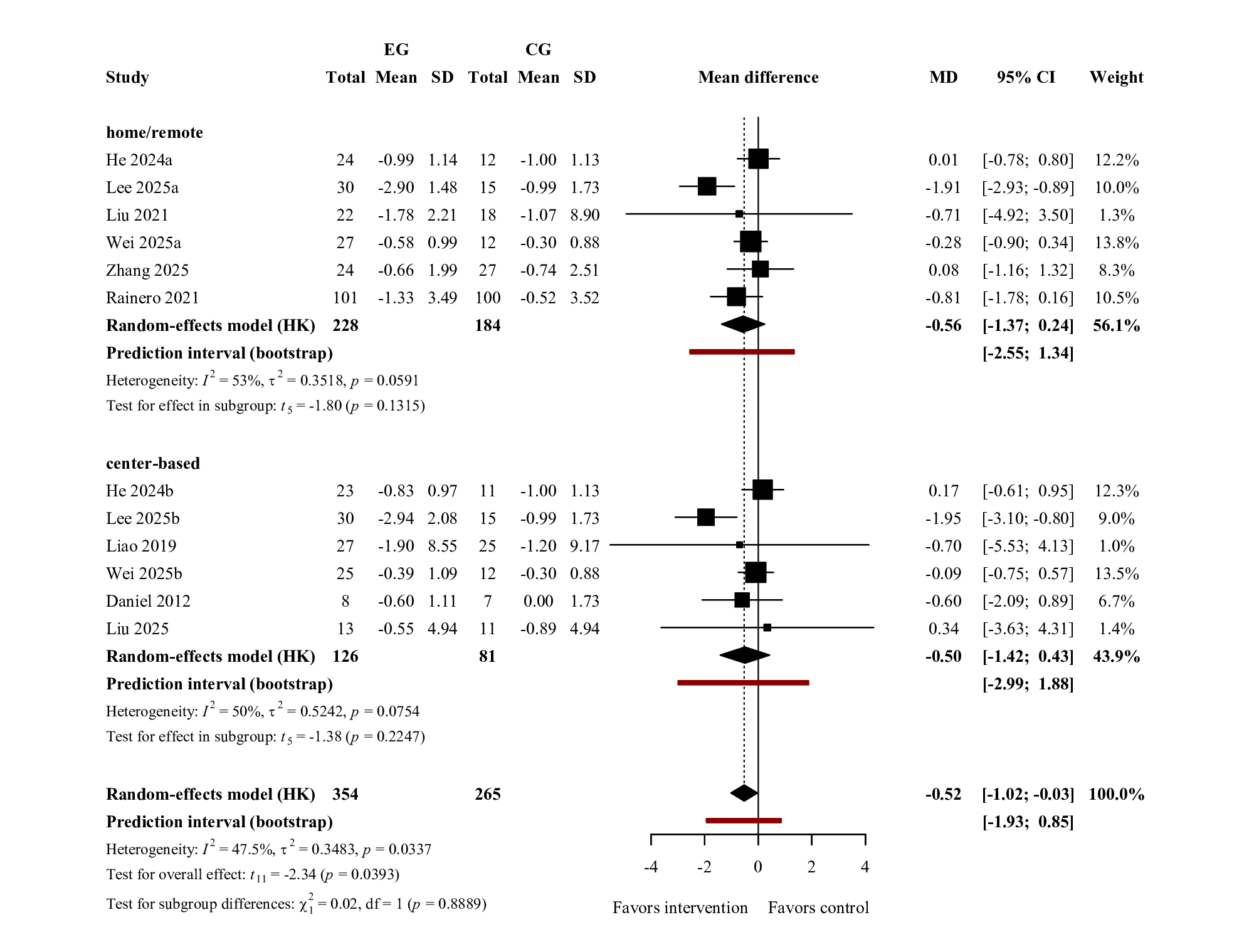
**

**Figure 5.3 Subgroup Analysis of Balance According to Intervention Setting: home/remote versus center-based**

**
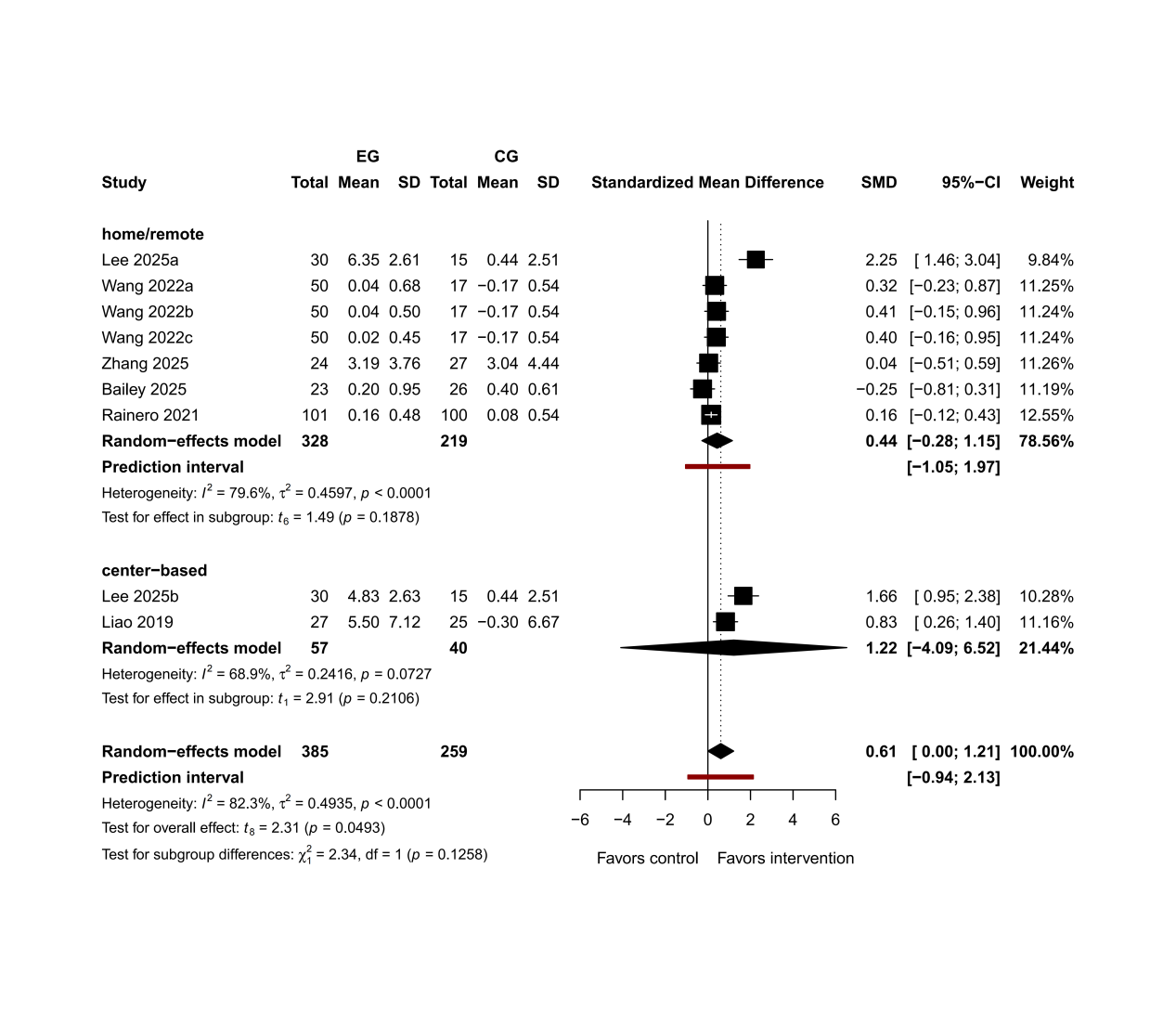
**

**Figure 5.3 Subgroup Analysis of 30-Second Chair Stand Test (30CST) According to Intervention Setting: home/remote versus center-based**

**
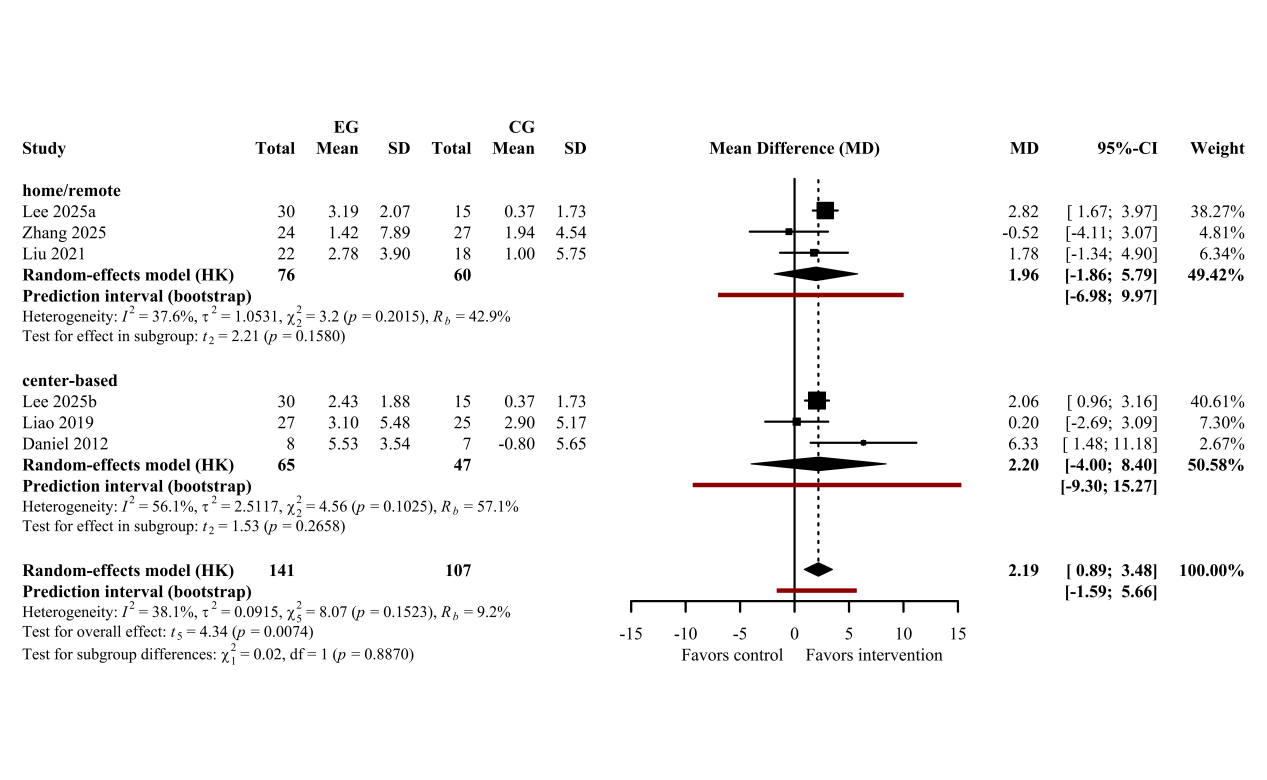
**
