# Supplementary material for: Effectiveness of Digital Health Interventions in Older Adults With Frailty and Sarcopenia: Systematic Review and Meta‐Analysis of Randomized Controlled Trials
Source: J Med Internet Res. 2026 May 11;28:e88374. doi: 10.2196/88374 (PMC13161750; doi:10.2196/88374)

**Sensitivity Analysis**

Note: CG: control group; EG: experimental group; HK: Hartung-Knapp (method); MD:mean difference; SMD: standardized mean difference; Labels a, b and c denote distinct intervention arms from the same multi-arm trial. To avoid double counting, the sample size of the shared control group was split equally between intervention arms (Wang 2022a/b/c, Lee 2025a/b).

**Figure S1.** Sensitivity Analysis of 5 sit-to-stand tests Meta-Analysis.


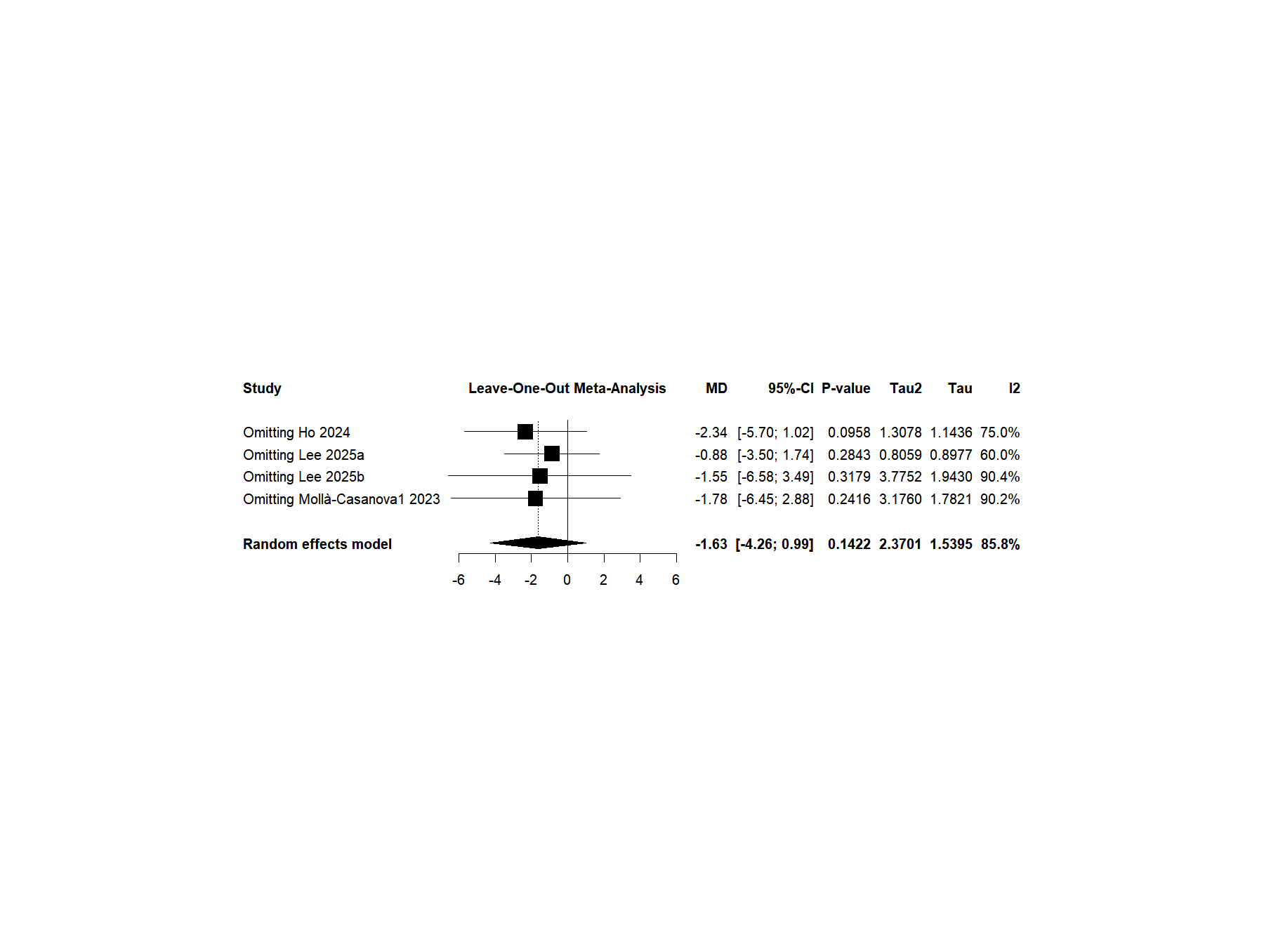


**Figure S2.** Sensitivity Analysis of Balance Meta-Analysis.


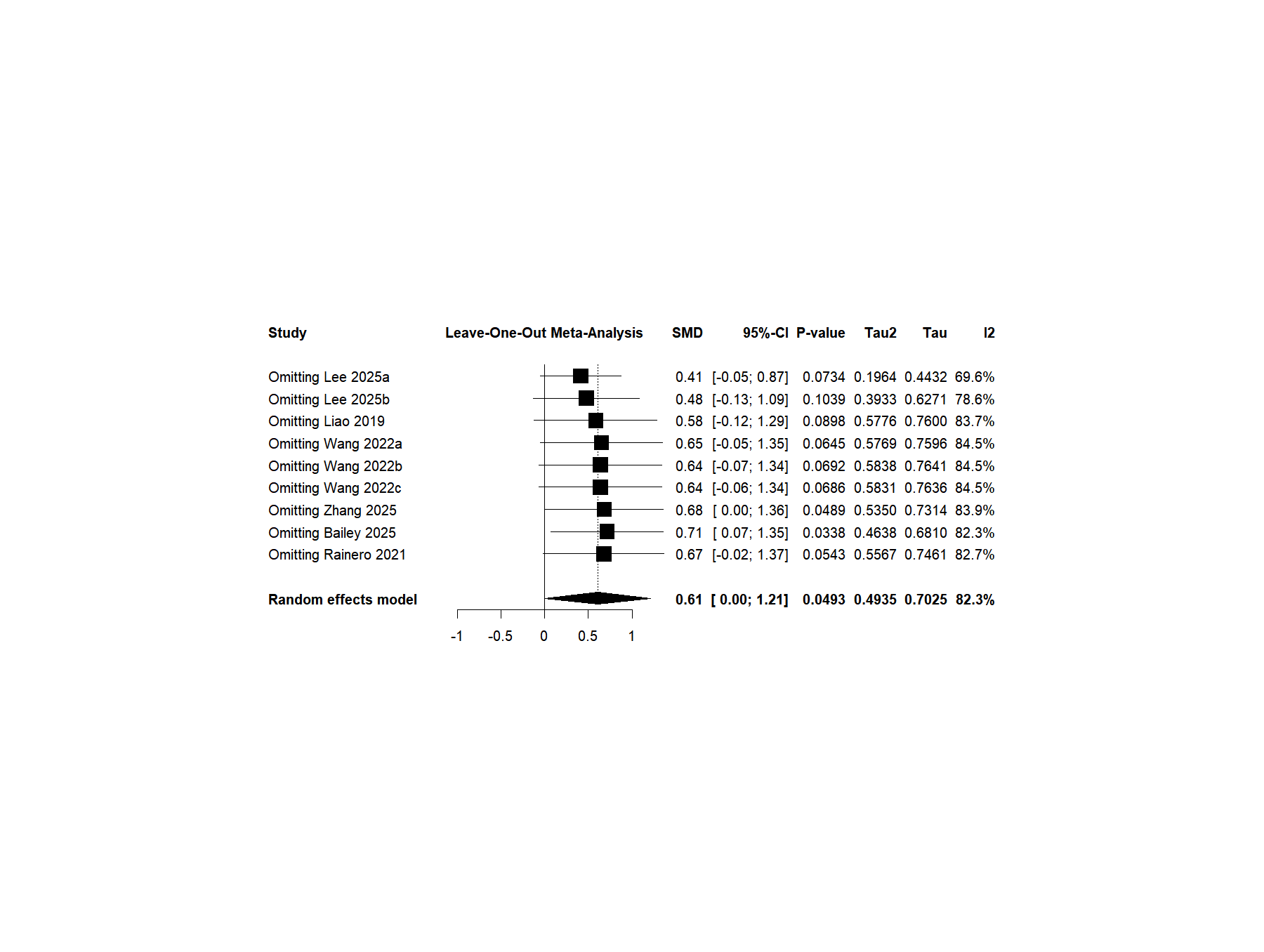

Supplement: Multimedia Appendix 5 [file jmir-v28-e88374-s005.docx]
