# Supplementary material for: Effectiveness of Digital Health Interventions in Older Adults With Frailty and Sarcopenia: Systematic Review and Meta‐Analysis of Randomized Controlled Trials
Source: J Med Internet Res. 2026 May 11;28:e88374. doi: 10.2196/88374 (PMC13161750; doi:10.2196/88374)

**Funnel plots**

1.1 Funnel plot of Grip Strength


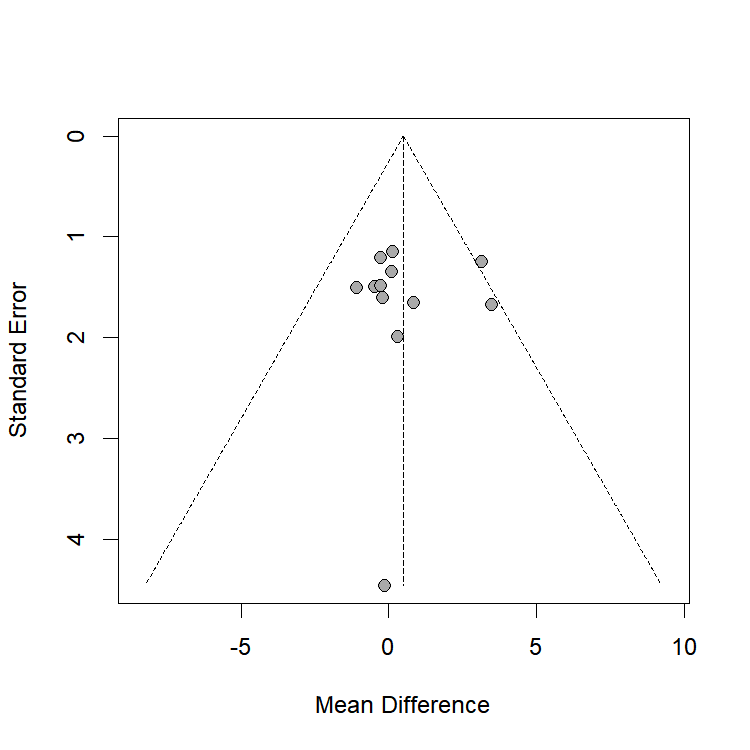


1.2 Funnel plot of Appendicular Skeletal Muscle Mass Index (ASMI)


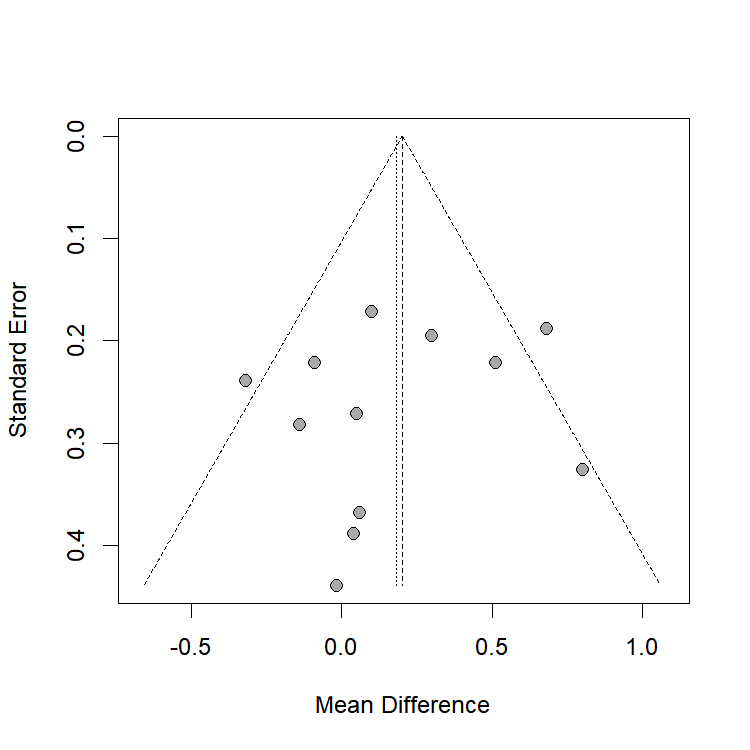


1.3 Funnel plot of Gait Speed


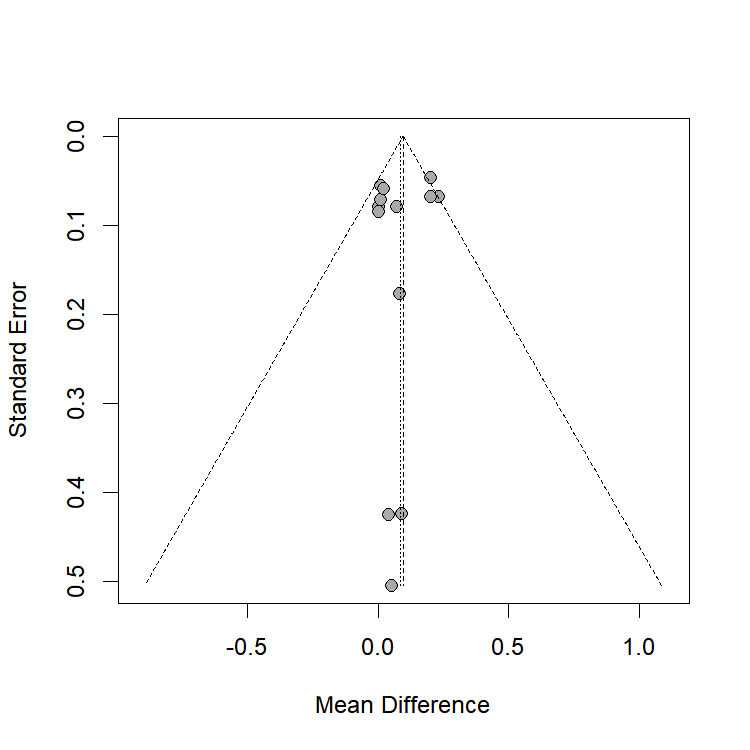


1.4 Funnel plot of Timed Up and Go Test (TUGT)


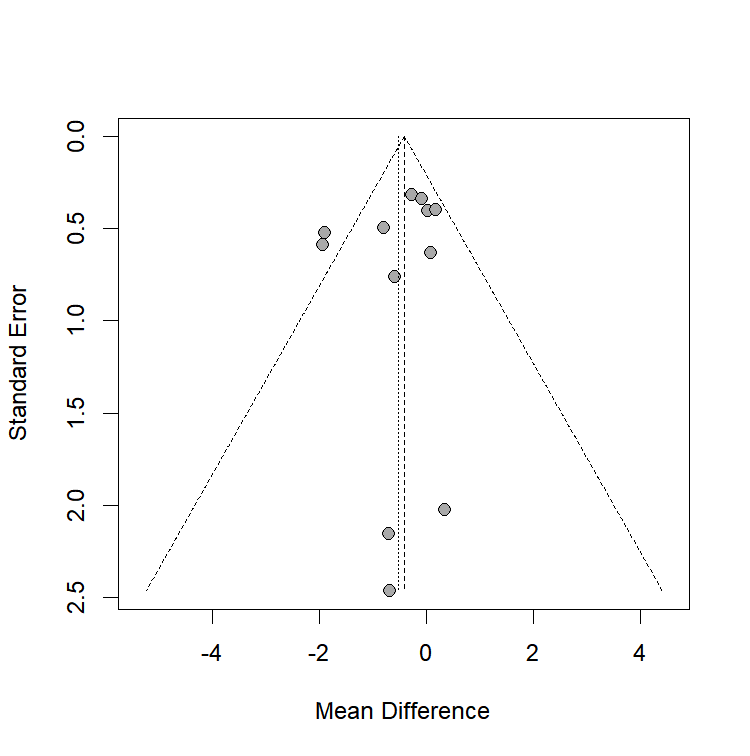

Supplement: Multimedia Appendix 6 [file jmir-v28-e88374-s006.docx]
